# Smc5/6 Coordinates Formation and Resolution of Joint Molecules with Chromosome Morphology to Ensure Meiotic Divisions

**DOI:** 10.1371/journal.pgen.1004071

**Published:** 2013-12-26

**Authors:** Alice Copsey, Shangming Tang, Philip W. Jordan, Hannah G. Blitzblau, Sonya Newcombe, Andrew Chi-ho Chan, Louise Newnham, Zhaobo Li, Stephen Gray, Alex D. Herbert, Prakash Arumugam, Andreas Hochwagen, Neil Hunter, Eva Hoffmann

**Affiliations:** 1 MRC Genome Damage and Stability Centre, University of Sussex, Brighton, United Kingdom; 2 Howard Hughes Medical Institute, University of California, Davis, Davis, California, United States of America; 3 Department of Microbiology & Molecular Genetics, University of California, Davis, Davis, California, United States of America; 4 Whitehead Institute for Biomedical Research, Cambridge, Massachusetts, United States of America; 5 Department of Life Sciences, University of Warwick, Coventry, United Kingdom; 6 Department of Biology, New York University, New York, New York, United States of America; 7 Department of Molecular & Cellular Biology, University of California, Davis, Davis, California, United States of America; 8 Department of Cell Biology & Human Anatomy, University of California, Davis, Davis, California, United States of America; National Cancer Institute, United States of America

## Abstract

During meiosis, Structural Maintenance of Chromosome (SMC) complexes underpin two fundamental features of meiosis: homologous recombination and chromosome segregation. While meiotic functions of the cohesin and condensin complexes have been delineated, the role of the third SMC complex, Smc5/6, remains enigmatic. Here we identify specific, essential meiotic functions for the Smc5/6 complex in homologous recombination and the regulation of cohesin. We show that Smc5/6 is enriched at centromeres and cohesin-association sites where it regulates sister-chromatid cohesion and the timely removal of cohesin from chromosomal arms, respectively. Smc5/6 also localizes to recombination hotspots, where it promotes normal formation and resolution of a subset of joint-molecule intermediates. In this regard, Smc5/6 functions independently of the major crossover pathway defined by the MutLγ complex. Furthermore, we show that Smc5/6 is required for stable chromosomal localization of the XPF-family endonuclease, Mus81-Mms4^Eme1^. Our data suggest that the Smc5/6 complex is required for specific recombination and chromosomal processes throughout meiosis and that in its absence, attempts at cell division with unresolved joint molecules and residual cohesin lead to severe recombination-induced meiotic catastrophe.

## Author Summary

Meiosis is a specialized cell division that exactly halves the number of chromosomes transmitted from each parent to their offspring via gamete cells (such as sperm and eggs). This requires that matching (‘homologous’) chromosomes associate and then separate into different cells such that each gamete contains exactly one complete set of chromosomes. In many organisms, this sequence of events is facilitated by the induction and repair of chromosome breaks via a process called homologous recombination. As homologous chromosomes engage in recombination, matching DNA strands between broken and intact template chromosomes become intertwined in repair intermediates called Joint Molecules. In this study, we show that a highly conserved protein complex called the Structural Maintenance of Chromosomes 5/6 (Smc5/6) complex is important for regulating the choice of recombination template as well as for the resolution of Joint Molecules that is required for chromosomes to separate. Even though Joint Molecules remain unresolved in mutants that lack normal Smc5/6 function, cells still attempt to separate chromosomes and meiosis becomes catastrophic. Thus, Smc5/6 mutants have a two-fold defect: accumulation of unresolved Joint Molecules and a failure to stall meiosis in order to remove these structures.

## Introduction

Sexually reproducing organisms reduce their genomic content by half in the gametes such that the normal chromosome copy number is restored in the zygote. To achieve this, homologous chromosomes (homologs) have to pair and then segregate to opposite spindle poles at the first division of meiosis. In many organisms, homolog pairing and segregation depends upon the developmental induction of hundreds of double-strand breaks (DSBs) throughout the genome (150–300 DSBs in yeasts and mammals) [1]. High levels of DSBs are necessary for homologs to pair efficiently along their entire lengths [2]. Moreover, a subset of DSB repair events lead to crossover formation. These reciprocal exchanges between homologs combine with sister-chromatid cohesion to form chiasmata, the physical connections that aid bi-orientation of homologs on the meiosis I spindle. Homolog separation at anaphase I thus requires the release of sister chromatid cohesion between chromosome arms. However, centromere cohesion is specifically protected to allow biorientation and accurate segregation of sister chromatids on the meiosis-II spindles [3]–[5].

Meiotic recombination is highly regulated and temporally coordinated with the meiotic cell cycle. Crossover-specific joint molecule intermediates (JMs) are formed during midprophase I of meiosis (‘thick threads’, pachytene), when homologous chromosomes are highly compacted and paired along their entire length by the synaptonemal complex. JMs are resolved into crossovers upon pachytene exit when a dedicated resolving process becomes activated by polo-like kinase [6]–[8]. In contrast, most noncrossovers arise during prophase I, independently of known resolving nucleases via a process termed synthesis-dependent single-strand annealing [8], [9].

The formation of JMs is guided by the RecQ-family DNA helicase Sgs1/BLM, which limits the formation of aberrant JM structures, such as those that interconnect 3 or 4 chromatids instead of the normal two [10], [11]. Resolution of aberrant JMs requires the activities of structure-selective nucleases, Mus81-Mms4, Slx1-Slx4 and Yen1 [11]–[14]. Sgs1 together with type-I topoisomerase, Top3, and accessory factor, Rmi1, defines a potent double Holliday junction (dHJ) “dissolving” enzyme that specifically promotes noncrossover formation [15], [16]. At pre-crossover sites, this dissolution activity must be attenuated in order to ensure efficient crossing over.

In budding yeast, a majority of crossovers are formed via a dedicated pathway defined by the conserved, meiosis-specific MutS complex, MutSγ (Msh4–Msh5) that is predicted to encircle and thereby stabilize JMs [17]–[20]. From extensive studies, we know that components of the MutSγ pathway promote the formation of stable JMs, Single End Invasions (SEIs) and dHJs, and protect them from being dissociated by Sgs1 [10], [20], [21]. Subsequent resolution of dHJs into crossovers requires the DNA mismatch repair factors, Exo1 and the predicted endonuclease activity of MutLγ, a complex of the MutL homologs Mlh1 and Mlh3 [22], [23].

In *C. elegans*, MutSγ promotes all crossovers [24]. However, other organisms, such as fission yeast and *Drosophila*, lack MutSγ. In *Drosophila*, an analogous function in protecting JMs from Sgs1/BLM anti-crossover activity has been inferred for two MCM-like proteins (mei-MCM). JM resolution in *Drosophila* occurs by the XPF-family endonuclease, MEI9-ERCC1 [25], [26]. In fission yeast, essentially all crossovers are generated by Mus81-Eme1, another XPF-family endonuclease [27]–[29]. In budding yeast, plants and mammals MutSγ-MutLγ is the predominant pathway of crossover formation, although Mus81-Eme1 (Mus81-Mms4 in budding yeast) also promotes a subset of crossovers [30]–[32]. Although Exo1-MutLγ, Mus81-Mms4, and Sgs1 are the major JM processing activities during budding yeast meiosis, at least two additional endonucleases can also facilitate resolution in budding yeast and metazoans. Yen1 can act as a backup resolvase in the absence of Mus81-Mms4 [13], [14], [33]. Similarly, Slx1–Slx4 is essential for resolution of a subset of JMs, specifically when Sgs1 is absent [13], [14], [34]–[36]. Collectively, the JM resolution and dissolution activities establish two essential conditions for efficient homolog disjunction at meiosis I: formation of crossovers to facilitate homolog biorientation and the efficient removal of all JMs that would otherwise impede chromosome separation.

Meiotic recombination is coordinated with global changes in chromosome morphology, including sister-chromatid cohesion and condensation. These processes are mediated by Structural Maintenance of Chromosome (SMC) complexes, large clamp or ring-like structures that include cohesin, condensin and Smc5/6. Whereas cohesin and condensin have wide-ranging effects on global chromosome morphology as well as DNA repair [37], the Smc5/6 complex appears to operate locally to attenuate recombination [38]–[43]. During mitotic growth, the Smc5/6 has been proposed to stabilize stalled replication forks and prevent recombination at the fork [43], [44]. However, if recombinational repair ensues, Smc5/6 also regulates late steps, promoting the resolution of recombination structures [38], [45]. The core Smc5/6 complex does not contain any DNA repair activities, raising the question of how it facilitates replication and recombination. One model posits that Smc5/6 regulates effector proteins via an intrinsic SUMO E3 ligase activity, catalysed by the associated non-SMC element Nse2/Mms21 [46]–[48]. This SUMO-mediated process has been inferred for regulation of telomeric and kinetochore proteins, and the establishment of cohesion around DSB sites (in mitotically cycling cells) [49]–[52]. However, this emerging paradigm has not been extended to enzymes involved in JM resolution. Genetic or physical interactions between Smc5/6 and JM resolving enzymes have not been established.

Based upon the findings that chromosome segregation appeared worse in *smc6* mutants that also lacked Sgs1 or Mus81, the Smc5/6 complex has been suggested to work in parallel with both Sgs1 and Mus81-Mms4 during mitotic DNA repair [40]. However, the severity of *smc5/6* mutants in combination with *mus81* or *sgs1* could equally reflect both separate as well as collaborative functions. The only physical interaction described to date is with the Mph1/FANCM DNA helicase, whose interaction with Smc5/6 does not depend upon sumoylation [53].

Despite the central role of Smc5/6 in orchestrating responses to DNA damage in mitotic cells, the role of Smc5/6 in meiotic recombination remains equivocal. In one study, a critical role for budding yeast Smc5/6 was inferred to occur during premeiotic S-phase, since abolition of meiotic DSBs by mutation of Spo11 did not improve the block to chromosome separation caused by *smc5/6* mutation [54]. In fission yeast, deletion of Nse5 or Nse6 is epistatic with the Mus81-Eme1 resolvase with regards to crossover generation suggesting that Smc5/6 regulates Mus81-dependent crossovers [55]. However, Mus81-Eme1 appears to be the sole resolvase acting during meiosis in fission yeast [56], [57], so it is unknown whether this paradigm extends to organisms that employ multiple resolvases; or whether Smc5/6 influences all resolution activities via global changes in chromosome structure. In contrast to fission yeast, in *C. elegans* animals depleted for Smc5/6, crossover formation appears normal but meiocytes contain excess RAD-51 foci indicative of unrepaired DSBs [58]. From these phenotypes, a specific defect in meiotic DSB-repair between sister-chromatids was inferred [58]. This raises the possibility that the Smc5/6 complex regulates a subset of recombination events and their resolution via specific resolvase activities.

A possible explanation for these apparently contradictory phenotypes is the extent to which different organisms employ the different JM resolution/dissolution activities [59]. In this study, we demonstrate that budding yeast Smc5/6 has essential roles during meiotic recombination in regulating the ordered formation of interhomolog joint molecules as well as their resolution. In *smc5/6* mutants, intersister dHJs as well as multichromatid joint molecules accumulate and fail to be resolved. For the latter, we show that Smc5/6 regulates Mus81-Mms4 activity in joint molecule resolution and localization to meiotic chromosomes. In contrast, the main resolvase activity during meiosis (MutLγ) appears to function independently of the Smc5/6 complex.

## Results

### Smc5/6 accumulates at centromeres, cohesion-binding sites, and double-strand breaks (DSBs)

Affinity-tagged Smc5-13myc and Nse4-TAP proteins were expressed throughout meiosis (Figure S1A). A subset of Smc5-13myc migrated as a highly molecular weight band that likely corresponds to the sumoylated species (Figure S1A). Smc5-13myc displayed linear or punctate immuno-staining patterns along meiotic chromosomes, during prophase I, that became undetectable at diplonema and metaphase I (Figure S1B). The punctate localization of Smc5-13myc was dependent upon Cdc6 (which is required for meiotic DNA replication) and to a lesser extent on the type-II topoisomerase Top2 (Figure S1). In contrast, chromosomal staining of Smc5/6 did not require Spo11 (required for DSB formation), Rec8 (cohesion), or the type-I topisomerases, Top1 and Top3 (Figure S1C and data not shown).

**Figure 1:**
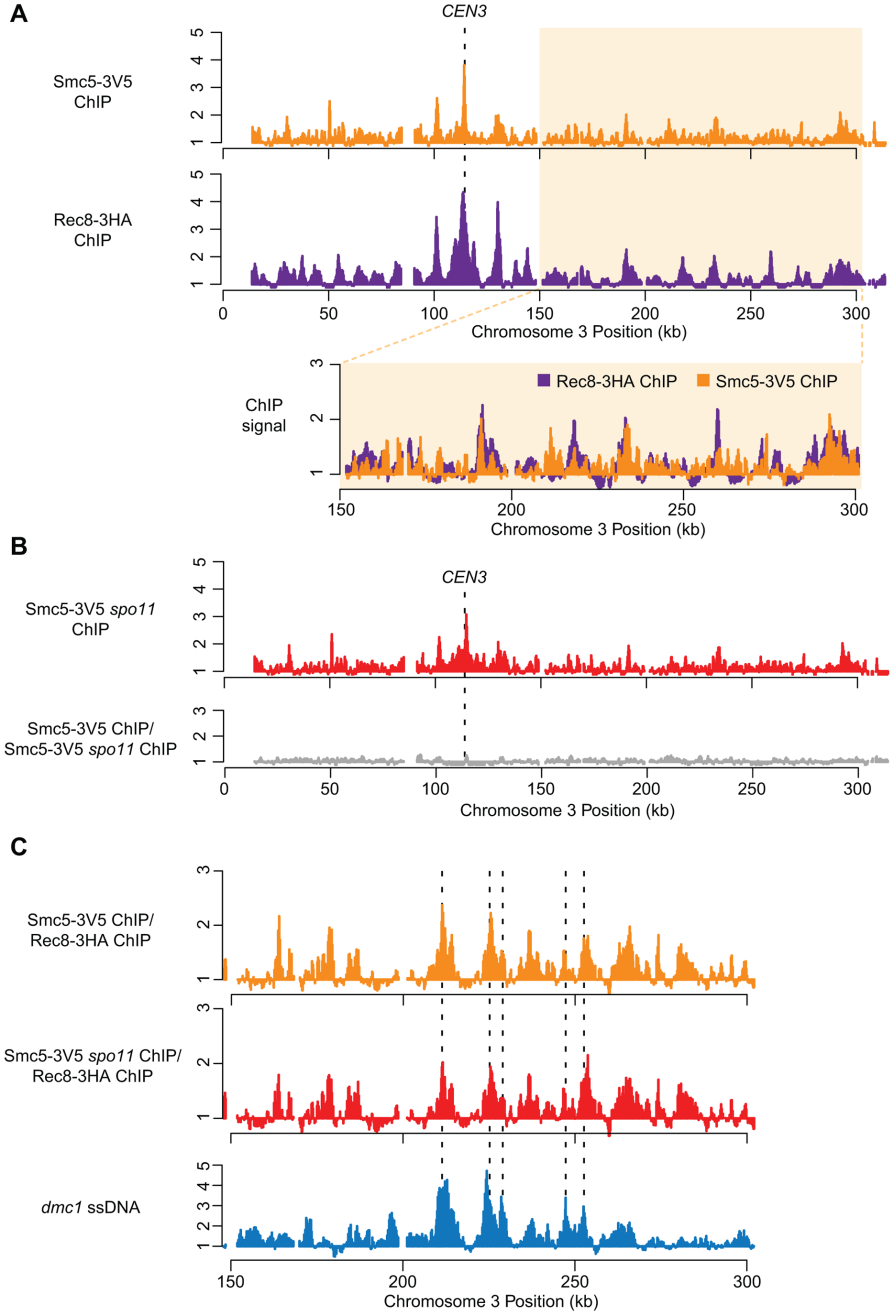
Smc5 associates with cohesin binding sites, centromeres, and DSBs. (A) DNA binding profiles for Smc5-3V5 (orange, H6671) and Rec8-3HA (purple, H4471, [65]) plotted for Chromosome III. Lower panel shows overlay of the right arm (150–300 kb) of Chromosome III. (B) DNA binding profiles for Smc5-3V5 in a *spo11Δ* strain (top panel, H6674) and the normalized DNA binding of Smc5-3V5 in *spo11Δ* strain versus Smc5-3V5 in the *SPO11* strain from (A) on Chromosome III. (C) The binding profile of Smc5-3V5 (orange) was normalized to Rec8-3HA binding using the data shown in (A) to reveal weaker, non-core binding regions. DSB sites mapped by ssDNA enrichment in the *dmc1*Δ mutant are indicated below (blue, H118, [100]). All ChIP experiments were carried out at 3 hours after transfer to SPM. Spindles reached their max. peak at 4 hours.

To obtain a higher resolution picture of Smc5/6 association with meiotic chromosomes, we carried out genome-wide ChIP-on-chip localization analysis for Smc5 tagged at its C-terminus with three V5 or 13 myc epitopes. Smc5 binds to many of the same chromosomal axis-associated sites as the meiosis-specific cohesin component, Rec8, and is similarly enriched at centromeres (Figure 1A, 1B). A similar, perhaps even more pronounced, enrichment at cohesin binding sites was also observed when a tagged Smc5-13myc protein was analyzed (Pearson's correlation coefficient (PCC) for Smc5-3V5 vs. Rec8 = 0.22, p<10^−15^; Smc5-13myc vs. Rec8 = 0.43, p<10^−15^; Figure S2). The enrichment of Smc5 at cohesin binding sites, centromeres, and telomeres is similar to the localization pattern previously described for Smc5/6 in vegetative cells [60]. However, in contrast to the mitotic distribution [60], neither we nor Xaver et al. [61] observed an increased density of Smc5/6 association sites along longer chromosomes during meiosis.

To determine whether the association of Smc5 with meiotic chromosomes depended upon DSB formation, we determined the binding profile in the absence of Spo11 (Figure 1B). Aside from a small overall reduction in binding, we observed no gross changes in the Smc5-3V5 distribution either at or between core sites in a *spo11Δ* strain (Figure 1B). This result is consistent with our observation that Smc5 immuno-staining on individual, spread meiotic nuclei is largely unaffected in the absence of Spo11 (Figure S1C), similar to that seen for Smc6 [54].

Some weaker binding sites also occurred in between the axis association sites defined by Rec8 (Figure 1A, lower panel). DSBs tend to occur in between the Rec8 axis association sites [62], [63], and Smc5/6 is recruited to DSBs in mitotic cells [60], [64]. Thus, we explored the idea that a fraction of Smc5/6 binds meiotic DSB sites. The locations of non-axis Smc5 association sites were determined by normalizing the Smc5 binding signal to the Rec8 signal (Figure 1C). This analysis revealed several additional binding sites along each chromosome (Figures 1C, S2). These weaker binding sites showed significant overlap with DSB sites (PCC = 0.28, p<10^−15^; Figure 1C), mapped by single-stranded DNA that accumu**l**ates at DSB sites in *dmc1Δ* mutants [65], [66]. Thus, Smc5/6 displays both a strong localization to chromosomal core sites and a weaker (perhaps more transient) localization to DSB sites. Several proteins involved in the formation and processing of meiotic DSBs localize to DSB hotspots even in the absence of DSB formation. Indeed, the Smc5 pattern, including DSB-correlated sites, is essentially unchanged in a *spo11* mutant (Figure 1C). This pattern is reminiscent of the binding profiles of Rec114 and other factors required for DSB formation, which are inferred to result from interaction of the DSB sites with the chromosome axes at the time of DSB formation [62], [63]. We conclude that Smc5/6 associates with cohesin association sites, centromeres, as well as DSB hotspots, and that this association occurs mostly independently of DSB formation.

The strong enrichment of Smc5/6 at centromeres (the strongest cohesin binding sites in the genome) as well as DSBs were also observed for the Smc6 subunit in independent experiments by Xaver *et al.* (2013). Using ChIP-seq, they observed a small enrichment at cohesin association sites as well. The differences in the magnitude with which Smc5 (our study) or Smc6 (Xaver et al.) binds cohesin associated sites is likely due to the affinity tags being placed on different subunits of the complex. These may be differentially accessible to the antibodies and/or local DNA. It is unlikely that the enrichment of Smc5/6 that we observe in the ChIP experiments is non-specific, because the patterns are similar for both Smc5-3V5 and Smc5-13myc, which were immunoprecipitated with different antibodies and resins. Moreover, other DSB factors tagged with 13myc did not show any significant enrichment to cohesion binding sites by ChIP-chip (data not shown). Finally, consistent with a fraction of Smc5/6 binding to chromosomal axes, more than 50% of Smc5-13myc foci localize to the synaptonemal complex (central element component, Zip1) in our experiments (Figure S1B). This makes it highly unlikely that non-specific association of the antibodies with proteins or sequences at cohesin binding sites gives rise to false peaks.

### Smc5/6 is required for chromosome separation following meiotic DSB formation

**Figure 2:**
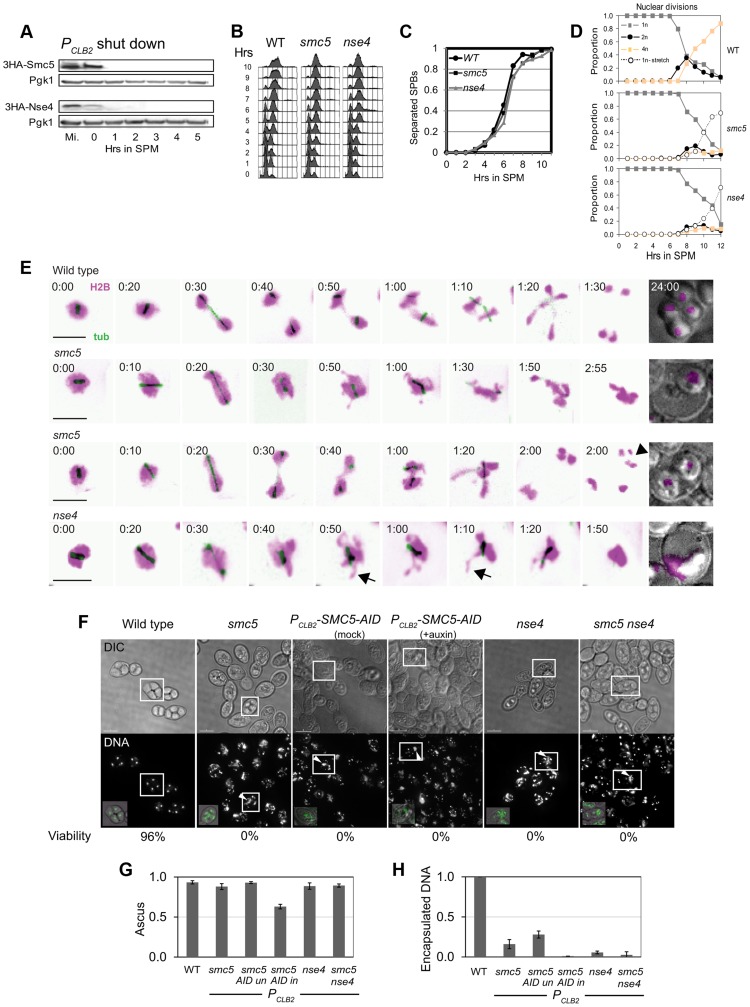
Meiotic depletion of Smc5 or Nse4 leads to meiotic catastrophe. (A) Western blot of depletion of 3HA-Smc5 (Y941) and 3HA-Nse4 (Y942) protein levels under the *P_CLB2_* promoter. Mutants are referred to as *smc5* and *nse4* throughout. (B) FACS analysis of S-phase progression in wild type (Y940), *smc5* (Y941) and *nse4* (Y942) mutants. (C) Population kinetics of spindle pole body separation (n = 200 *per* time point). (D) Population kinetics of nuclear divisions (n = 200 *per* time point). (E) Montage of time series of nuclear divisions and spindle dynamics from representative time-lapse movies. H2B-mCherry and Tub1-GFP are pseudo-coloured in magenta and green, respectively. Maximum projections are shown. Bars: 4 µm. Full movies are available as Supplemental Movies S1 to S4. Arrows indicate examples of nuclear spikes and arrowheads show fragmentation/micronuclei. Strains: WT (Y3606), *smc5* (Y3627), *nse4* (Y3630). (F) DNA encapsulation failure in *smc5* and *nse4* mutants. Upper panel DIC, lower panel, DAPI (DNA). The boxed asci are shown with DNA (green) overlaid in the insets in the lower panel, bottom left. Note that the samples are taken from different time points in the various strains. Bars, 5 µm. (G) Proportion of cells completing meiosis and forming an ascus (di-tyrosine fluorescence). (H) Proportion of asci with encapsulated DNA (bottom). All data were collected after 24 hours in liquid sporulation medium. Three independent diploids were assessed for each genotype (standard deviations are shown). Strains: WT (Y1381), *smc5* (Y2705), *P_CLB2_-SMC5-AID* (Y3252), *nse4* (Y2704), *smc5 nse4* (Y3185).

Smc5 localization at sites of meiotic DSBs, cohesin binding, and centromeres suggests possible roles for the Smc5/6 complex in meiotic recombination and chromosome morphogenesis. Since Smc5/6 is essential, its meiotic functions were studied by depleting the core component, Smc5, and the kleisin (Nse4) using the *CLB2* promoter, which is strongly repressed in meiosis [67] (Figure 2A). Meiosis-specific depletion circumvents the need for temperature-sensitive conditional alleles that require temperature-shift protocols, which may be complicated by the fact that several chromosomal processes are affected by temperature [20], [68].

Strains carrying the *P_CLB2_-SMC5* or *P_CLB2_-NSE4* alleles (hereafter, *smc5* and *nse4*) had normal vegetative growth and were not sensitive to DNA damaging agents (data not shown). In meiosis, although bulk DNA replication and spindle pole body separation were essentially normal (Figure 2B,C), nuclear divisions were severely defective (Figure 2D). Time-lapse studies revealed that although nuclear divisions were attempted at both anaphase I and II, as soon as spindles disassembled, DNA bodies retracted into a single mass that subsequently failed to be encapsulated in the spores (Figure 2F,H; Movie S1, S2, S3). None of 30 randomly-selected cells imaged for either the *smc5* or *nse4* mutant managed to stably separate their DNA at the completion of meiosis I or II (Figure 2E). Micronuclei or fragmented nuclei as well as aberrant chromosomal morphologies were also observed (Figure 2E, arrows). Despite the severe nuclear separation defect, both the *smc5* and *nse4* mutants went on to complete meiosis and form asci with similar efficiencies to wild type (Figure 2F,G, ∼90%). However, the failure to separate the DNA at meiosis I and II, prevented encapsulation of DNA into the spores (Figure 2H). This “meiotic catastrophe” was more pronounced for the *nse4* mutant compared to the *smc5*. This is likely due to more efficient depletion of Nse4, because when Smc5 was further depleted using an auxin-inducible degron fusion (*P_CLB2_-SMC5-AID*, [69]), the nuclear separation defect became more severe and analogous to that seen in *nse4* cells (Figure 2F–H). We could not determine unequivocally that the *P_CLB2_-SMC5-AID* was more depleted than *P_CLB2_-SMC5*, since the depletion by *P_CLB2_*-*SMC5* alone rendered Smc5 undetectable by Western blot (Figure 2A, data not shown). However, analysis of *SMC5-AID* (without *CLB2* depletion) demonstrated that auxin-induced degradation of Smc5 does occur, even when Smc5 is expressed at normal levels from its native promoter (Figure S3). Together, these experiments support the notion that the less severe meiotic catastrophe seen in the *P_CLB2_*-*SMC5* cells relative to *P_CLB2_*-*NSE4* is due to less efficient depletion of Smc5. However, they do not rule out the possibility that Nse4 has a function distinct from Smc5, perhaps acting as part of the Nse1-Nse3-Nse4 subcomplex [70].

**Figure 3:**
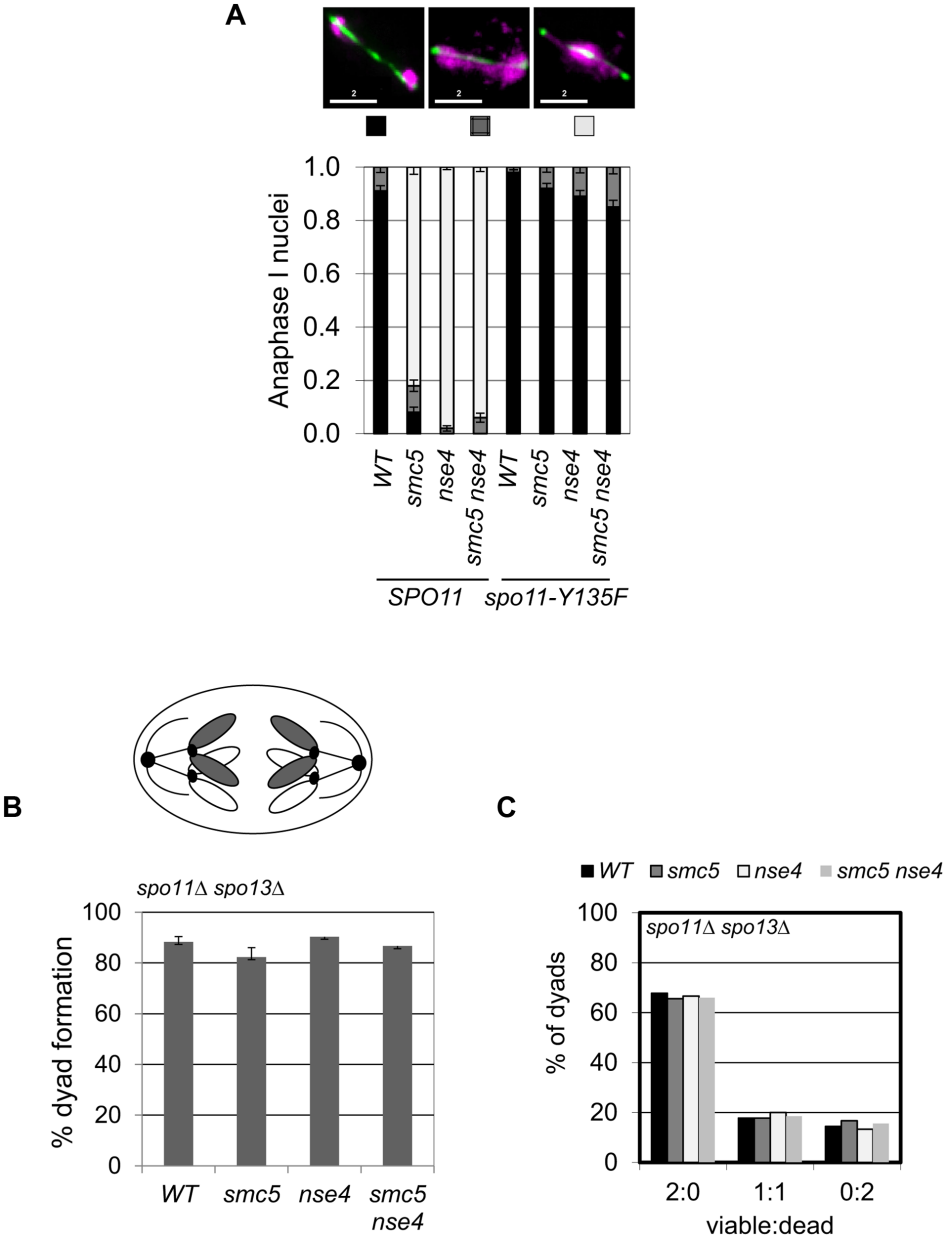
Meiotic depletion of Smc5 and Nse4 leads to Spo11-dependent nuclear separation defects in meiosis. (A) Catalytic-dead Spo11 mutation rescues nuclear separation at anaphase I in the Smc5/6 mutants (n≥100). Bars indicate standard error bars for a proportion. Strains: WT (Y1381), *smc5* (Y2705), *nse4* (Y2704), *smc5 nse4* (Y3185), *spo11-Y135F* (Y3147), *spo11-Y135F smc5* (Y3150), *spo11-Y135F nse4* (Y3153), *spo11-Y135F smc5 nse4* (Y4202). (B and C) Schematic of sister chromatid segregation at meiosis I in *spo11*Δ *spo13*Δ mutants. Dyad formation and viability after 24 hours in sporulation medium of Smc5/6 mutants in conjunction with the *spo11*Δ *spo13*Δ bypass. Strains: *spo11*Δ *spo13*Δ (Y2816), *spo11*Δ *spo13*Δ *smc5* (Y2846), and *spo11*Δ *spo13*Δ *nse4* (Y2848).

To determine whether meiotic catastrophe required the initiation of recombination, we abolished the DSB activity of Spo11, using the catalytically-dead *spo11*-*Y135F* allele. This suppressed the nuclear separation defects of both *smc5* and *nse4* (Figure 3A). To address whether DNA damage or replication intermediates accumulated during pre-meiotic S-phase contribute to the nuclear separation defects of *smc5* and *nse4*, we converted meiosis I into a single mitosis-like division by de-protecting centromeric cohesin at anaphase I (*spo13*Δ), while simultaneously inactivating recombination (*spo11*Δ). No effect of *smc5* or *nse4* mutation on either dyad formation or spore viability was observed (Figure 3B,C). This experiment rules out the possibility that gross S-phase defects alone are responsible for the meiotic chromosome segregation failure in *smc5* and *nse4* mutants. Thus, depletion of Smc5/6 causes severe recombination-dependent meiotic catastrophe. This is in sharp contrast to the *smc6–9* temperature sensitive allele, which was previously shown to cause meiotic catastrophe independently of Spo11 [54].

### Joint molecule metabolism is severely defective in *smc5/6* mutants

**Figure 4:**
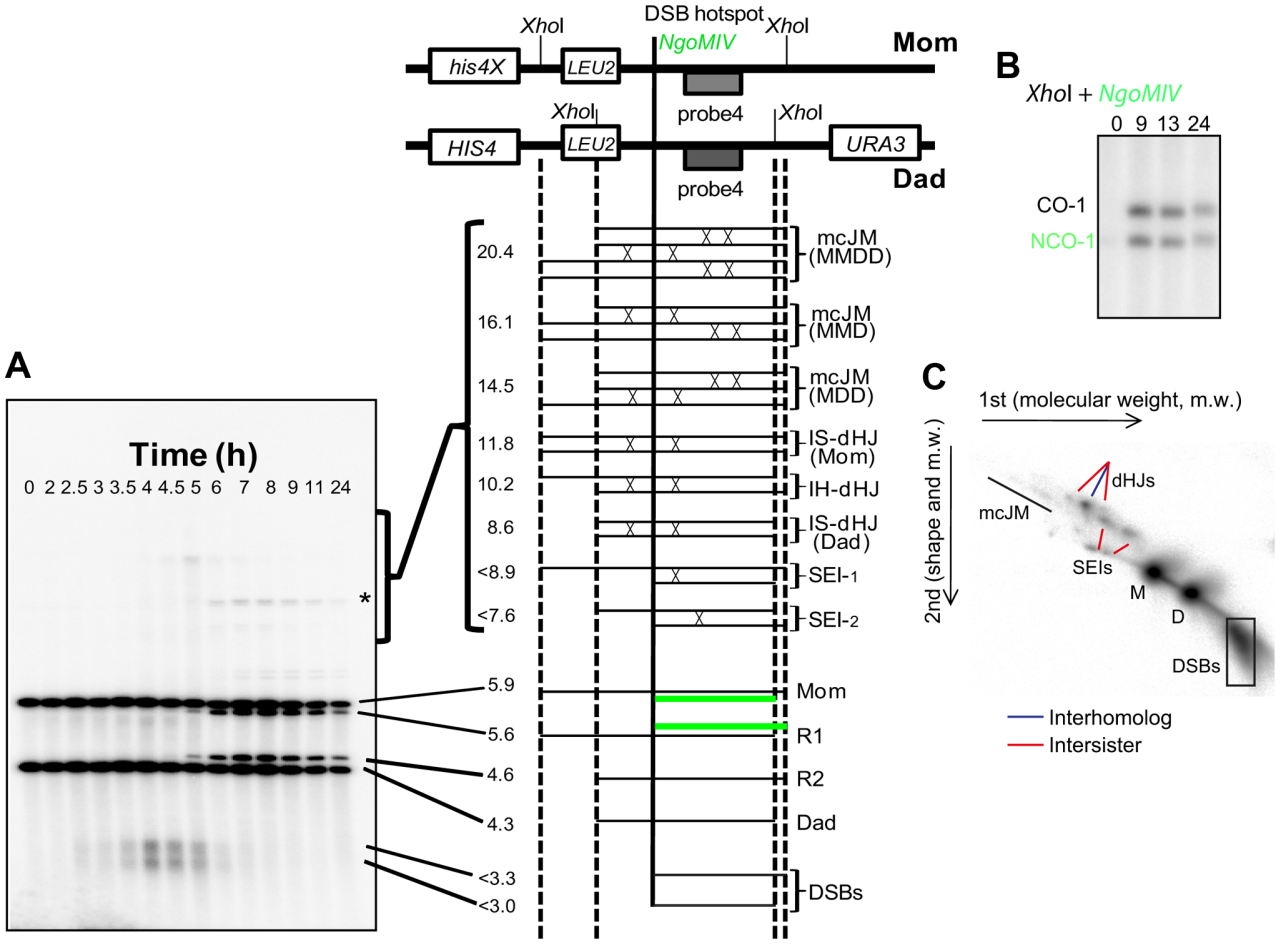
Assessment of meiotic recombination at the *HIS4LEU2* hotspot. (A–C) The *HIS4LEU2* hotspot. mcJM: multichromatid joint molecules (abbreviations: M-Mom, D-Dad), IS-dHJ intersister double Holliday Junctions, IH-dHJ interhomolog double Holliday Junctions, SEI- single-end invasions, DSBs- double strand breaks. Digesting with *Xho*I gives diagnostic band sizes from parental molecules, Mom and Dad, as well as recombinant fragment lengths (R1 and R2). These are predominantly crossovers. The different molecules can be separated on 1D (A) and shape-dependent separation on 2D gels (C). Further digestion with *NgoMIV* differentiates noncrossovers from parental molecules (B). The * indicates a non-specific signal.

To investigate possible roles of Smc5/6 in meiotic DSB repair, we analysed meiotic recombination at the well-characterized *HIS4LEU2* recombination hotspot construct using a series of Southern blot assays [71], [72] (Figure 4). Restriction site polymorphisms combined with 1D or 2D gel electrophoresis and Southern analysis allow formation of DSBs, crossovers, noncrossovers and several different species of joint molecules to be monitored at *HIS4LEU2*. Joint molecules include single-end invasions, double Holliday Junctions (formed between homologs or between sister chromatids) and multichromatid joint molecules (involving 3 or 4 chromatids) [10], [71], [72].

**Figure 5:**
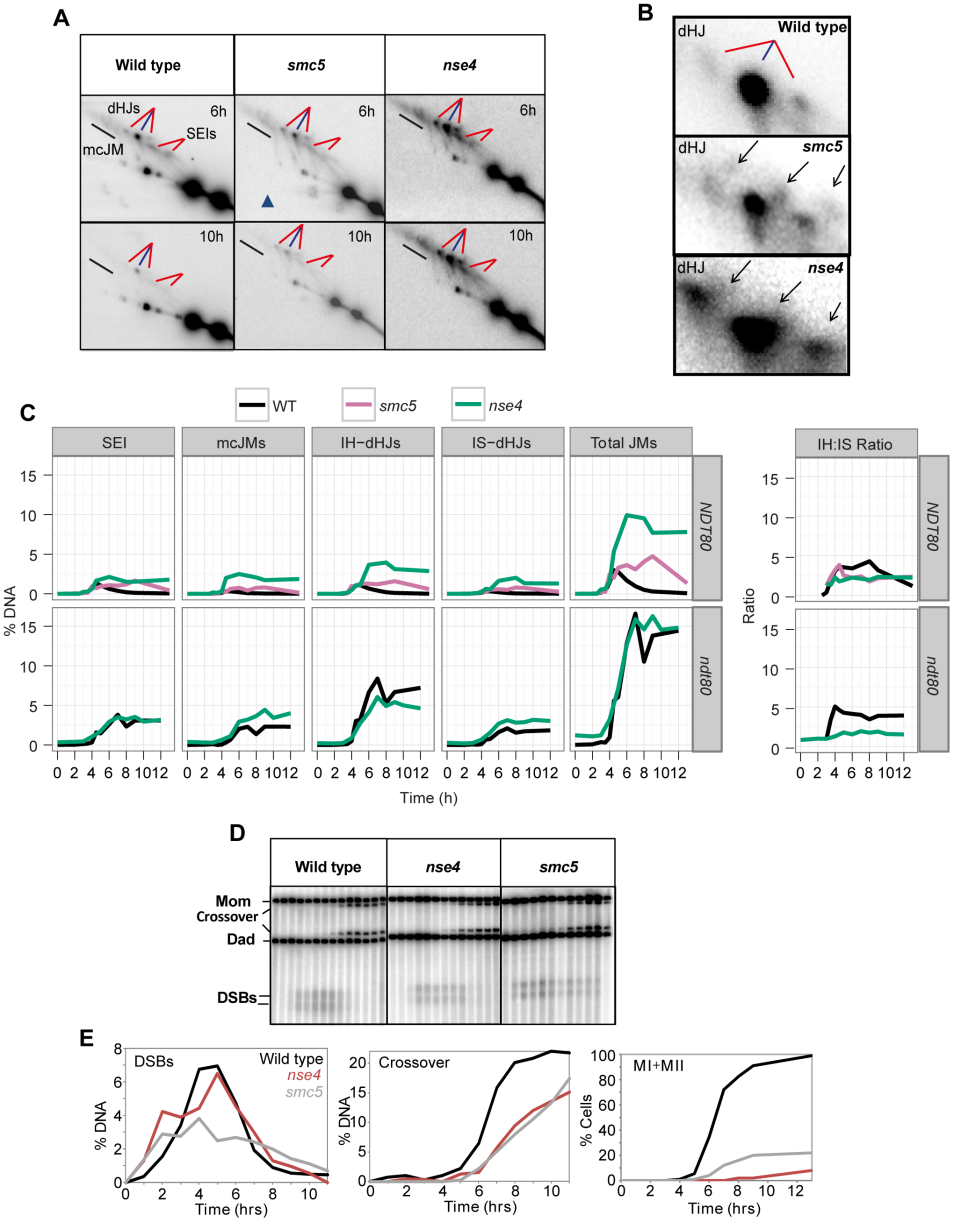
Aberrant joint molecules accumulate in *smc5* and *nse4* mutants. (A) Examples of time courses from 2D gels. Blue lines point at joint molecules formed between homologous chromosomes (interhomolog, IH) and red lines indicate joint molecules composed of sister chromatids (intersister, IS). Strains: WT (Y2976), *smc5* (Y1211), *nse4* (Y1212). (B) Enlarged dHJ spots from wild type, *smc5*, and *nse4*. (C) Smoothed levels of single end invasions (SEIs), multichromatid joint molecules (mcJMs), interhomolog-double Holliday Junctions (IH-dHJs), intersister-double Holliday Junctions (IS-dHJs), IH-dHJ to IS-dHJ (IH∶IS) ratio, and total joint molecules (Total JMs). Cumulative levels of recombination were assessed in the *ndt80*Δ background (lower panel). Strains: *ndt80* (Y3025), *ndt80 nse4* (Y3843). (D) Examples of time course analyses of double-strand break and crossover formation. (E) Quantification of DSB, crossover, MI+MII nuclear divisions.

In wild-type cells, joint molecule levels peaked around 4.5 hours, at ∼3% of hybridizing DNA, and disappeared by 8 hrs, when the majority of cells had completed the meiotic divisions (Figure 5A, C). In contrast, joint molecules in the *smc5* mutant appeared with normal timing but persisted at high levels (4.7%) until at least 9 hrs. The *nse4* mutant had a much more severe defect in joint molecule resolution, with very high levels of joint molecules (10%) persisting at 13 hrs (Figure 5C), when wild type cells have completed the meiotic divisions (Figure 2D). The level of unresolved joint molecules detected in the *nse4* mutant is at least 3-fold higher than any other single mutant analyzed to date and is reminiscent of mutants that simultaneously lack multiple joint molecule resolution or dissolution activities [13], [14], [33].

Closer inspection of both the intersister- and interhomolog-dHJ signals revealed additional spots or smears (Figure 5B). In the 1^st^ dimension, these new signals migrated ahead of the main dHJ spots, suggesting a lower molecular weight. In contrast, the signals were retarded in the 2^nd^ dimension relative to the main dHJ spots. It is currently unclear whether these JM species are extreme variants of dHJs (e.g. with very widely spaced Holliday junctions) or aberrant structures that are never formed in wild type. Regardless, their existence indicates that JM formation as well as resolution is altered in *smc5* and *nse4* mutants.

In contrast to joint molecules, the appearance, disappearance, and resection of DSBs in *smc5* and *nse4* mutants occurred with largely wild-type kinetics (Figure 5D, Figure S4, S5). These observations suggest that the initiation of recombination occurs without any significant defects and that *smc5*/6-depleted cells are specifically defective in steps leading to the formation and resolution of joint molecules.

Crossover formation was delayed and final levels were reduced by 20–30% in *smc5* and *nse4* mutants. Crossovers accumulated to 22% of the DNA signal in wild type, while *nse4* and *smc5* mutants formed, respectively, 15% and 17% (Figure 5D and E, S4B). The double mutant (*smc5 nse4*) was indistinguishable from the *nse4* single mutant (Figure S4).

### *smc5* and *nse4* mutants accumulate joint molecules between homologs, sister chromatids, and multiple chromatids

To understand whether *smc5/6* mutants accumulate a specific class of joint molecules, we separately quantified the levels of single-end invasions (SEIs), double Holliday Junctions (dHJs), and multi-chromatid joint molecules (mcJMs) using 2D gels (Figure 5C). Compared to the wild type, the *smc5* mutant showed slightly elevated levels of all joint molecule species and delayed disappearance. In the *nse4* mutant, all classes of joint molecule accumulated to higher levels than wild type and remained elevated throughout the meiotic time course (Figure 5C). We infer that Smc5/6 plays a general role in joint molecule metabolism.

### Homolog bias is decreased in the *smc5* and *nse4* mutants

Our observations that *smc5* and *nse4* mutants accumulate unresolved joint molecules while still forming high levels of crossovers raise the possibility that more total joint molecules are made in these mutants. To address this question, we used the resolution-defective *ndt80*Δ mutant to quantify joint molecule formation independently of changes in the efficiency of resolution [6], [8]. In both *ndt80*Δ and *ndt80*Δ *nse4*, total accumulated joint molecules plateaued at similar levels and with essentially identical kinetics (∼15%, Figure 5C, lower panel *ndt80*). However, intersister dHJs and multichromatid JMs were increased at the expense of interhomolog dHJs when compared to the *ndt80*Δ mutant alone (Figure 5C; lower panel *ndt80*). Consistently, the ratio of interhomolog dHJs to intersister dHJs (“interhomolog bias”) was decreased from 4∶1 (4.1±0.5) in the *ndt80*Δ strain, to 2∶1 in both mutants (1.9±0.3 and 1.7±0.2 in *smc5 ndt80*Δ and *nse4 ndt80*Δ, respectively; Figure 5C and data shown not). Similarly, when *NDT80* was present, the IH∶IS dHJs ratio was also decreased from a steady-state ratio of ∼3.5±0.4 in wild type to 2.1±0.2 in *smc5* and 2.1±0.2 in *nse4* (P<0.01; Figure 5C). We conclude that overall JM levels are not significantly altered by depletion of Smc5/6, but the spectrum of JMs is altered such that intersister and multichromatid joint molecules are increased at the expense of interhomolog dHJs. Similar conclusions have been reached by two other labs [61], [73].

### Combined depletion of *sgs1* and *smc5/nse4* synergistically increases joint molecule accumulation

**Figure 6:**
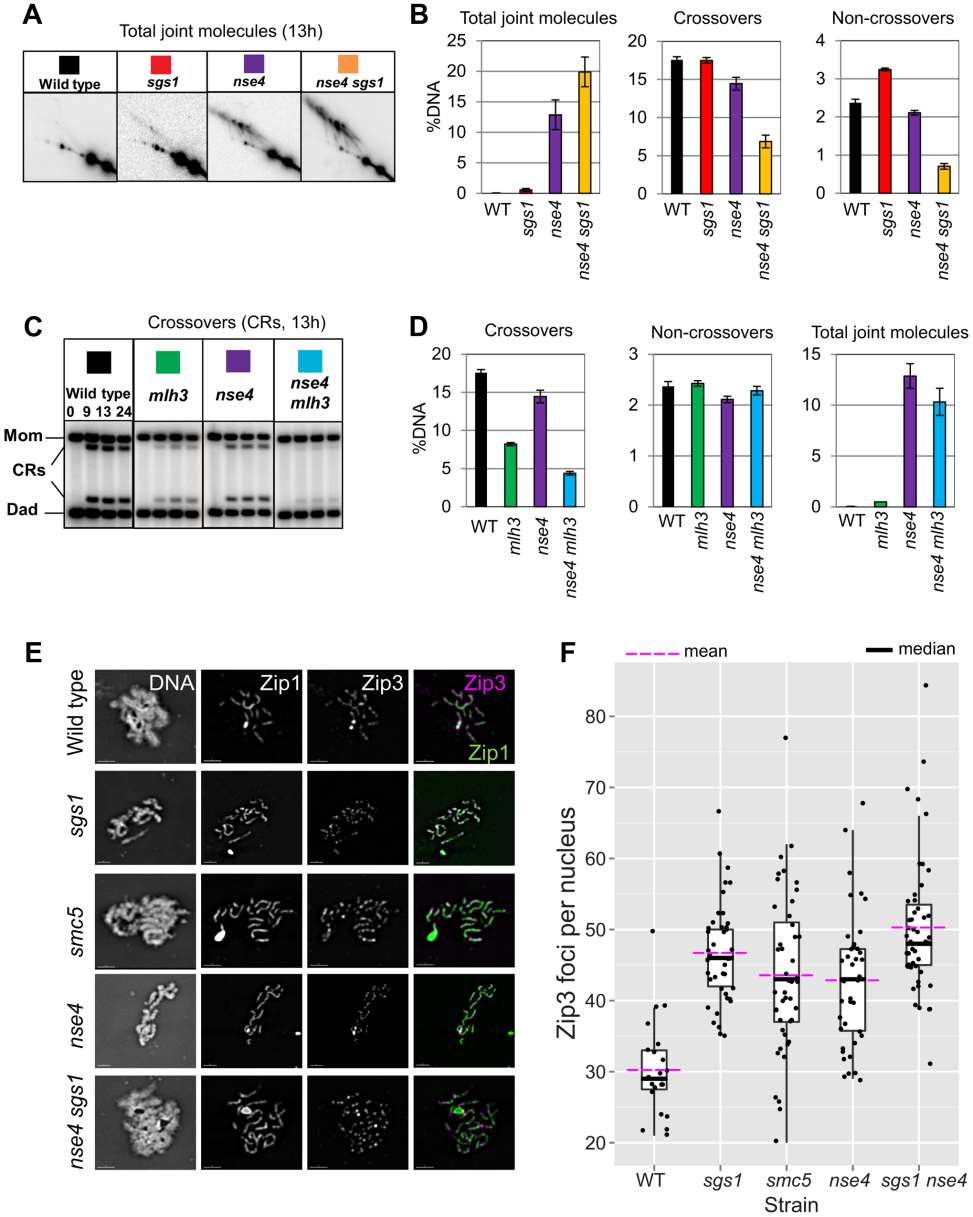
Sgs1 and MutLγ are functional in *smc5/6*. (A) Representative images of 2D analysis from *sgs1* mutant (*P_CLB2_-3HA-SGS1*) in combination with *nse4*. (B) Quantification of total joint molecules, crossovers and non-crossovers, and total joint molecule levels at meiotic endpoints (13 hours). Quantification from three independent diploids; error bars represent the standard deviation. (C) Representative images of crossover formation in *mlh3*Δ mutants, in combination with *nse4*. (D) Quantification of crossovers, noncrossovers, and total joint molecules levels from three independent diploids (13 hours). (E,F) Analysis of Zip3 foci. Representative images and Tukey-Kramer box-and-whisker plot of 30 nuclei from each strain (boxes represent the 25^th^–75^th^ percentile; the median value is denoted by the horizontal bar, and the whiskers are 1.5× the 25–75^th^ percentile or max or min. values- whichever are the lowest). Fold increase in Zip3-GFP foci relative to wild type was calculated based on the arithmetic mean (horizontal bar, magenta). Note that the Zip3-GFP causes some polycomplex formation of Zip1 predominantly in the mutants but also in the wild type. The distributions of all four mutant strains were significantly different from wild type (p<0.01, Kruskall-Wallace). Strains: WT (Y1435), *sgs1* (Y3591), *smc5* (Y3514), *nse4* (Y3511), *sgs1 nse4* (Y3636).

In budding yeast meiosis, Sgs1 helicase is a central regulator of meiotic recombination intermediates during meiotic prophase [10]–[14]. Similar to *smc5* and *nse4* strains, *sgs1* mutants form more multichromatid and intersister JMs, but fewer interhomolog dHJs [10]. However, unlike *smc5* and *nse4*, joint molecule resolution and chromosome segregation occur efficiently in *sgs1* cells. To examine the relationship between Smc5/6 and Sgs1, we combined *smc5* or *nse4* depletion mutants with meiosis-specific depletion of Sgs1 (*P_CLB2_-3HA-SGS1*, hereafter *sgs1*). Both crossover and noncrossover formation were synergistically decreased in the *smc5 sgs1* and *nse4 sgs1* double mutants (Figure 6A and data not shown). On their own, *smc5*, *nse4*, and *sgs1* single mutants exhibited, respectively, 1.5%, 13%, and 0.6% joint molecules at time points when cells had completed meiosis (13 h; Figure 6A and data not shown). In both the *smc5 sgs1* and *nse4 sgs1* double mutants, we observed synergistic increases in all species of joint molecules, which accumulated to 14% and 20%, respectively (Figure 6A and data not shown). This level of accumulation of joint molecules is similar to that seen when both Sgs1 helicase and structure-specific endonucleases (Mus81-Mms, Slx1–Slx4, and Yen1) are lacking (∼20%, [13], [14]). Given that crossover and noncrossover levels are high in the *smc5* and *nse4* strains (Figure 5E, 6B), we infer that Sgs1 can still function proficiently to promote crossovers and noncrossovers when Smc5/6 is depleted.

### Absence of MutLγ diminishes crossing over in the *nse4* mutant

MutLγ is inferred to be an endonuclease that specifically promotes the resolution of dHJs into crossovers along the MutSγ pathway for crossing over [17], [22], [23], [74]. To test whether the crossovers formed in *smc5/6* mutants are formed via this pathway, we deleted *MLH3* in the *smc5* and *nse4* mutants. Although the *mlh3*Δ mutation alone caused a substantial decrease in crossovers (compare 18%±0.5% in wild type to 8.2%±0.2% in the *mlh3*Δ; Figure 6D), crossing-over in the double mutants was further decreased (4.5±0.5% for *smc5 mlh3*Δ and 4.4%±0.2% for *nse4 mlh3*Δ; Figure 6D; data not shown for *smc5*). Importantly, noncrossovers were unaffected, consistent with the notion that MutLγ predominantly yields crossovers [23], [75]. We infer that MutLγ is active and responsible for most crossovers in *smc5/6* mutants.

### Zip3 foci are increased in *smc5* and *nse4* mutants and synapsis occurs with wild-type kinetics

MutLγ promotes crossovers in conjunction with MutSγ, which in turn interacts with and requires Zip3, for its association with meiotic chromosomes (reviewed in [76]). Zip3 associates in a punctate pattern with meiotic chromosomes at axial association sites, where homolog synapsis initiates and where crossovers will form [2], [77]. We reasoned that if MutLγ and MutSγ are active in the *smc5* and *nse4* mutants, then Zip3 localization along meiotic chromosomes as well as synapsis should occur with normal proficiency. To assess whether this was the case, we detected a GFP-tagged Zip3 and co-stained for the synaptonemal complex protein, Zip1 (Figure 6E). In the wild type, we observed ∼30 Zip3-GFP foci in pachytene nuclei; this number was increased 1.2–1.3-fold in the *smc5* and *nse4* mutants (Figure 6F). This increase was similar in magnitude to that observed in an Sgs1-depleted strain (Figure 6F) [78].

Zip3 promotes the assembly of the synaptonemal complexes (SC). No significant differences were observed in the kinetics of SC assembly and disassembly, including turnover of Zip1 protein, in the *smc5* and *nse4* when compared to the wild type (Figure S6). Thus, early steps in MutSγ-dependent crossover formation and initiation of synapsis are not adversely affected by depletion of Smc5/6.

### Smc5/6 affects Mus81-Mms4-dependent joint molecule resolution

Our results further distinguish phenotypes observed for Smc5/6 from those of Sgs1: Smc5/6 depletion does not suppress the crossover defect of MutLγ, unlike that seen in *sgs1 mlh3*Δ mutants [10]. These phenotypes could be explained if Smc5/6 has additional roles in joint molecule resolution via the Mus81-Mms4 endonuclease, which becomes essential for resolution in *sgs1* mutants [11], [12].

To determine whether Smc5/6 affects the functions of structure-selective endonucleases during meiosis, we deleted *MMS4* (*mms4*), the regulatory subunit of Mus81, and also the two cryptic endonucleases Yen1 and Slx1–Slx4 [79]. Yen1 and Slxl–Slx4 have only minor, if any, roles in joint molecule resolution in otherwise wild-type cells [13], [14], [33].

**Figure 7:**
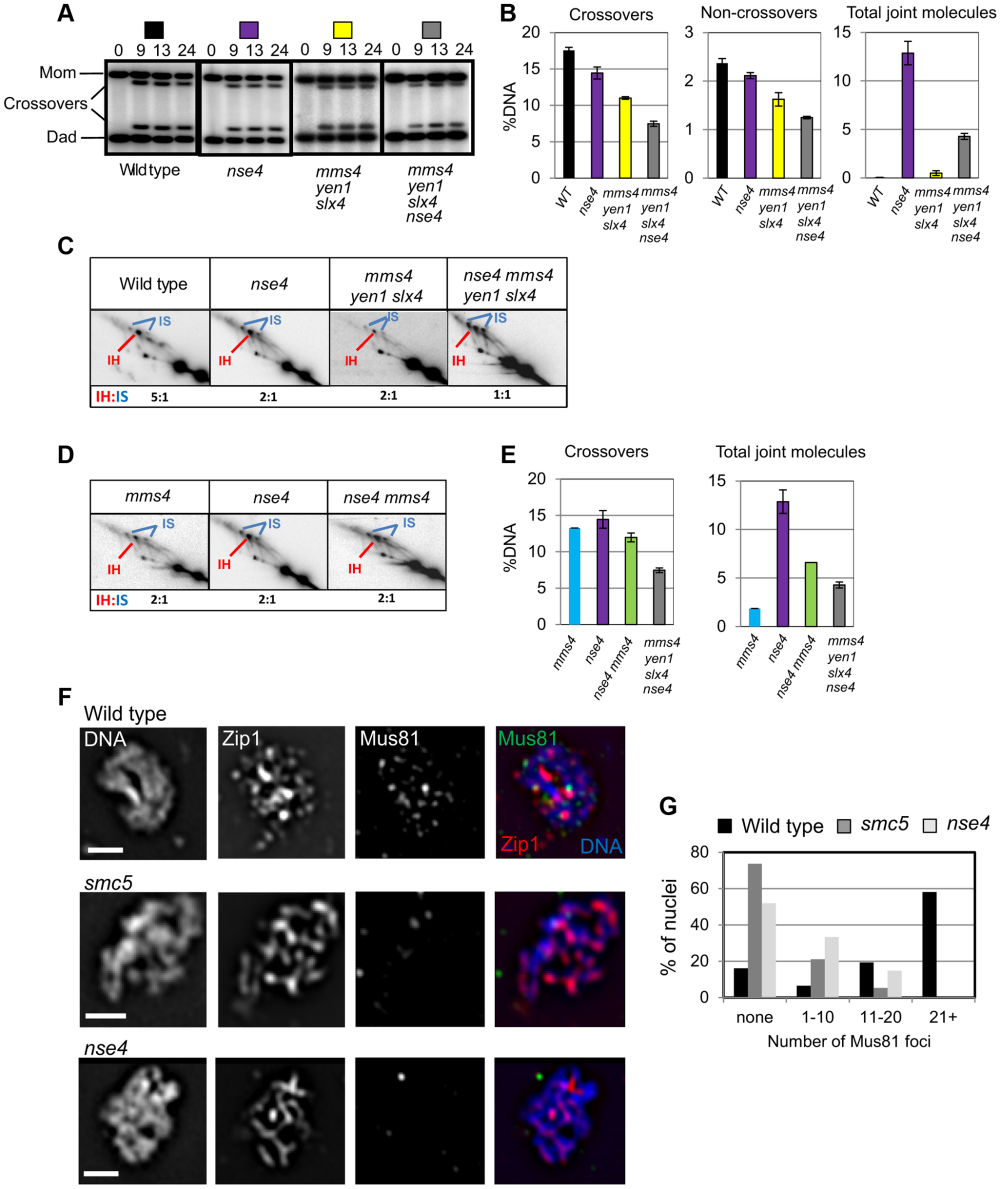
Smc5/6 regulates joint molecule resolution by Mus81-Mms4. (A) Representative images of 1D analysis of crossover levels. (B) Quantification of crossovers, non-crossovers, and total joint molecule levels at meiotic endpoints (13 hours). Quantification from three independent diploids; error bars represent the standard deviation. (C) Representative images of 2D analysis of IH∶IS ratio in *nse4* and *nse4 mms4 yen1 slx4* quadruple mutants in the *ndt80*Δ background (13 hours). Data from three independent diploids. (D) Representative images of 2D analysis of IH∶IS ratio in *nse4* and *nse4 mms4* mutants the *ndt80*Δ background (13 hours). Data from three independent diploids. (E) Quantification of crossovers and total joint molecules in *nse4 mms4* mutants compared to individual single mutants and the *nse4 mms4 yen1 slx4* quadruple mutant. (F,G) Representative images of Mus81 foci on spread, meiotic nuclei and quantification of Mus81-9myc foci. Nuclei were selected on the basis of linear Zip1 structures (pachynema). 100 nuclei were assessed for each strain. For the *mms4* single strain, we ran only one diploid in parallel with the *nse4* mutants. These data were similar to those described previously [14]. Strains: WT (Y3137), *smc5* (Y3135), and *nse4* (Y3144).

Crossover levels were roughly similar in the *mms4 yen1 slx4* mutant (11±0.4%), *smc5* (12%±0.7%) and *nse4* (14.5±1.7%) mutants (Figure 7A,B and data not shown). The *nse4 mms4 yen1 slx4* quadruple mutant had a further reduction in the levels of crossovers (7.4±0.7%; Figure 7A,B). Noncrossovers were also further decreased in the *nse4 mms4 yen1 slx4* quadruple mutant. In the wild type, the noncrossover signal contributed 2.4±0.1%, compared to 2.1±0.1% in the *nse4* mutant, 1.6±0.3% in the *mms4 yen1 slx4* mutant and 1.3±0.1% in *nse4 mms4 yen1 slx4* quadruple mutant (Figure 7A,B).

At least two reasons could account for the further loss of crossover and noncrossover products in the *nse4 mms4 yen1 slx4* quadruple mutant. Smc5/6 could promote joint molecule resolution in parallel with one or more of the three endonucleases. Alternatively, the formation of joint molecules leading to crossovers and noncrossovers could be perturbed. Analysis of joint molecules in the *nse4 mms4 yen1 slx4* cells lends support to the latter possibility (Figure 7C). The *nse4 mms4 yen1 slx4* mutant displayed a further decrease in the IH∶IS dHJ ratio (1∶1) compared to the *nse4* single and *mms4 yen1 slx4* triple mutants (2∶1). This indicates that Smc5/6 operates in parallel with the resolvases to promote interhomolog template bias (Figure 7C). Assuming a direct relationship between interhomolog-dHJs and the generation of interhomolog products (crossover and noncrossover), the decreased IH∶IS bias (50%) in the *nse4 mms4 yen1 slx4* mutant would be predicted to lead to a loss of half the crossovers (predicted 7.3% crossover products based on the 14.5% crossovers seen in the *nse4* mutant). The observed value of 7.4% crossovers (Figure 7B) is in good agreement with this. The additive reduction of interhomolog bias in the *nse4* and *mms4 yen1 slx4* mutants is therefore sufficient to explain the further decreases in crossover and noncrossover levels seen in the *nse4 mms4 yen1 slx4* quadruple mutant.

To further address which endonuclease was affected by Smc5/6, we focussed upon analysing the genetic interaction with Mus81-Mms4 (Figure 7D, E). Crossover levels (Figure 7E) as well as the IH∶IS dHJ ratios (Figure 7D) were similar in the *mms4*, *nse4*, and *nse4 mms4* mutants. These observations show that abolishing Mus81-Mms4 activity has little consequence for joint molecule resolution at least when Smc5/6 is depleted. Moreover, crossover levels were substantially higher in the *nse4 mms4* mutant compared to the *nse4 mms4 yen1 slx4* quadruple mutant, which suggests that Yen1, or more likely, Slx1–Slx4 promotes a significant amount of crossing over, presumably via a function that promotes interhomolog bias (Figure 7C).

In contrast to the effect of depleting Sgs1 in the *nse4* mutant background, the level of unresolved joint molecules did not increase in the *nse4 mms4 yen1 slx4* quadruple mutant, but instead decreased (compare 4.3±0.6% to 12.9±2.4% in the *nse4* single mutant; Figure 7B). This was also the case for the *nse4 mms4* mutant (6.6% unresolved joint molecules; Figure 7E). We interpret there results to mean that when Smc5/6 is depleted, the Mus81-Mms4 endonuclease renders a significant proportion of joint molecules non-cleavable by Sgs1 and/or MutLγ.

### Association of Mus81 with meiotic chromosomes is defective in *smc5* and *nse4* mutants

To investigate whether chromosomal localization of Mus81-Mms4 was affected in the *smc5* and *nse4* mutants, we assessed the ability of Mus81-9myc to form foci on spread, meiotic chromosomes at pachytene, when joint molecules reach their highest levels. Pachytene-stage nuclei were selected by virtue of linear staining of the synaptonemal complex component, Zip1, and the numbers of Mus81 foci were counted. In the wild type, the majority of pachytene nuclei contained more than 20 distinct foci of Mus81. In contrast, the majority of nuclei from the *smc5* and *nse4* mutants had no distinct Mus81 foci (Figure 7F,G). We ruled out that this was due to reduced levels of Mus81-Mms4 protein or failure to hyperactivate Mus81-Mms4 upon exit from pachytene (Figure S7). These observations imply that the ability of Mus81 to associate with or be stabilized on meiotic chromosomes is diminished when Smc5/6 complexes are depleted.

### Smc5/6 mutants progress into the meiotic divisions with high levels of γH2A foci

Our observations imply that unresolved joint molecules in the *smc5* and *nse4* cells cause severe failure of chromosome segregation during anaphase I and II and, ultimately, meiotic catastrophe (Figure 2). This recombination-dependent meiotic catastrophe hypothesis makes at least two predictions. First, the cell cycle should occur with similar timing in the mutant and wild-type strains and, second, individual meiotic nuclei should show increased DNA damage at anaphase I and anaphase II, when cells are attempting to divide their nuclei.

**Figure 8:**
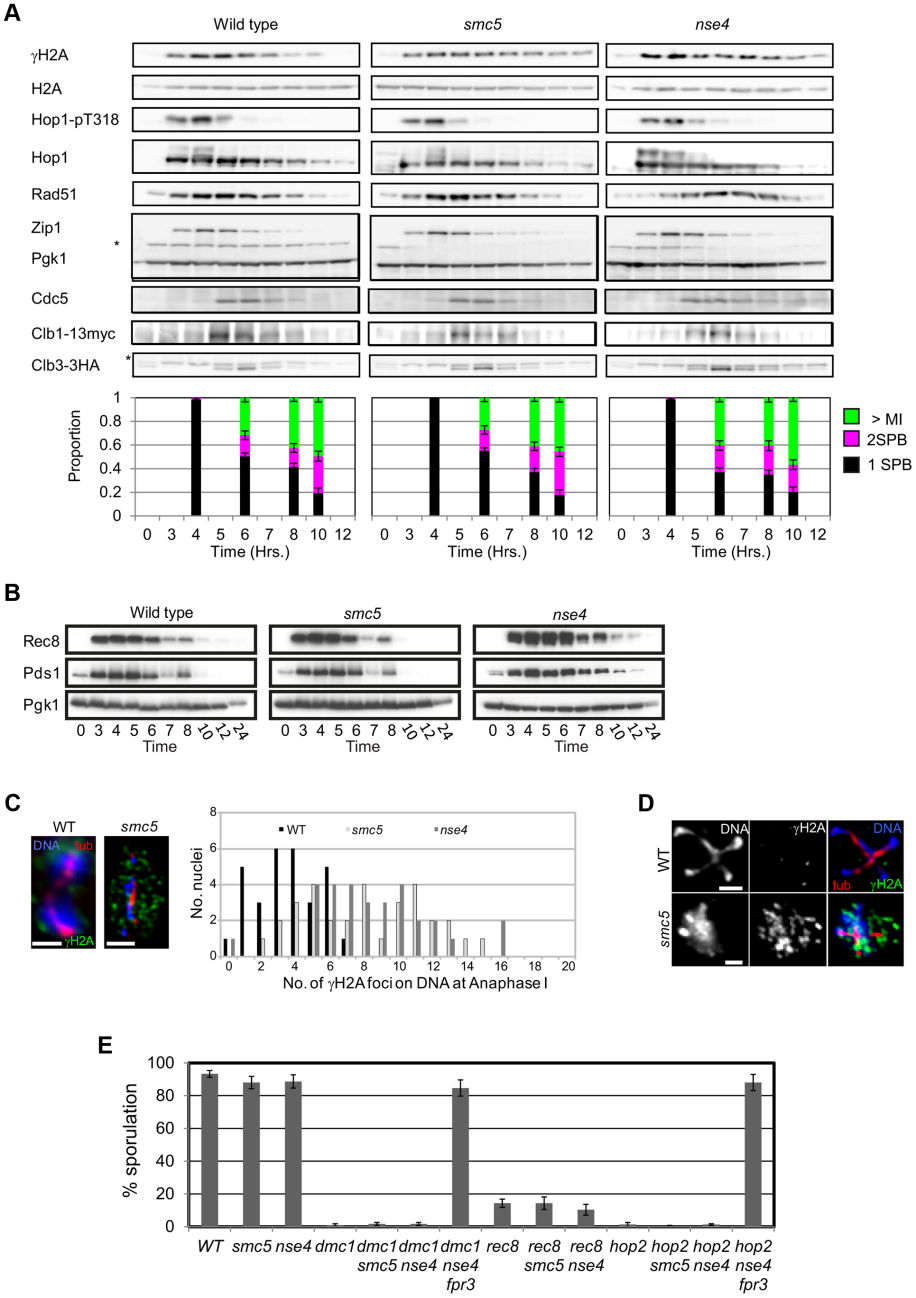
Smc5/6-depleted cells progress relatively normally through meiotic prophase I and enter nuclear divisions with damaged DNA. (A) Western blot analysis of Mec1 substrates, Hop1 (pT318) and H2A (pS129, γH2A). Clb1 and Clb3 are meiosis I- and meiosis II-specific B-type cyclins, respectively [101]. Pgk1 is a loading control. Strains: WT (Y4567), *smc5* (Y4570), and *nse4* (Y4573). Spindle pole body separation was used as a marker of cell cycle progression. (B) Western blot of Rec8-GFP and Pds1-13Myc. Strains: WT (Y2572), *smc5* (Y2673), and *nse4* (Y3653). (C) Typical examples of immunofluorescence images of γH2A at anaphase I from wild type and the two mutants. Bars: 2 µm. Right: quantification of the number of γH2A foci directly localized to the DNA. WT (Y1381), *smc5* (Y2704), and *nse4* (Y2705). (D) Typical examples of immunofluorescence images from wild type and *smc5* undergoing meiosis II. Bars: 2 µm. (E) Sporulation frequencies at 24 hours in *smc5* and *nse4* mutants in combination with mutants that show robust prophase I arrest. Strains: WT (Y1381), *smc5* (Y2704), and *nse4* (Y2705), *dmc1* (Y2045), *dmc1 smc5* (Y3491), *dmc1 nse4* (Y3488), *dmc1 nse4 fpr3* (Y4606), *rec8* (Y4607), *rec8 smc5* (Y2856), *rec8 nse4* (Y2855), *hop2* (Y2489), *hop2 smc5* (Y4610), *hop2 nse4* (Y4613), *hop2 nse4 fpr3* (Y4616).

To test these predictions, we monitored markers for early prophase I, exit from prophase I, and entry into meiosis II, which allowed us to calculate and thus compare transit times in the wild type to Smc5/6-depleted cells. Induction of the meiotic DNA damage response (DDR), monitored by the Mec1/ATR-dependent phosphorylation of HORMA-domain protein, Hop1, and γH2A [80], [81] occurred with similar timing, 3–4 hours after transfer to sporulation medium (Figure 8A). Spindle pole body separation, a marker for pachytene exit, and indeed spindle formation both occurred with relatively normal timing in the two mutants compared to wild type (Figure 8A). Consistent with this, the timing of Cdc5 and Clb1 expression, both under the regulation of the Ndt80 transcription factor that facilitates pachytene exit [67], were also similar in all three strains. These results suggest that exit from pachytene occurred with similar timing in the *smc5* and *nse4* mutants compared to the wild type strain.

To follow M-phase events, we assessed steady-state levels of Rec8 and Pds1, the securin orthologue in budding yeast. Degradation of both occur at the onset of anaphase I and anaphase II. Rec8 and Pds1 degradation occurred around 7 hours in all three strains and the second wave of Pds1 degradation (anaphase II onset) was observed in both wild type and *smc5* (Figure 8B). The *nse4* time course was presumably less synchronous such that the second wave of Pds1 and Rec8 degradation was not detected [82]. To assess meiosis II entry, we used the B-type cyclin, Clb3. In all three strains, Clb3 expression appeared at similar times (Figure 8A). Collectively, these observations strongly support the notion that the meiotic progression is not significantly delayed or arrested in Smc5/6-depleted cells.

The population kinetics of γH2A suggest that *smc5* and *nse4* mutants undergo meiotic catastrophe with damaged DNA. In the wild-type, γH2A disappeared by 7–8 hours, whereas it remained high in the two mutant strains, even at 12 hours when meiosis was completed (Figure 8A, and data not shown). Consistent with this analysis, immunostaining for γH2A foci in combination with tubulin revealed meiosis I and meiosis II cells that also contained an increased number of γH2A foci (Figure 8C,D). In the wild type, cells with anaphase I spindles showed confluent, low intensity background γH2A staining as well as a few punctate foci (median: 3 foci). In contrast, analogous nuclei from both *smc5* and *nse4* mutants contained large numbers of γH2A foci, many of which were located off the main body of DNA (Figure 8C), suggestive of perturbed DNA/chromatin structure. Furthermore, in nuclei with meiosis II spindles, 5% of *smc5* and 42% of *nse4* nuclei (n = 50) contained punctate γH2A staining (Figure 8D). The lower number of γH2A-positive staining anaphase II nuclei in the *smc5* mutant presumably reflects the lower level of unresolved joint molecules relative to *nse4* (Figure 5). Collectively, these data indicate that *smc5/smc6* mutants progress through the meiotic divisions with elevated levels of γH2A.

Finally, we investigated whether *smc5* and *nse4* mutants are deficient in maintaining the DDR-induced meiotic arrest that occur in mutants, where high levels of single-stranded DNA accumulate (*dmc1Δ*, *rec8Δ*, and *hop2Δ*) [83]. Depletion of Smc5 or Nse4 had no effect on the meiotic progression in any of these mutants (Figure 8E). Combining the *dmc1Δ nse4* or *hop2Δ nse4* mutants with *fpr3Δ*, which is required for checkpoint maintenance [84], resulted in high levels of checkpoint bypass (Figure 8E). These data demonstrate that *smc5* and *nse4* mutants are checkpoint proficient and that the progression into the meiotic nuclear divisions with unresolved joint molecules is unlikely to be caused by defective DDR maintenance.

### Meiotic cohesin is mis-regulated in *smc5* and *nse4* mutants

**Figure 9:**
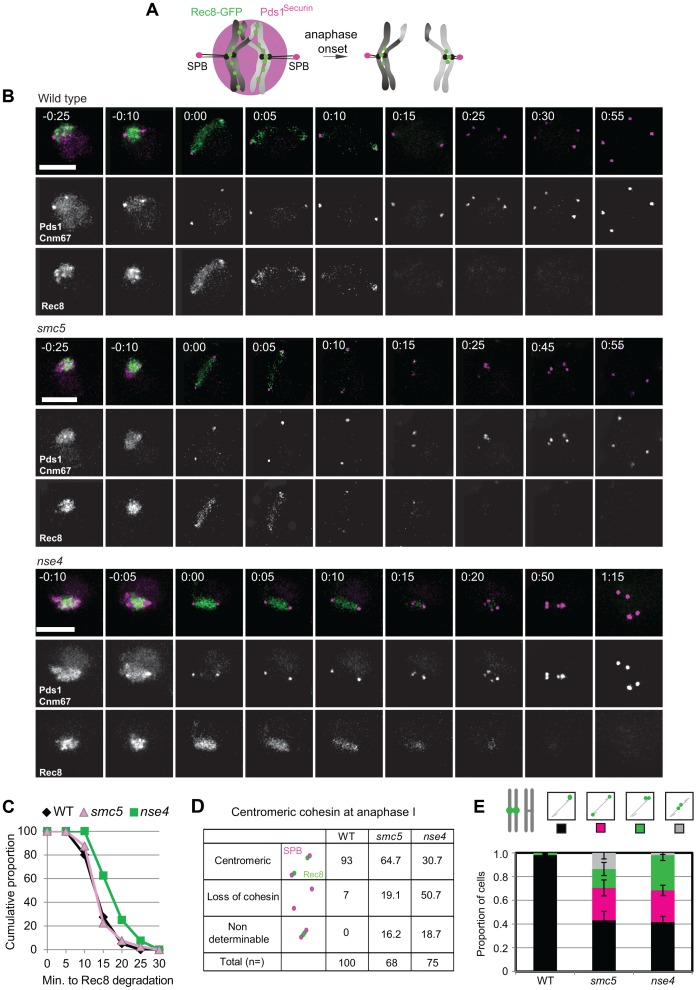
Misregulation of cohesin in *smc5/6*-depleted cells. (A) Experimental set up: Spindle pole body component CNM67-mCherry and Pds1^Securin^-tdTomato were used to assess spindle length and the onset of anaphase I, respectively. Rec8 is tagged with GFP. Upon anaphase I onset, Pds1^Securin^-tdTomato is degraded, the distance between CNM67-mCherry foci increase, and Rec8-GFP is degraded along arm regions until only centric and pericentromeric cohesin is left (right hand diagram). (B) Typical examples of time lapse images from wild type and the two mutants. Bars: 4 µm. Arrows indicate loss of centromeric cohesin signal. Note that the temporal resolution of kinetics is limited to 5 min. Strains: WT (Y2572), *smc5* (Y2673), and *nse4* (Y3047). Full movies are available in the Supplemental Information (Movies S5, S6, S7). (C) The cumulative proportion of cells with arm cohesin has been degraded at the given time after anaphase I onset (n≥40 per strain). Significance tests for Kruskall-Wallis (P<0.01) show *nse4* is delayed compared to wild type and *smc5*. (D) Proportions of nuclei with centromeric cohesin at anaphase I from live-cell imaging experiments. Anaphase I was staged by loss of Pds1 signal. (E) Analysis of sister kinetochore separation. tetO repeats are inserted 1.5 kb from *CEN5* and tetR-GFP expressed constitutively. Only one homolog contains the tetO-*CEN5* insertions, which allows analysis of sister kinetochore behaviour. Bars represent standard error (n>100 for each strain). Anaphase I was staged by spindles being greater than 4 µm in length. At this length, all spindles from *smc5* and *nse4* were Pds1 negative (data not shown). WT (Y2708), *smc5* (Y2709), and *nse4* (Y3071).

Unresolved joint molecules are inferred to impede chromosome separation in cells undergoing the meiotic divisions [11], [12]. However, cleavage of cohesin by separase is also essential for chromosome disjunction [85]. Smc5/6 localizes to cohesin-binding sites (Figure 1) and in *S. pombe*, *smc5/6* mutants show increased retention of cohesin during mitosis that contributes to chromosome segregation defects [86], [87]. These considerations led us to evaluate whether cohesin was mis-regulated in meiosis. To this end, we analysed Rec8-GFP dynamics in time-lapse studies [88]. Using Pds1-tdTomato as a marker for anaphase I entry (Figure 9A), cohesin removal along chromosome arms was completed in 14.2 (±5.7) minutes in wild type (n = 30; Figure 9A & B, Movies S4, S5, S6, S7). There was little or no delay in the *smc5* cells and a slight but significant delay in the *nse4* mutant (Figure 9C, Mann-Whitney p<0.01). Assessment of retention of cohesin in spread nuclei confirmed that the cohesin was associated with meiotic chromosomes (Figure S8). Moreover, we also observed *smc5* nuclei at anaphase II with significant cohesin staining (Figure S8B). It is likely that this residual cohesin that we detect with antibodies but not live cell imaging in the *smc5* mutant, reflect relatively low levels of retained cohesin that cannot be detected due to the decreased sensitivity of live cell imaging.

**Figure 10:**
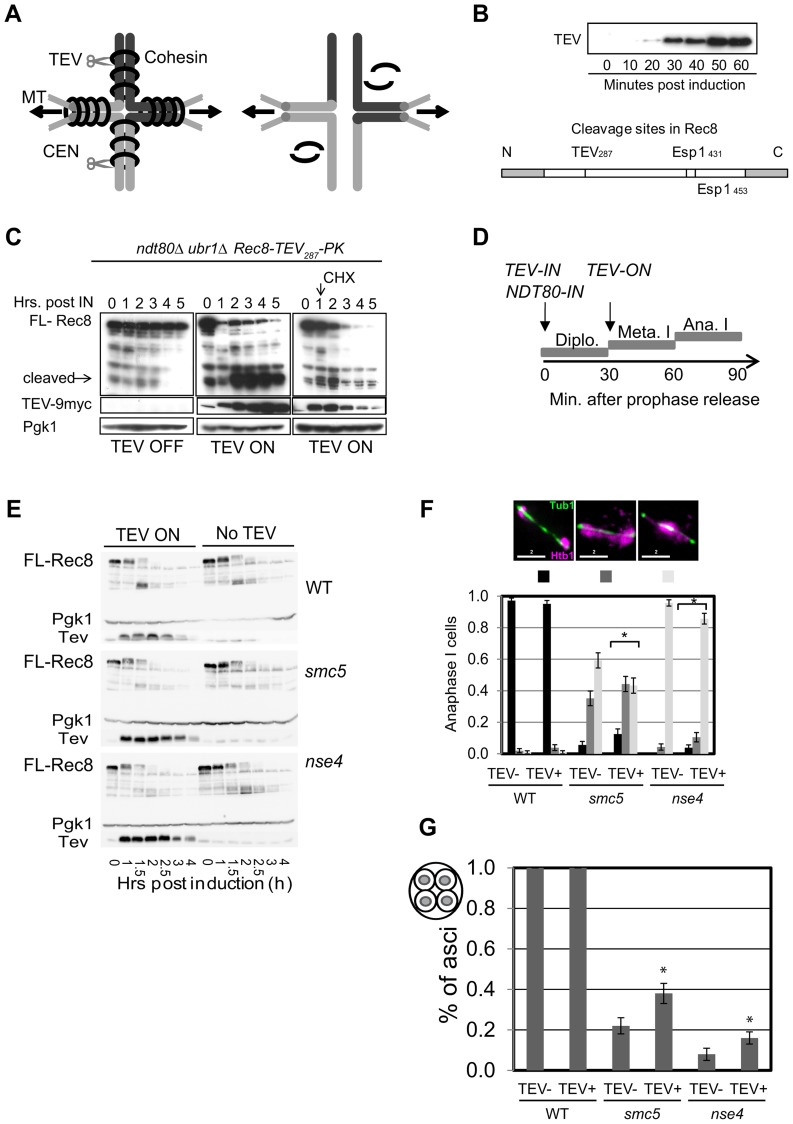
Retained arm cohesin at anaphase I contributes to the chromosome resolution defect in the *smc5* and *nse4* mutants. (A) Diagram of bivalent resolution by cohesin (Rec8) cleavage along arms regions Abbreviations: MT-microtubules, CEN-centromeres, scissors depict TEV protease. (B) TEV-9Myc expression after induction during a meiotic time course and the TEV cleavage site introduced into Rec8. Note that Rec8-TEV287-PK retains its two separase (Esp1) cleavage sites. (C) Rec8-TEV287-PK cleavage by TEV protease in *ndt80*Δ *ubr1*Δ cells. TEV protease was induced 6 hours into meiosis when >80% are arrested in pachynema. FL-full length Rec8-TEV287-PK. Left panel shows no TEV induction; the middle panels shows TEV induction; and the right panel shows TEV induction and cyclohexamide treatment (CHX) 1.15 hours after induction. Pgk1 was used as a loading control. Strain: Y3380. (D) Experimental set up of TEV protease induction after meiotic prophase by simultaneous induction of TEV protease and prophase exit (*NDT80-IN*). (E) Analysis of protein levels of Rec8-TEV_287_-PK in arrested and released (*NDT80-IN*) cells. (F) Nuclear separation at anaphase I. Bar graph shows proportion of tetrads with fully separated, ‘stretched’ or compacted nuclear appearance. The *denotes statistically significant differences (p<0.01, G-test) in the distribution of classes. (G) DNA encapsulation into spores. Bar graph shows proportion of tetrads with fully encapsulated DNA. The *denotes statistically significant differences (p<0.05, G-test) in the distribution of classes. Strains: WT (Y3264- no TEV and Y3299), *smc5* (Y3261- no TEV and Y3237), and *nse4* (Y3258- no TEV and Y3240).

To address whether the delayed removal of cohesin relative to the nuclear divisions contributed towards the severe chromosome segregation defects of the *smc5/6* mutants, we engineered a TEV protease cleavage site into Rec8 (in addition to the two separase cleavage sites) and expressed TEV protease around anaphase I onset (Figure 10A–D). We observed small improvements in chromosome segregation at anaphase I in both strains, with a more pronounced effect in *smc5* (Figure 10F, G). However, the contribution of the persistent cohesin towards the severe meiotic catastrophe is likely relatively small compared to the failure to remove joint molecules prior to the meiotic divisions, especially in the *nse4* strain.

Finally, we noticed that the retention of centromeric cohesin was severely defective in the two mutants (Figure 9, S8A, C). This premature loss of centromeric cohesin correlated with the precocious separation of sister centromeres (Figure 9E) and indicates that *smc5/6* mutants experience problems with the establishment and/or retention of cohesion. We conclude that the mis-regulation of cohesin is two-fold in the Smc5/6-depleted cells: removal of arm cohesin is delayed while the protection of centromeric cohesin is compromised as well.

## Discussion

### The Smc5/6 complex is essential for chromosome segregation in following the induction of DSBs in meiosis

SMC complexes regulate a vast array of chromosomal processes, including DNA repair, during mitosis and meiosis [37]. In this study, we set out to determine whether the third, highly conserved SMC complex, Smc5/6, has roles in meiotic recombination. We were particularly interested in determining whether depletion of Smc5/6 leads to general recombination defects, like cohesin or condensin [89], [90], or whether specific pathways would be perturbed in its absence (Figure S9).

Despite its central role in mitotic cells in mediating resolution and separation of chromosomes in response to DNA damage, the role of the Smc5/6 complex in meiotic recombination has remained enigmatic. Previous findings suggested that Smc5/6 mediated its critical role during premeiotic S-phase, since deletion of *SPO11* did not alleviate the chromosome separation defect of *smc6* temperature-sensitive mutants [54]. In this work, we show clearly that the budding yeast Smc5/6 complex is required for chromosome resolution following induction of meiotic recombination (Figure 3). Similar findings are reported by two independent studies in budding yeast [61], [73]. Collectively, they firmly support the notion that across a range of species, Smc5/6 has essential functions in mediating chromosome resolution in response to induction of meiotic recombination [55], [58], [61], [73], [91].

### Recombination-induced meiotic catastrophe in *smc5/6* mutants is caused by a combination of three factors

**Figure 11:**
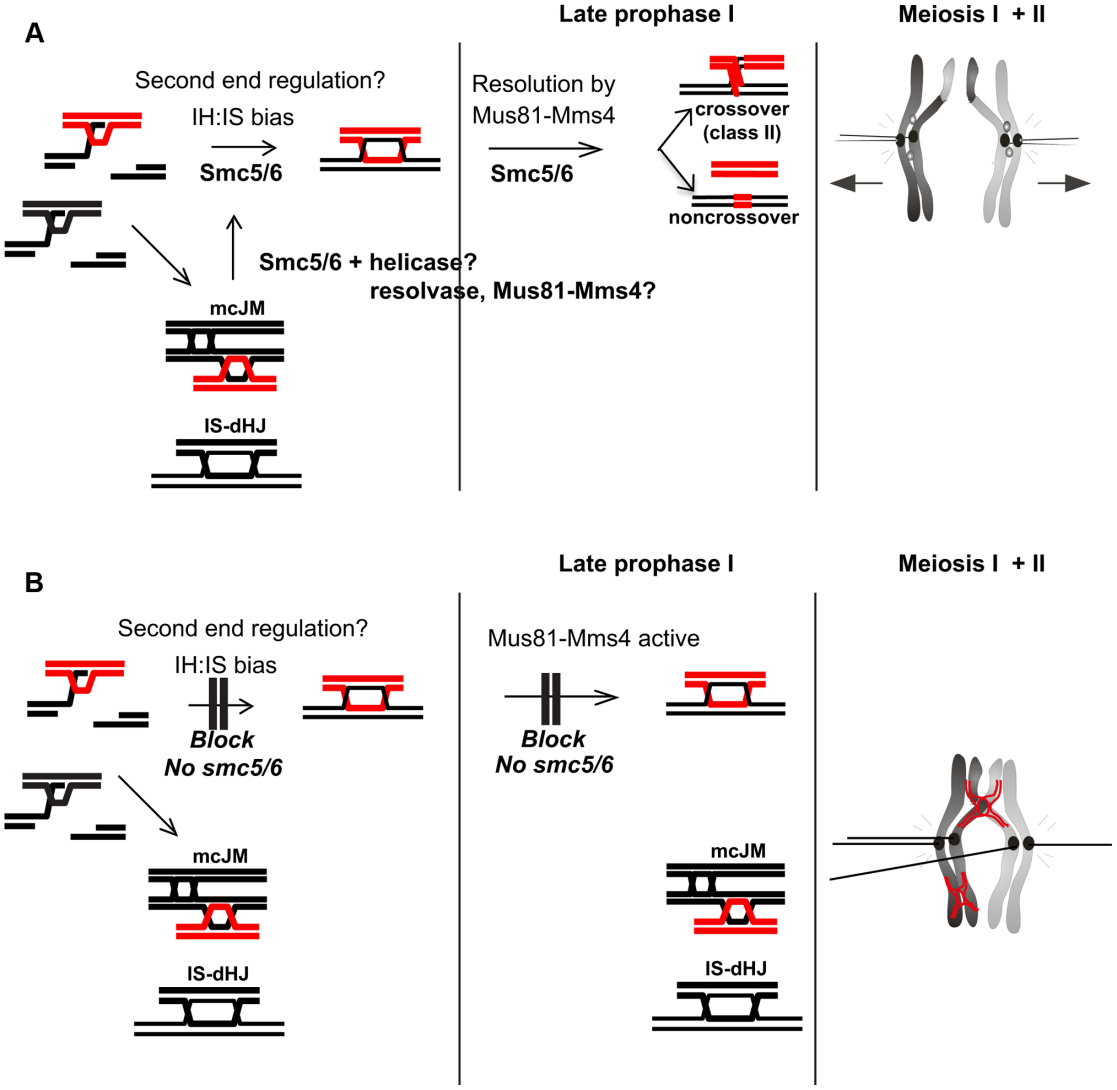
Model for Smc5/6 function during meiosis. (A) In wild type cells, Smc5/6 is present and ensures the formation of IH-dHJs either directly or perhaps by removing mcJMs and IS-dHJs, returning them to an interhomolog fate. This could be done in co-operation with helicases and resolvases, potentially Mus81-Mms4. (B) In the absence of Smc5/6, second end regulation is aberrant and cells enter late prophase with increased mcJMs and IS-dHJs. These are not cleaved by Mus81-Mms4, which is hyperphosphorylated by Cdc5, because it requires Smc5/6. Since the joint molecules do not appear to trigger a prophase I checkpoint, *smc5/6* mutants enter the nuclear divisions with joint molecules as well as precociously separated sister kinetochores that prevent chromosome segregation, leading to meiotic catastrophe.

During meiosis, Smc5/6 localizes to centromeres, cohesin-binding sites and sites of meiotic DSBs (Figure 1). However, the chromosome-length dependent increase in the density of Smc5/6 binding sites reported in vegetative cells [60] is not observed in meiosis. We identified at least three factors that contribute to the general failure of chromosome separation seen in *smc5/*6 mutants. First, high levels of joint molecules, both between homologs and sister chromatids, remain unresolved, especially in the *nse4* mutant (Figure 5). Second, cells enter the meiotic nuclear divisions without a delay that might otherwise allow time for joint molecules to be resolved (Figure 8). Third, mis-regulation of cohesin also partly contributes to the delayed chromosome separation at anaphase I, especially in the *smc5* mutant (Figure 10). Moreover, a combination of unresolved joint molecules between sister chromatids and precocious separation of sister kinetochores (Figure 9) could also contribute to chromosomal entanglement (Figure 11B).

Time-lapse imaging of single cells delineates the sequence of severe chromosome segregation defects and meiotic catastrophe caused by unresolved joint molecules. Meiotic catastrophe was preceded by failure to separate the nuclear mass (*nse4*) or by failure to keep the nuclear masses separated upon spindle disassembly (*smc5*). Spindle formation and elongation were associated with aberrant chromosome morphology such as micronuclei and chromosome spikes (Figure 2).

It has been suggested that even low levels of unresolved joint molecules may block chromosome separation in meiotic cells [11], [12]. In the *nse4* mutant, the 10% of chromosomes trapped in joint molecules at *HIS4LEU2* (Figure 5) translates to 20% of cells with an unresolved joint molecule at this recombination hotspot. Assuming that naturally occurring hotspots display a similar dependency on Smc5/6, each cell will undergo nuclear divisions with 20%, or roughly 30–40 joint molecules, unresolved (based on DSB levels of 150–200 per cell [92]). In the *smc5* mutant, the 1.8% unresolved joint molecules at 13 hours would equate to ∼5–7 persistent joint molecules per cell. These considerations raise the possibility that a small number of unresolved joint molecules (less than one per chromosome) can cause a pan-nuclear segregation defect.

### Smc5/6 is critical for joint molecule metabolism at meiotic DSB hotspots

Physical monitoring of joint molecules indicates that Smc5/6 regulates both the formation of recombination intermediates as well as their resolution (Figure 5) [61]. In accompanying studies the hypomorphic *smc6–56* allele and the SUMO E3 ligase-dead *mms21-11* alleles also accumulate joint molecules [61], [73]. Therefore, inactivation or depletion of four distinct components of the core budding yeast Smc5/6 complex leads to defective joint molecule metabolism during meiosis. Similarly, in *S. pombe*, *nse5* and *nse6* mutants show accumulation of Rec12/Spo11-dependent joint molecules [55]. Thus, Smc5/6 has a critical and conserved role in the completion of meiotic DSB repair in both yeasts by facilitating the removal of joint molecules.

### Smc5/6 has critical roles in regulating the orderly formation of recombination intermediates during meiotic prophase I

We have identified three aberrations in the joint molecules that accumulate in the *smc5/6* mutants from which we infer that Smc5/6 is critical for directing not only the removal of joint molecules upon prophase I exit (‘late prophase I”, Figure 11), but also their proper formation during DSB repair (Figure 11). Smc5/6 depletion increases the fraction of joint molecules between sister chromatids that involve three and four chromatids (multi-chromatid JMs), while decreasing the levels of interhomolog dHJs. A similar conclusion is reached by Xaver *et al.* (2013), who analyzed joint molecules at a second hotspot. Since single-end invasions formed relatively normally in *smc5/6* mutants (Figure 5), these observations suggest that Smc5/6 may be important for coordinating the two DSB ends or to limit secondary strand invasions between sister chromatids (Figure 11A). Smc5/6 could also redirect multi-chromatid JMs and intersister dHJs to the interhomolog fate, perhaps via regulation of DNA helicases and/or endonucleases during early prophase I (Figure 11A). Such a redirection process was previously envisioned for Sgs1 [10].

Mus81-Mms4 was previously shown to play a small but significant role in inter-homolog bias, primarily by enhancing formation of inter-homolog dHJs [12], [30]. Since inactivation of Mus81-Mms4 did not cause a further decrease in inter-homolog bias in the *nse4* mutant (Figure 7), it is possible that Smc5/6 regulates this function of Mus81-Mms4 during the formation of interhomolog dHJs. However, Mus81-Mms4 also somehow increases the final level of unresolved joint molecules in *nse4* cells (Figure 7E). Perhaps, in the absence of Smc5/6 function, Mus81-Mms4 creates structures that cannot be resolved. Alternatively, the decreased accumulation of JMs in the *nse4 mms4* and *nse4 mus81* mutants may suggest functions of Mus81-Mms4 in processing DSB repair intermediates that do not lead to crossovers (see below).

### Aberrant joint molecules species accumulate in *smc5/6*

Inspection of the JM spots revealed additional spots and smears of the main dHJ molecules, suggestive of altered structure of the JMs that accumulate in *smc5/6* mutants (Figure 5B). In *S. pombe*, JMs that accumulate in *mus81* mutants can be resolved *in vivo* by expression of RusA and by RuvC after extraction from gels. In *nse5/6* mutants, however, the JMs appeared partially refractory to both RusA and RuvC treatment [55], although they migrated in similar spots of JMs in *mus81* mutants. Our observations suggest that the JMs that are formed in *smc5/6* mutants are not normal and this, together with the mislocalization of Mus81-Mms4 on the meiotic chromosomes, could contribute to the lack of resolution by Mus81-Mms4, despite its normal activation by Cdc5.

### Smc5/6 regulates Mus81-Mms4-dependent resolution of joint molecules, whilst MutLγ remains active

In *S. pombe*, Mus81-Eme1 promotes most or all crossovers and deletion of Nse5 or Nse6 diminishes crossing over [27], [28], [55], [57]. Our findings show that Smc5/6 may be specifically required for resolution mediated by structure-specific endonucleases such as Mus81-Mms4 (and possibly also Yen1 and Slx1–Slx4) in organisms with alternative resolving pathways. Specifically, we found that crossover levels and inter-homolog bias in *nse4* mutant were not further reduced when Mus81-Mms4 was also mutated (Figure 7D,E). In contrast, mutation of Sgs1 or Mlh3 synergistically reduced crossover levels in *nse4* cells (Figure 6). These observations suggest that Smc5/6 coordinates resolution of joint molecules that form independently of the major, MutSγ-dependent pathway. It is possible that Smc5/6 affects resolution of all non-Msh4/5 joint molecules. We infer that it is unlikely that Smc5/6 depletion leads to gross, general chromosomal defects that generally affect recombination, as seen in condensin mutants, where Cdc5/Polo-like kinase fails to associate with meiotic chromosomes and recombination is perturbed [90], [93].

How might Smc5/6 regulate joint molecule resolution? In the case of Mus81-Mms4, hyperphosphorylation and presumably hyperactivation of endonuclease activity still occurs in in the *smc5* and *nse4* mutants (Figure S7). However, association of Mus81 with meiotic chromosomes is diminished (Figure 7F,G), even during early prophase I, consistent with observed defects during the formation of joint molecules (Figure 7D,E). Although we do not know whether the Mus81 foci we observe reflect catalytically active Mus81-Mms4 complexes, our data support the idea that Smc5/6 mediates chromosomal association of Mus81-Mms4.

Smc5/6 has been reported to have low affinity interactions with single stranded DNA [94]. It is possible that the complex targets Mus81-Mms4 to substrates containing single-stranded regions. However, no direct interaction between Mus81-Mms4 and the Smc5/6 complex has been reported. Another possibility is that Smc5/6 holds joint molecules (or their precursors) in a conformation that ultimately allows resolution by Mus81-Mms4. In this regard, the novel joint molecule species that we detect in the *smc5* and *nse4* mutants may represent structures that cannot be resolved by Mus81-Mms4 or other resolving endonucleases. EM studies have revealed aberrant JM structures in *sgs1* and *mms4 sgs1* mutants that might represent hard-to-resolve structures [12]. Finally, Smc5/6 may also regulate local chromosome structure around a subset of DSBs and this could impact on recombination [86]. For example, mis-regulation of cohesin could indirectly influence inter-homolog bias, as seen in *rec8Δ* mutants [89].

## Materials and Methods

The SI contains Movie S1, S2, S3, S4, S5, S6, S7; nine additional Figures (S1, S2, S3, S4, S5, S6, S7, S8, S9); and one Table (S1).

### Yeast strains and meiotic time courses

Strains are described in Table S1. They are all derived from SK1.

Diploid cells were grown to saturation in YEPD (1% yeast extract, 2% bactopeptone, 2% dextrose, pH 6.5), then inoculated at 5×10^6^ cells per ml in SPS (0.05% yeast extract, 1% peptone, 0.17% YNB, 1% potassium acetate, 0.5% ammonium sulphate, 0.05 M potassium hydrogen pthalate at pH 5.5) and grown to a cell density of 5×10^7^ cells per ml. To induce meiosis, cells were resuspended in SPM (pH 7.0) consisting of 1% potassium acetate, 0.02% raffinose, 0.02% antifoam (Sigma, A8311), 2% histidine, 1.5% lysine, 2% arginine, 1% leusine and 0.2% uracil.

### Genome-wide Smc5 DNA binding and microarray analysis

Genome-wide Smc5 association was measured as previously published [95]. Briefly, Smc5 crosslinked chromatin was immunoprecipitated with 2 µl anti-myc 9E11 (Abcam) or 20 µl anti-V5 beads (Sigma-Aldrich). Immunoprecipitated and input DNA samples were cohybridized to a custom DNA microarray (Agilent) and data were normalized as previously described. Every 3 points along the chromosome were averaged to produce the smoothed profiles in Figure 1. The relative enrichment of Smc5 to Rec8 and Smc5 in *spo11* versus *SPO11* is the ratio of the values in each of the two datasets indicated. The raw data and log ratios from this study are available from the NCBI Gene Expression Omnibus (http://www.ncbi.nlm.nih.gov/geo/), accession number GSE44852.

### Molecular assays

Molecular assays were carried out as described previously [72], with the modification that we used the Phase Lock Gel for phenol extraction. We analysed three independent diploids for each strain.

### CHEF analysis of chromosome breakage

To measure genome wide DSB signal, chromosome-length DNA captured in agarose plugs [96] was separated by pulsed field gel electrophoresis under the following conditions: 1.3% agarose in 0.5×TBE; 14°C; 6 V/cm; switch angle 120°, ramped switch time of 15–25 seconds over 30 hours (Biorad CHEF DRIII). Following a denaturing transfer to nylon membrane, a radioactive DNA telomeric probe for the left side of chromosomes III (*CHA1*) was hybridized to the membrane. Radioactive signal was collected on phospho-screens, imaged using a Fuji FLA5100 and quantified using FujiFilm ImageGauge software. DSB signal was measured as a percentage of the total lane signal [97]. DSB molecules occurring further from the probe are under-estimated due to DSBs occurring closer to the probe on the same molecule. To correct for this, the estimated DSB frequency was calculated using Poisson correction: Percentage broken chromosomes (Poisson corrected) = −ln(1−measured DSB signal). To produce lane profiles, 900 lane slices were exported from ImageGauge and combined from 6–10 hours and each slice plotted as a percent of total lane signal.

### Yeast protein extraction & protein analysis

Cells from meiotic cultures (OD_600_ 1.2–1.5, 2 ml) were disrupted using glass beads in 200 µl of ice cold 20% TCA. Precipitates were collected by centrifugation and washed in 400 µl of ice cold 5% TCA. Precipitates were resuspended in 100 µl of SDS-PAGE sample buffer (4% SDS, 5% β-mercaptoethanol, 0.15 M DTT, 20% glycerol, 0.01% bromophenol blue); boiled for 5 minutes at 95°C, centrifuged, and the supernatant containing protein was collected.

Proteins were separated by SDS-PAGE, transferred to nitrocellulose membranes, and probed with the appropriate antibodies followed by HRP-conjugated secondary antibodies (DAKO, 1∶2000). HRP activity was detected using Pierce ECL Western Blotting Substrate followed by exposure to Amersham Hyperfilm ECL or using the Image Quant LAS 4000 imaging system.

### Antibodies used for western blotting

Cdc5 (Santa Cruz sc-6732, 1∶2000), HA (12CA5, CRUK, 1∶1000 or Abcam Ab9110, 1∶1000), γH2A (J. Downs, 1∶1000), H2A (1∶5000, J. Downs), Rad51 (1∶2000, S. Roeder), PAP (Sigma P1291, 1∶2000), Pgk1 (Invitrogen 459250, 1∶200 000), Myc (9E10, CRUK, 1∶2000), V5 (AbDSerotec MCA1360, 1∶2000), Zip1 (Santa Cruz sc-48716, 1∶2000), Hop1 (F. Klein, 1∶1000), pHop1-T318 (Cambridge Research Biochemicals, 1∶500), and Clb3 (Santa Cruz sc-7167, 1∶500).

### TEV protease induction

Meiotic cultures were arrested at pachynema after 6 hours in SPM. TEV protease and Ndt80 were induced by the addition of 1 µM β-estradiol.

### Protein synthesis block

Protein synthesis was blocked by the addition of cyclohexamide to meiotic cultures to a final concentration of 200 µg/ml. Cyclohexamide was added to meiotic cultures 1 hour after Ndt80 induction.

### Auxin-dependent degradation of Smc5

The *P_CLB2_-SMC5* was C-terminally-tagged with the AID [69]. To induce degradation of Smc5, we added 150 µl of 500 mM auxin (3-indoleacetic acid; Sigma I375-0), resuspended in 1N NaOH, to 50 ml meiotic cell cultures. This was added at 1 hour after transfer to SPM. Addition of auxin at earlier time points resulted in arrest during the preceding mitotic divisions when cells underwent premeiotic growth in pre-sporulation medium (SPS).

### Meiotic nuclear spreading, immunofluorescence, and antibodies

Nuclear spreading and antibodies have been described elsewhere [98], [99], except that we treated cells with both zymolyase 100T and glusulase in order to generate spheroblasts for some strains. Fixation followed by indirect immunofluorescence was carried out by fixing cells in 4% formaldehyde for 15–45 minutes at room temperature.

When assessing Mus81-Mms4 foci, we carefully controlled for the extent of spreading, because we noted that even in the wild type, a small proportion of nuclei did not contain Mus81-Mms4^Eme1^ foci. When we applied more extreme spreading techniques, all Mus81-Mms4^Eme1^ staining (but not Zip1) was abolished in the wild type (data not shown). This suggests that the Mus81-Mms4^Eme1^ interaction with meiotic chromosomes is less stable than Zip1.

### Live cell imaging

Cells were initially incubated in sporulation media for 6–8 hours. 20 µl of cells were added to a Y04D CellASIC plate (CellASIC ONIX microfluidic perfusion system) and imaged inside an environmental chamber set at 30°C. A flow rate of 8 psi was used to load the cells and a steady-state flow rate of 2 psi was used for the duration of the time course.

Time-lapse microscopy was carried out using a Personal DeltaVision (Applied Precision) with xenon or solid-state illumination, using associated proprietary software (SoftWoRx software; version 4.0.0, Applied Precision). Images were captured using an UPLS Apochromat 1.4 numerical aperture, ×100 magnification oil immersion objective (Olympus), auxiliary magnification to prevent undersampling, standard DeltaVision filter sets FITC (ex 490, em 525 nm) and TRIC (ex 555, em 605), yielding approximate resolutions (Rayleigh's d) of ∼229 nm and 264 nm in the xy, respectively, whereas axial resolutions were approximately 811 and 935 nm. Photon detection was carried out using a Cascade2 1 K EMCCD camera (Photometrics) using a gain of 230 and no binning. Images were taken using exposure times of 0.025 sec. and 32% transmission (FITC) and 32% transmission and 0.1 sec. exposure (TRITC). 6–7 z-stacks at 1 µm were collected. Final images for sporulation were carried out with DIC, 32% transmission and 0.05 sec. exposure. Images were recorded every 5 minutes for the first 90 minutes, every 20 minutes for the next 80 minutes and then every 45 minutes for the last 90 minutes. Around 12 hours after imaging the sporulation of the cells at each point of imaging was assessed. Only cells that sporulated were included in the analyses.

### Image analysis and manipulation

Images were deconvolved using SoftWoRx software (version 4.0.0, Applied Precision). Subsequent 3D analysis to measure spindle length was carried out using Imaris (version 7.0.0, Bitplane).

3D images are presented as maximum projections, rendered in Softworx or Imaris. Some images were manipulated in Adobe Photoshop CS5.1 using the following procedure. Images were converted to .psd files from *Softworx* files before being opened in Adobe Photoshop. Only the max/min input levels of each channel were adjusted manually to adjust differences in the imaging intensities. Images were cropped preserving the relative ratios, and the size bar copied to a second layer of the image. For aesthetic reasons, a broader bar covering the size and the out-of-focus number was added on top of the original. Analysis of foci numbers was carried out manually and with the ‘Find Peaks algorithm’ (ImageJ plugin is available from: http://www.sussex.ac.uk/gdsc/intranet/microscopy/imagej/plugins and documentation: http://www.sussex.ac.uk/gdsc/intranet/microscopy/imagej/findpeaks). Peaks were identified above a background level using non-maximal suppression. An allowance was made for peak regions covering multiple pixels with the same intensity (plateau maxima). A watershed algorithm was used to assign all non-maxima pixels to the appropriate peak by following the maximum gradient. Peak expansion was restricted using the height above background. Following identification the boundaries between peaks were calculated and the highest boundary point between touching peaks stored as saddles. A peak merge algorithm was used to join insignificant smaller peaks into their neighbour peak defined using the highest saddle point. Peaks were identified as insignificant using height and area criteria.

Noisy data were smoothed using a Gaussian blur prior to peak identification. Reported peak statistics always use the intensity values from the original unsmoothed image. The algorithm can be applied to 2D or 3D images and is available as a plugin for ImageJ. The plugin allows setting parameters to control the background identification, search method, merge criteria and the results output. The plugin is scriptable via the ImageJ macro facility and provides a GUI that allows the parameters to be adjusted with real-time results update. The plugin will be published separately elsewhere.

### Statistics

We used various statistical tests in R (www.r-project.org), as indicated throughout the text. P-values were adjusted for multiple pair-wise comparisons according to Dunn-Sidak to reflect α<0.05. Standard error bars around proportions were calculated as √[p(1−p)/n], where p is the proportion of the specific class (n>100 for each strain). For the Pearson product-moment correlation, the cor.test uses the t-statistics to calculate the p-value and the Fisher z transform to generate an asymptotic confidence interval (95%).

## Supporting Information

Figure S1 Smc5-13myc localization on meiotic chromosomes. (A) Expression of Smc5-13myc and Nse4-TAP during meiosis. Note the Smc5-13myc band travelling with lower electrophoretic mobility (indicated by the arrow); likely the sumoylated species of Smc5. Strain: Smc5-13myc (Y2824), and Nse4-TAP (Y2826). (B) Localization of Smc5-13myc and Zip1. Note the lack of apparent colocalization during leptonema and zygonema. (C) Localization of Smc5-13myc in *rec8Δ* and *spo11Δ* mutants. Strains: *rec8Δ* (Y2837) and *spo11Δ* (Y2836). (D) Depletion of Cdc6 expressed under the *SCC1* promoter (left) and expression of Smc5-13myc. Strain: (Y2891). (E) Lack of localization of Smc5-13myc to chromosomes in *P_SCC1_-CDC6* strain. (F) Depletion of Top2 expressed under the *CLB2* promoter (left) and expression of Smc5-13myc. Note, this strain arrests at pachynema. Strain: (Y2851). (G) Diminished localization of Smc5-13myc to chromosomes in *P_CLB2_-TOP2* strain. Smc5-13myc foci numbers remained normal in *top1-mn*, *top3-mn*, *sgs1-mn*, *rad50S*, *dmc1*Δ, *zip1*Δ, *zip2*Δ, *zip3*Δ, *mer3*Δ, *pch2*Δ, *fpr3*Δ (data not shown).
(PDF)

Figure S2 Association of myc-tagged Smc5 with cohesin binding sites, centromeres, and DSBs. (A) DNA binding profiles for and Smc5-13myc (red, H5492) and Rec8-3HA (purple, H4471, [65]) plotted for Chromosome III. Lower panel shows enlarged, overlay on the right arm of Chromosome III (150–300 kb). (B) Overlay of the Rec8-3HA and Smc5-3V5 (Figure 1) or Smc5-13myc (A) binding profiles near *CEN3*. (C) The binding of Smc5-13myc was normalized to Rec8-3HA binding using the data shown in (A) to reveal weaker, non-core regions (red). DSB sites mapped by ssDNA enrichment are indicated below (blue, H118, [100]).
(EPS)

Figure S3 Auxin-induced degradation of Smc5-AID. (A) Western blot analysis of Smc5-AID-V5 after mock treatment or treatment with 1.5 mM auxin at 1 hour after transfer to sporulation medium. Strain: (Y4540). (B) Quantification of DNA encapsulation in Smc5-AID depleted cells. Note that continuous treatment with auxin leads to better depletion and a more severe phenotype, but that the mock-treatment with solvent (NaOH) alone (but not solvent+auxin) causes sporulation defects.
(PDF)

Figure S4 Meiotic recombination and crossing over in the *smc5 nse4* mutant is similar to the *nse4* single mutant. (A) Example of 1D analysis of crossover recombination. (B) Quantification of crossover levels from three independent diploids (24 hours). Strains: WT (Y2976), *smc5* (Y1211), *nse4* (Y1212), *smc5 nse4* (Y4179).
(PDF)

Figure S5 Smc5- or Nse4-depletion does not increase DSB levels in *RAD50S* or *dmc1*Δ mutants. (A) Representative CHEF gel followed by Southern blotting using the *CHA1* probe (chromosome III, left end) in *dmc1*Δ strain background. Percentage total lane signal was calculated by smoothing the histogram of signals from 900 bins in each lane. Strains: *dmc1*Δ (SG492), *nse4 dmc1*Δ (SG481), and *smc5 dmc1*Δ (SG478). (B) Quantification of DSBs (non-parentally sized fragments) are presented as raw data (left) or Poisson corrected (right, see materials and methods) for each time point. (C) Representative CHEF gel followed by Southern blotting using the *CHA1* probe (chromosome III, left end) in *RAD50S* strain background. Strains: *RAD50S* (SG488), *nse4 RAD50S* (SG484), and *smc5 RAD50S* (SG491). (D) Quantification of DSBs are presented as raw data (left) or Poisson corrected (right).
(PDF)

Figure S6 SC formation and disassembly occurs with normal kinetics in the *smc5* and *nse4* mutants. (A) Examples of Zip1 staining at pachynema in the wild type, *nse4* and *smc5* mutants. Strains: WT (Y967), *smc5* (Y3080) and *nse4* (Y2729). (B,C) Kinetics of Zip1 staining patterns and polycomplex formation (PC) in wild type and the *nse4* mutant. Left: Examples of Zip1 behaviour as ‘dotty’, ‘dot-linear’ and ‘linear’ staining, representative of leptonema, zygonema, and pachynema, respectively in nuclei from the *nse4* mutant (these are similar to those seen in wild type). The arrow indicates an aggregate of Zip1, likely a polycomplex (PC). Bars, 2 µm. Right: Proportion of nuclei with no Zip1, dotty, dot-linear, or fully linear Zip1 staining (upper panel) and the proportion containing a PC (lower panel). At least 100 nuclei were inspected for each time point. We chose a time course where spindle formation kinetics indicated similar synchrony in the two strains to allow direct comparison (not shown). The arrow denotes the time at which cells were released from prophase I arrest by induction of *NDT80* expression (*NDT80-IN*) allowing SC disassembly and Zip1 degradation (C) to be followed.
(PDF)

Figure S7 Steady-state levels and hyperphosphorylation of Mus81-9Myc and Mms4-9myc are not decreased in the Smc5-and Nse4-depleted strains. (A,B) Western blot of Mus81-9myc and Mms4-9myc. Loading factor Pgk1 was analysed on the same Western blot. Strains: WT (Y3618- Mus81-9myc, Y3683- Mms4-9myc), *smc5* (Y3621- Mus81-9myc, Y3689- Mms4-9myc) and *nse4* (Y3624-Mus81-9myc, Y3686- Mms4-9myc). (C) Mms4-9myc hyperphosphorylation occurs concomitantly with Cdc5 expression in wild type as well as the *smc5* and *nse4* strains. Pgk1 was used as loading factor.
(PDF)

Figure S8 (A) Immunostaining of fixed, semi-spread nuclei at anaphase I. Examples of anaphase I nuclei with associated Rec8-GFP along arms (‘arm retention’) as well as precocious loss of centromeric cohesin. Quantification is shown below. Anaphase I nuclei were staged by length; imaging with Pds1-tdTomato showed that all anaphase I spindles >4 µm were at anaphase I in wild type as well as the two mutants. (B) Representative images of Rec8-GFP of anaphase II nuclei in the wild type and *smc5* mutant. (C) Overexposure of the FITC (Rec8-GFP) channel to illustrate that the centromeric Rec8 is indeed not detected at anaphase I in *smc5* and *nse4* mutants. Box illustrates an anaphase I spindle (>4 µm). Overexposed GFP signals are from prophase I nuclei.
(TIF)

Figure S9 Integration of proposed Smc5/6 function within other JM regulatory mechanisms. The main crossover-generating mechanism is meiosis-specific and depends upon the preferential stabilization of recombination-intermediates by the ZMM proteins (green). Smc5/6 stabilizes other recombination intermediates and promote their resolution into both crossovers (class II) and noncrossovers by Mus81-Mms4 (grey box).
(EPS)

Movie S1 Time lapse imaging of nuclear divisions and spindle dynamics for wild type (Y3606). H2B is pseudocoloured in magenta and tubulin in green. This movie corresponds to panel 1 (upper panel) in Figure 2E.
(WMV)

Movie S2 Time lapse imaging of nuclear divisions and spindle dynamics for *smc5* (Y3627). H2B is pseudocoloured in magenta and tubulin in green. This movie corresponds to panel 2 in Figure 2E.
(WMV)

Movie S3 Time lapse imaging of nuclear divisions and spindle dynamics for *smc5* (Y3627). H2B is pseudocoloured in magenta and tubulin in green. This movie corresponds to panel 3 in Figure 2E.
(WMV)

Movie S4 Time lapse imaging of nuclear divisions and spindle dynamics for *nse4* (Y3630). H2B is pseudocoloured in magenta and tubulin in green. This movie corresponds to panel 4 in Figure 2E.
(WMV)

Movie S5 Time-lapse imaging of Rec8-GFP degradation in wild type (Y2572). Rec8-GFP is pseudocoloured in green; CNM67-mCherry and Pds1-tdTomato is shown in magenta. This movie corresponds to panel 1 in Figure 9B.
(WMV)

Movie S6 Time-lapse imaging of Rec8-GFP degradation in *smc5* (Y2673). Rec8-GFP is pseudocoloured in green; CNM67-mCherry and Pds1-tdTomato is shown in magenta. This movie corresponds to panel 2 in Figure 9B.
(WMV)

Movie S7 Time-lapse imaging of Rec8-GFP degradation in *nse4* (Y3047). Rec8-GFP is pseudocoloured in green; CNM67-mCherry and Pds1-tdTomato is shown in magenta. This movie corresponds to panel 3 in Figure 9B.
(WMV)

Table S1 List of strains used in this study. Individual strains used for the experiments are listed in the relevant figure legend.
(DOCX)
