# Supplementary figures and images for: Smc5/6 Coordinates Formation and Resolution of Joint Molecules with Chromosome Morphology to Ensure Meiotic Divisions
Source: PLoS Genet. 2013 Dec 26;9(12):e1004071. doi: 10.1371/journal.pgen.1004071 (PMC3873251; doi:10.1371/journal.pgen.1004071)

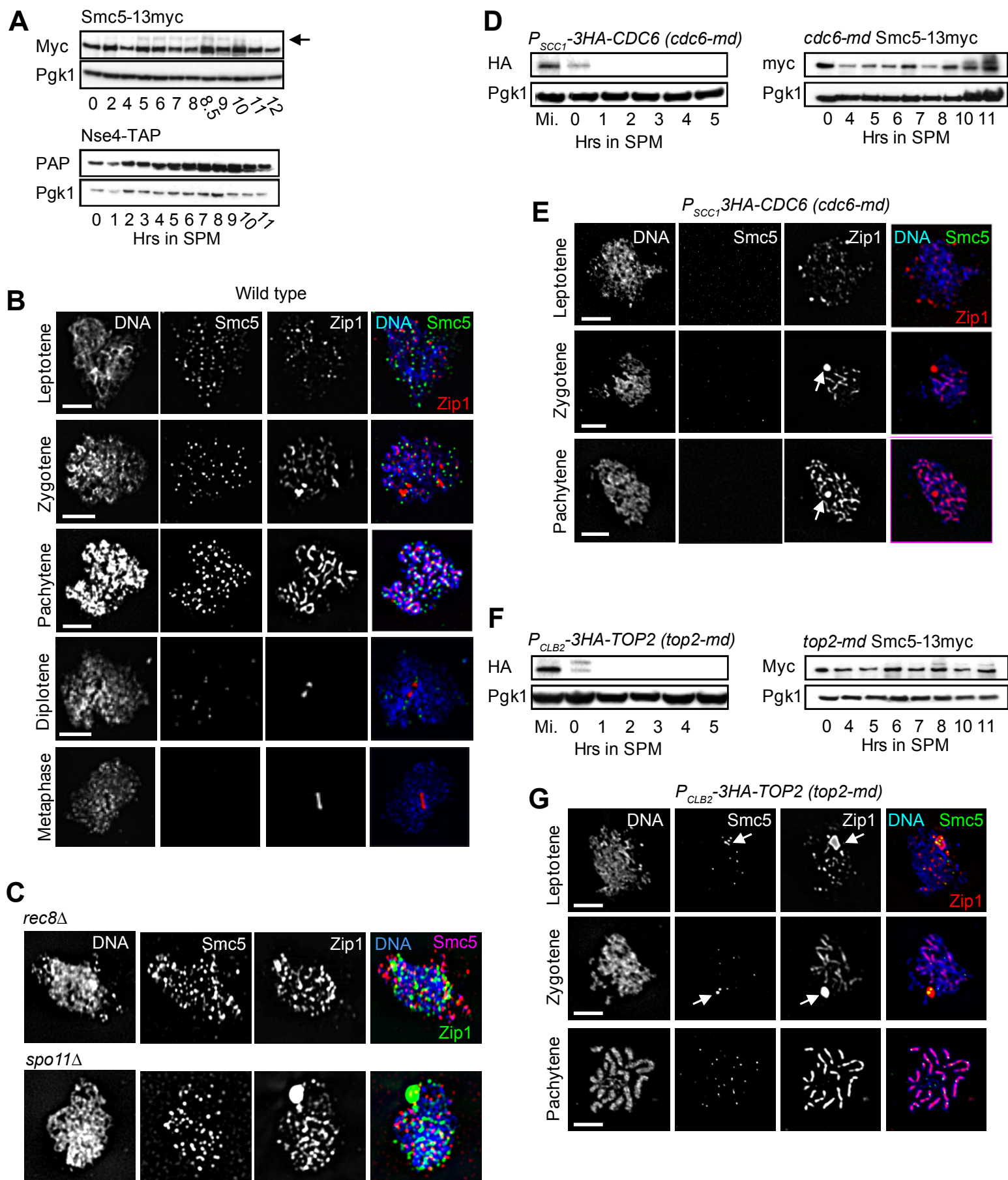

Supplement: Figure S1 — Smc5-13myc localization on meiotic chromosomes. (A) Expression of Smc5-13myc and Nse4-TAP during meiosis. Note the Smc5-13myc band travelling with lower electrophoretic mobility (indicated by the arrow); likely the sumoylated species of Smc5. Strain: Smc5-13myc (Y2824), and Nse4-TAP (Y2826). (B) Localization of Smc5-13myc and Zip1. Note the lack of apparent colocalization during leptonema and zygonema. (C) Localization of Smc5-13myc in rec8Δ and spo11Δ mutants. Strains: rec8Δ (Y2837) and spo11Δ (Y2836). (D) Depletion of Cdc6 expressed under the SCC1 promoter (left) and expression of Smc5-13myc. Strain: (Y2891). (E) Lack of localization of Smc5-13myc to chromosomes in PSCC1-CDC6 strain. (F) Depletion of Top2 expressed under the CLB2 promoter (left) and expression of Smc5-13myc. Note, this strain arrests at pachynema. Strain: (Y2851). (G) Diminished localization of Smc5-13myc to chromosomes in PCLB2-TOP2 strain. Smc5-13myc foci numbers remained normal in top1-mn, top3-mn, sgs1-mn, rad50S, dmc1Δ, zip1Δ, zip2Δ, zip3Δ, mer3Δ, pch2Δ, fpr3Δ (data not shown). (PDF) [file pgen.1004071.s001.pdf]

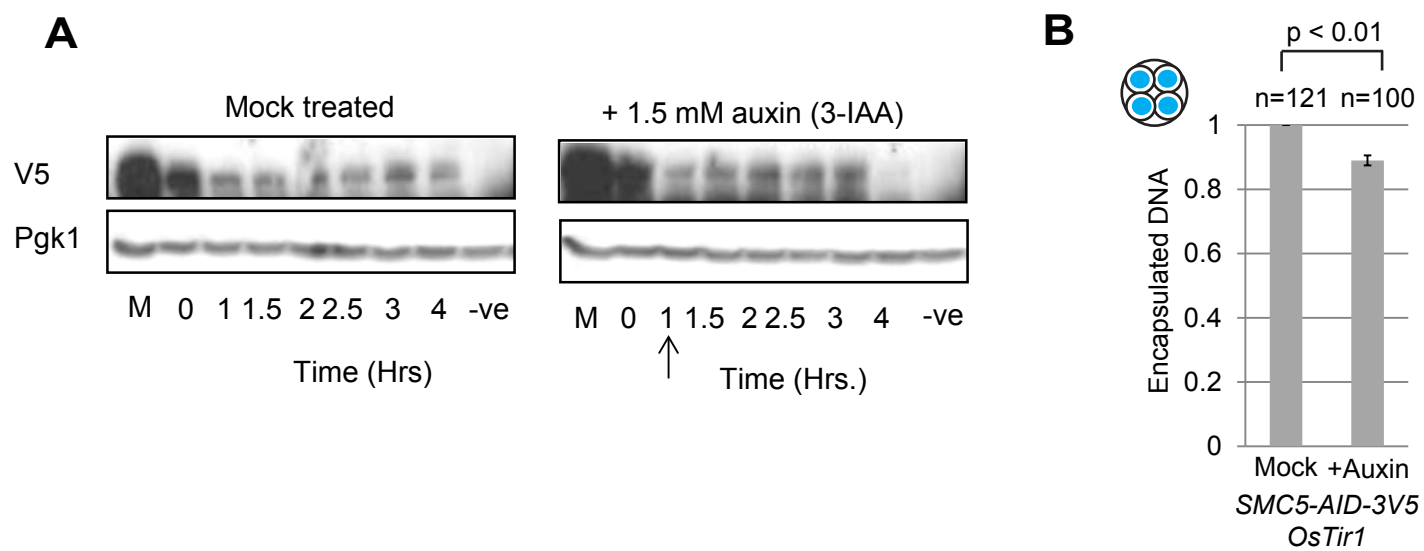

Supplement: Figure S3 — Auxin-induced degradation of Smc5-AID. (A) Western blot analysis of Smc5-AID-V5 after mock treatment or treatment with 1.5 mM auxin at 1 hour after transfer to sporulation medium. Strain: (Y4540). (B) Quantification of DNA encapsulation in Smc5-AID depleted cells. Note that continuous treatment with auxin leads to better depletion and a more severe phenotype, but that the mock-treatment with solvent (NaOH) alone (but not solvent+auxin) causes sporulation defects. (PDF) [file pgen.1004071.s003.pdf]

FIGURE S4

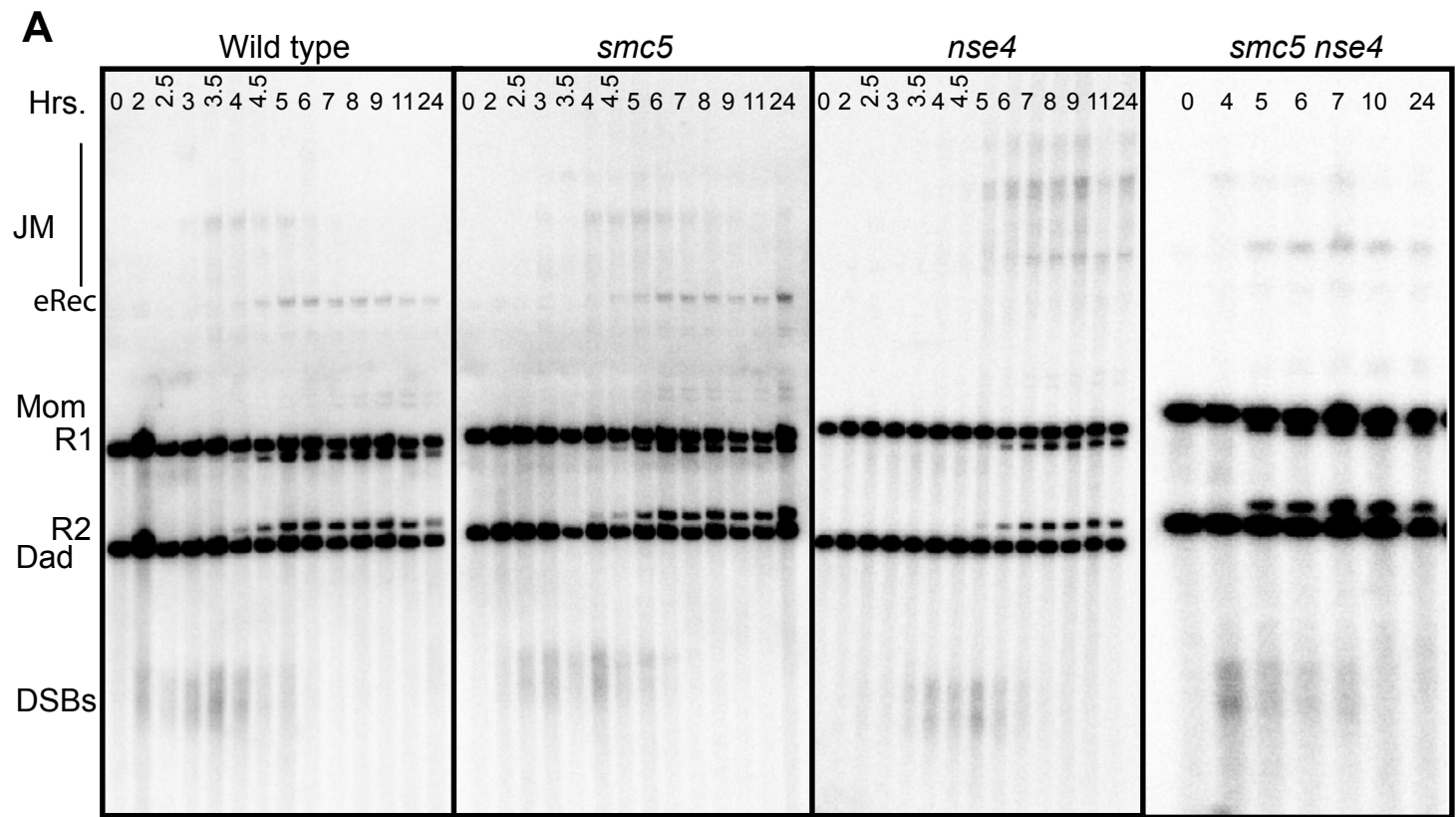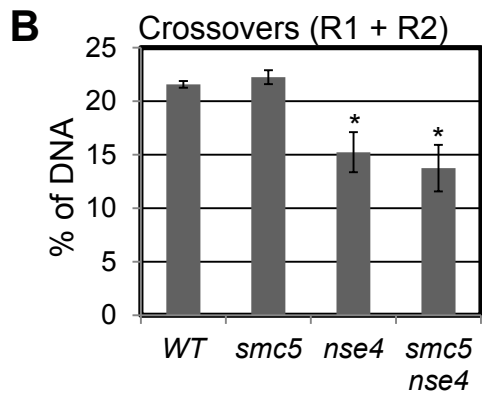

Supplement: Figure S4 — Meiotic recombination and crossing over in the smc5 nse4 mutant is similar to the nse4 single mutant. (A) Example of 1D analysis of crossover recombination. (B) Quantification of crossover levels from three independent diploids (24 hours). Strains: WT (Y2976), smc5 (Y1211), nse4 (Y1212), smc5 nse4 (Y4179). (PDF) [file pgen.1004071.s004.pdf]

**A**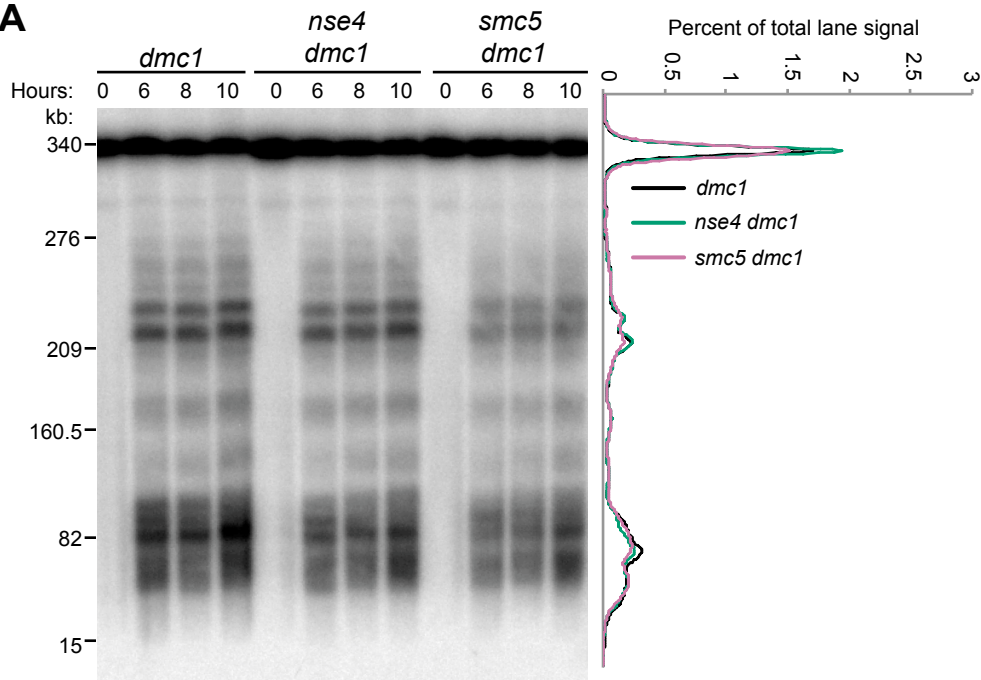**B**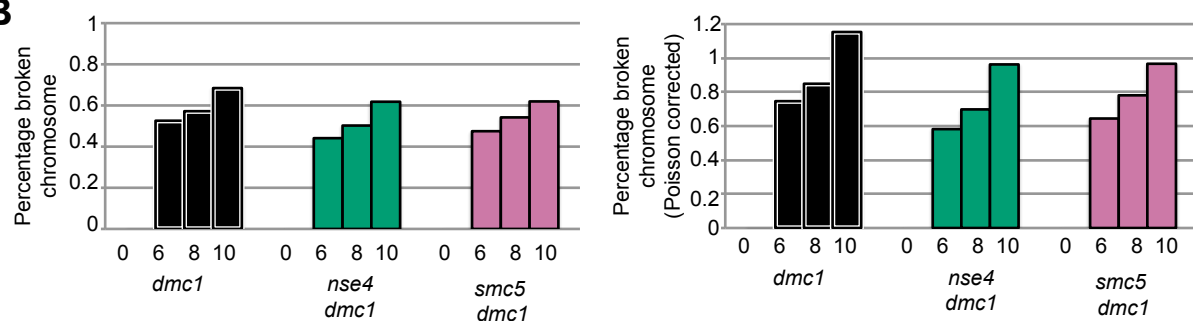**C**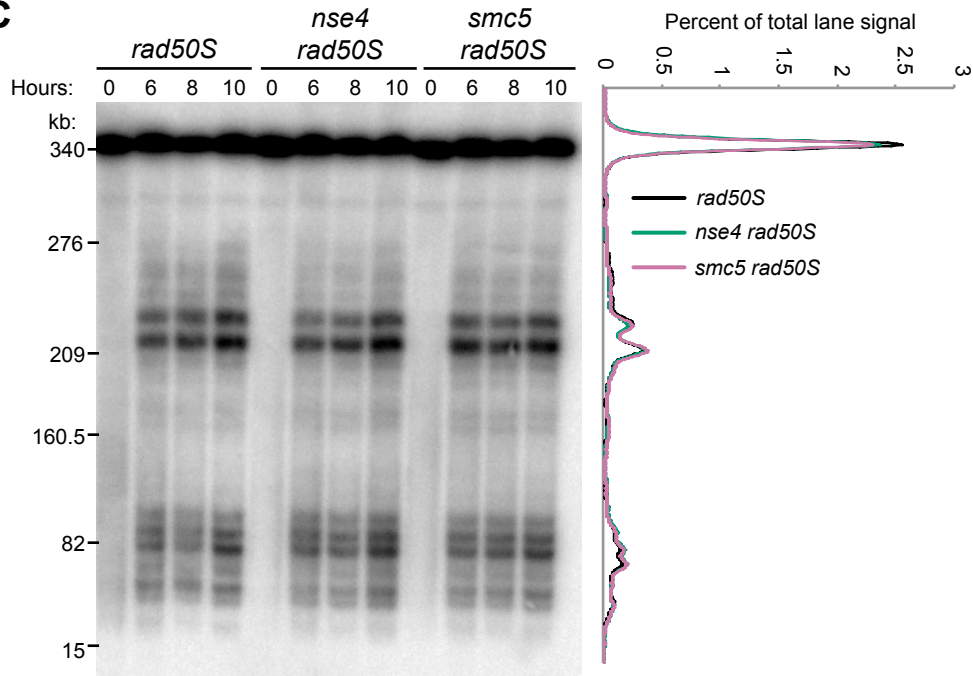**D**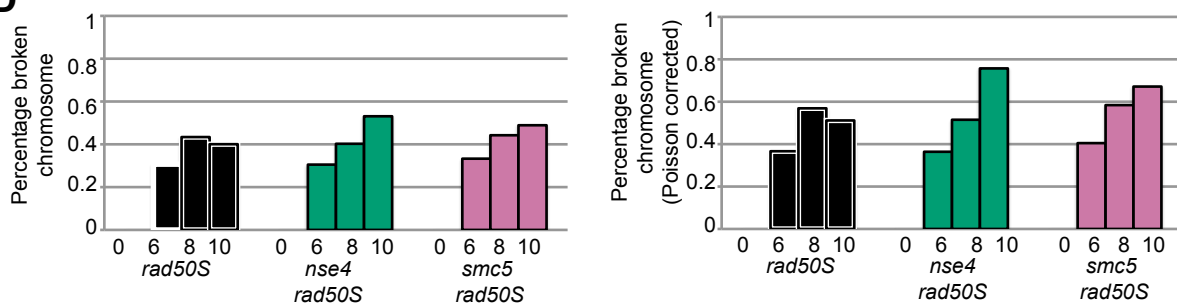

Supplement: Figure S5 — Smc5- or Nse4-depletion does not increase DSB levels in RAD50S or dmc1Δ mutants. (A) Representative CHEF gel followed by Southern blotting using the CHA1 probe (chromosome III, left end) in dmc1Δ strain background. Percentage total lane signal was calculated by smoothing the histogram of signals from 900 bins in each lane. Strains: dmc1Δ (SG492), nse4 dmc1Δ (SG481), and smc5 dmc1Δ (SG478). (B) Quantification of DSBs (non-parentally sized fragments) are presented as raw data (left) or Poisson corrected (right, see materials and methods) for each time point. (C) Representative CHEF gel followed by Southern blotting using the CHA1 probe (chromosome III, left end) in RAD50S strain background. Strains: RAD50S (SG488), nse4 RAD50S (SG484), and smc5 RAD50S (SG491). (D) Quantification of DSBs are presented as raw data (left) or Poisson corrected (right). (PDF) [file pgen.1004071.s005.pdf]

**A**

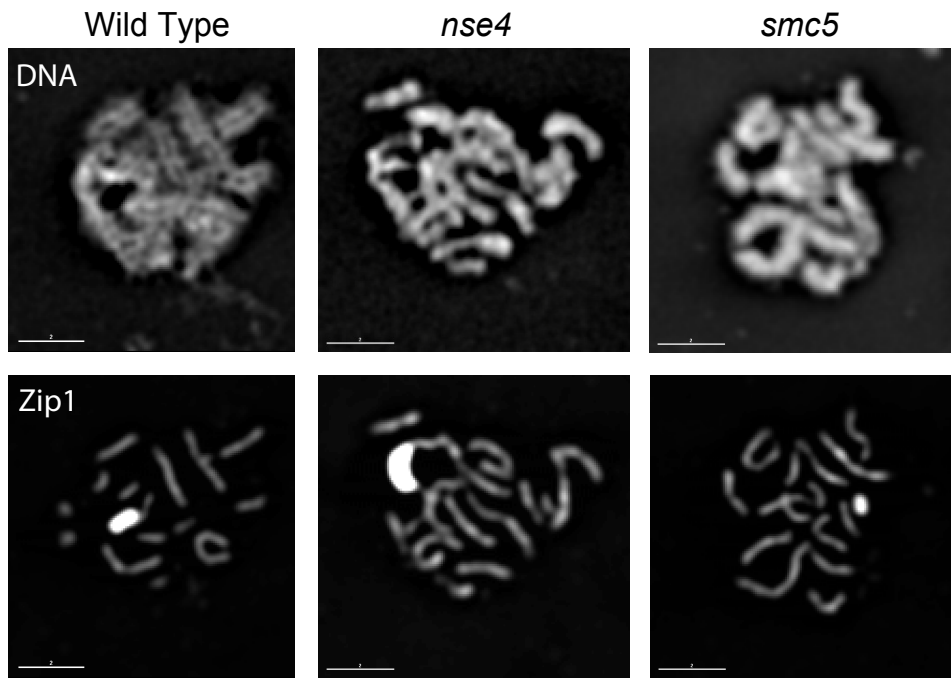

**B**

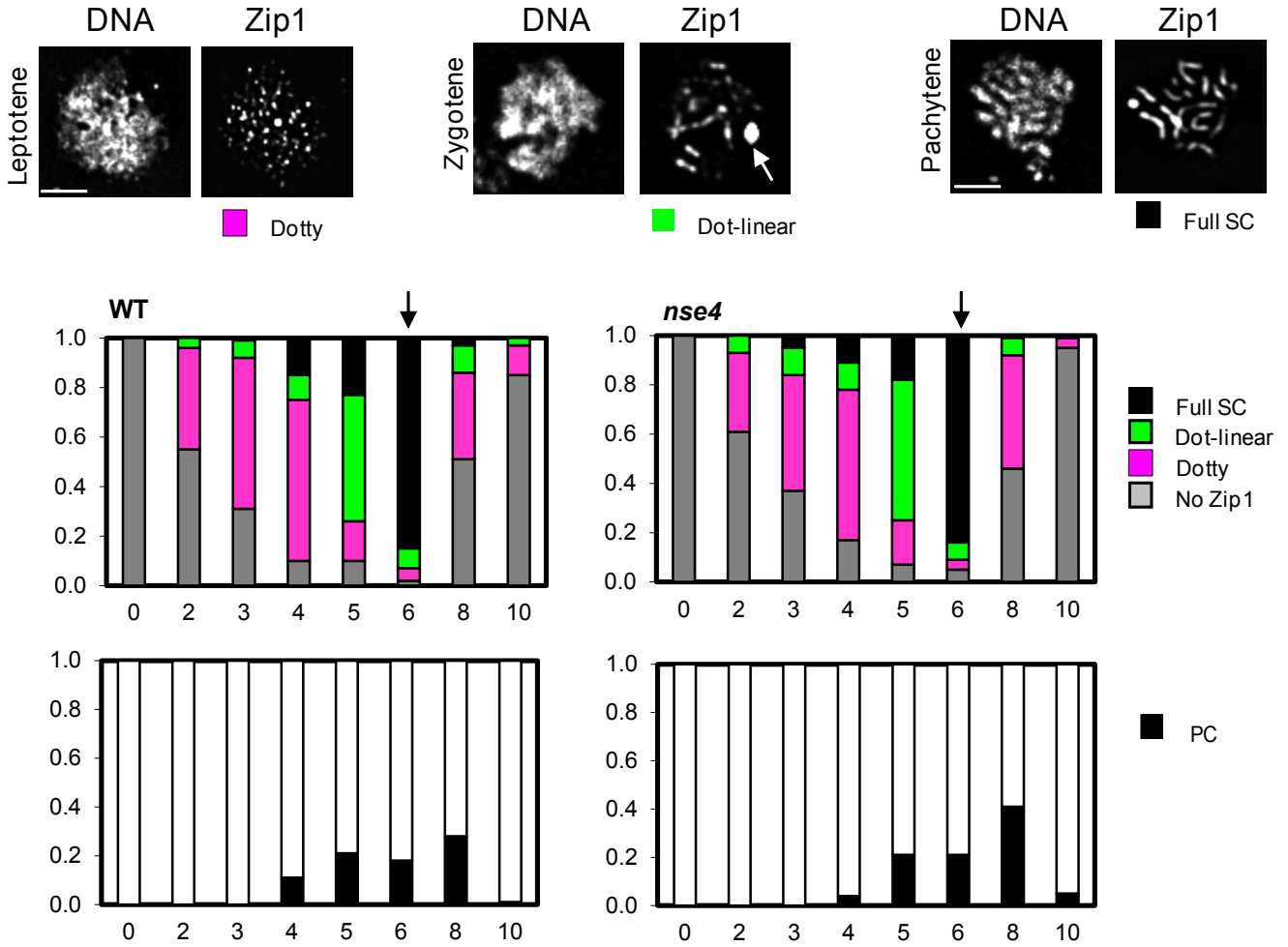

**C**

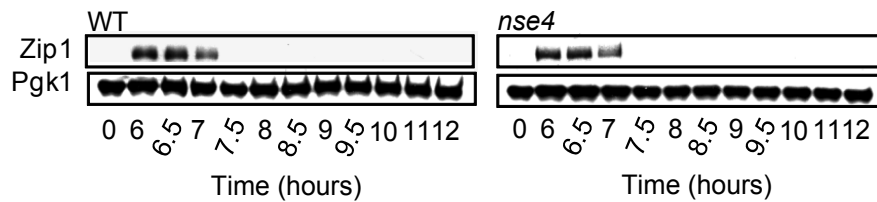

Supplement: Figure S6 — SC formation and disassembly occurs with normal kinetics in the smc5 and nse4 mutants. (A) Examples of Zip1 staining at pachynema in the wild type, nse4 and smc5 mutants. Strains: WT (Y967), smc5 (Y3080) and nse4 (Y2729). (B,C) Kinetics of Zip1 staining patterns and polycomplex formation (PC) in wild type and the nse4 mutant. Left: Examples of Zip1 behaviour as ‘dotty’, ‘dot-linear’ and ‘linear’ staining, representative of leptonema, zygonema, and pachynema, respectively in nuclei from the nse4 mutant (these are similar to those seen in wild type). The arrow indicates an aggregate of Zip1, likely a polycomplex (PC). Bars, 2 µm. Right: Proportion of nuclei with no Zip1, dotty, dot-linear, or fully linear Zip1 staining (upper panel) and the proportion containing a PC (lower panel). At least 100 nuclei were inspected for each time point. We chose a time course where spindle formation kinetics indicated similar synchrony in the two strains to allow direct comparison (not shown). The arrow denotes the time at which cells were released from prophase I arrest by induction of NDT80 expression (NDT80-IN) allowing SC disassembly and Zip1 degradation (C) to be followed. (PDF) [file pgen.1004071.s006.pdf]

**A**

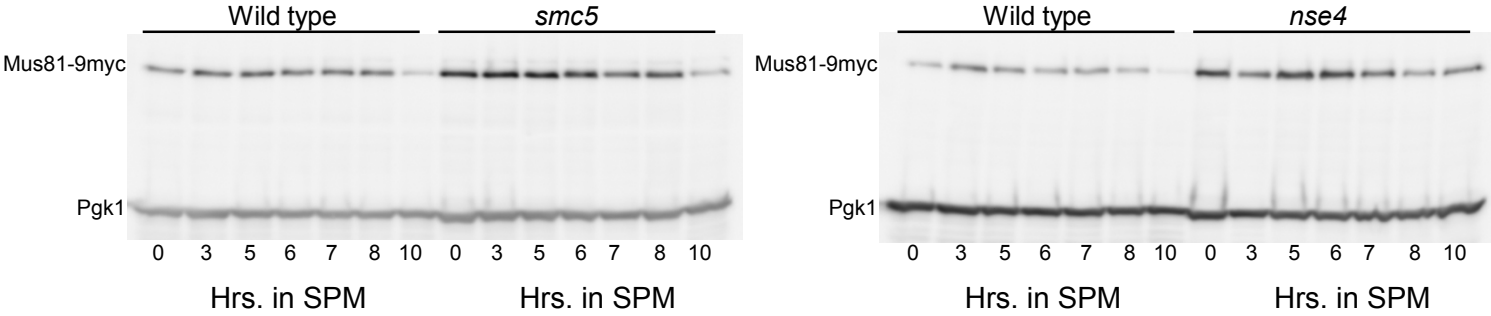

**B**

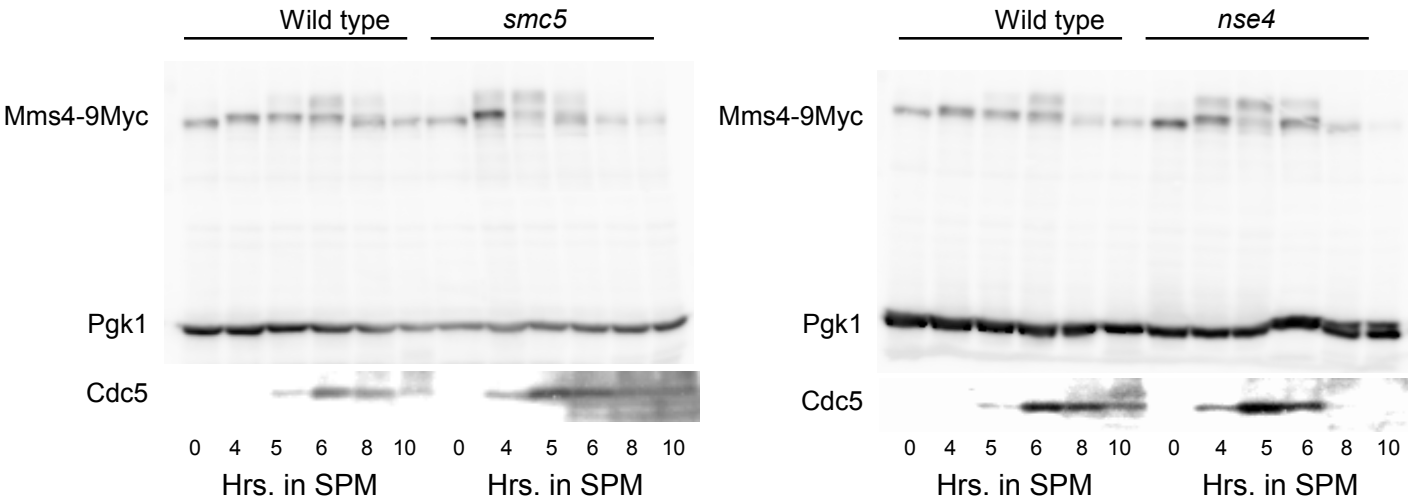

**C**

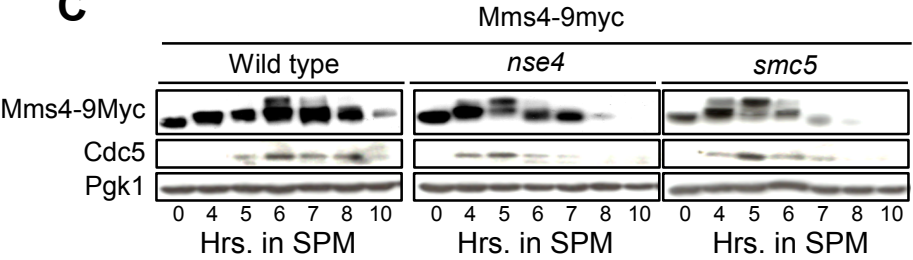

Supplement: Figure S7 — Steady-state levels and hyperphosphorylation of Mus81-9Myc and Mms4-9myc are not decreased in the Smc5-and Nse4-depleted strains. (A,B) Western blot of Mus81-9myc and Mms4-9myc. Loading factor Pgk1 was analysed on the same Western blot. Strains: WT (Y3618- Mus81-9myc, Y3683- Mms4-9myc), smc5 (Y3621- Mus81-9myc, Y3689- Mms4-9myc) and nse4 (Y3624-Mus81-9myc, Y3686- Mms4-9myc). (C) Mms4-9myc hyperphosphorylation occurs concomitantly with Cdc5 expression in wild type as well as the smc5 and nse4 strains. Pgk1 was used as loading factor. (PDF) [file pgen.1004071.s007.pdf]

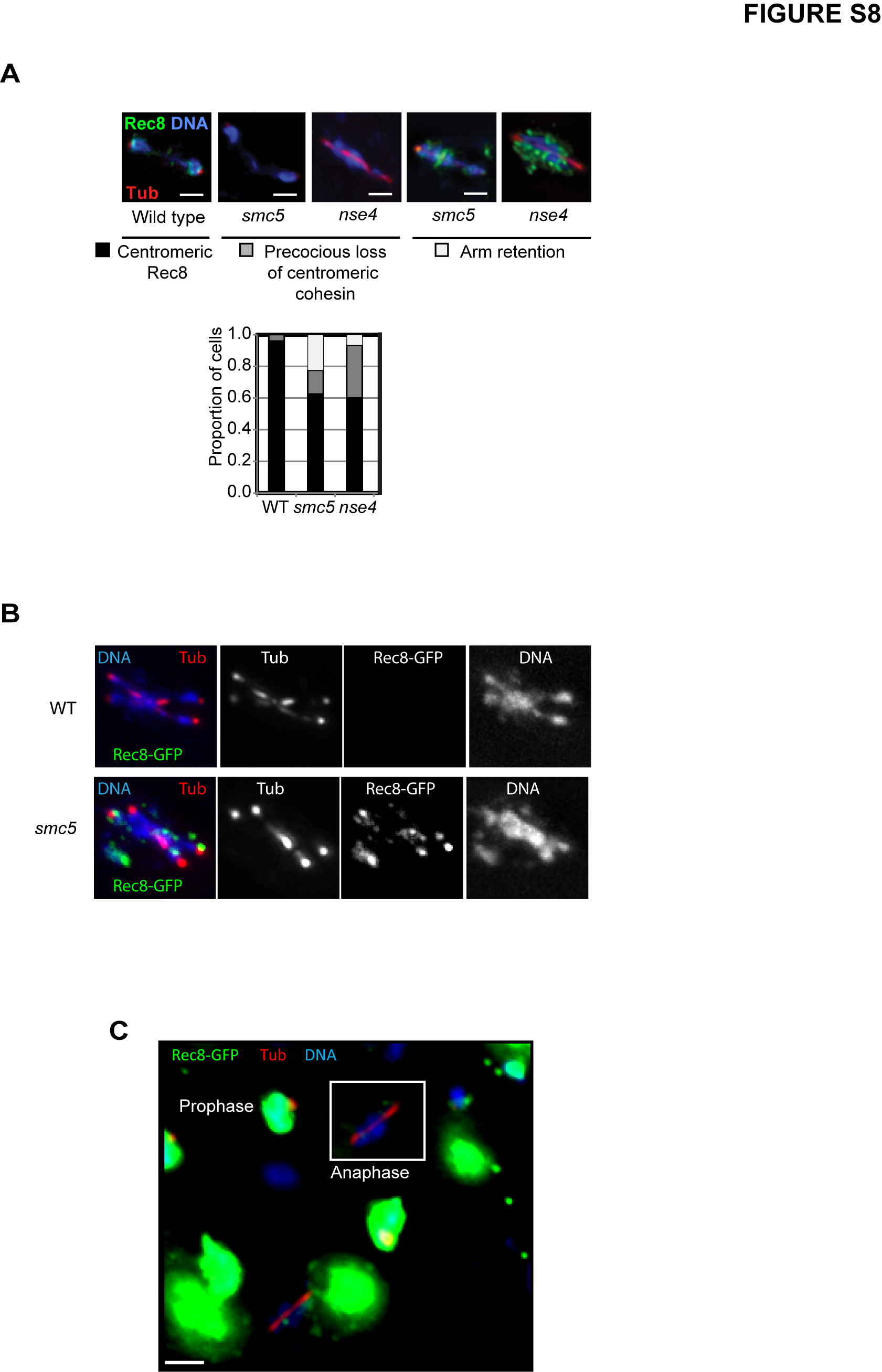

Supplement: Figure S8 — (A) Immunostaining of fixed, semi-spread nuclei at anaphase I. Examples of anaphase I nuclei with associated Rec8-GFP along arms (‘arm retention’) as well as precocious loss of centromeric cohesin. Quantification is shown below. Anaphase I nuclei were staged by length; imaging with Pds1-tdTomato showed that all anaphase I spindles >4 µm were at anaphase I in wild type as well as the two mutants. (B) Representative images of Rec8-GFP of anaphase II nuclei in the wild type and smc5 mutant. (C) Overexposure of the FITC (Rec8-GFP) channel to illustrate that the centromeric Rec8 is indeed not detected at anaphase I in smc5 and nse4 mutants. Box illustrates an anaphase I spindle (>4 µm). Overexposed GFP signals are from prophase I nuclei. (TIF) [file pgen.1004071.s008.tif]
