# Supplementary material for: Smc5/6 Coordinates Formation and Resolution of Joint Molecules with Chromosome Morphology to Ensure Meiotic Divisions
Source: PLoS Genet. 2013 Dec 26;9(12):e1004071. doi: 10.1371/journal.pgen.1004071 (PMC3873251; doi:10.1371/journal.pgen.1004071)
Supplement: Table S1 — List of strains used in this study. Individual strains used for the experiments are listed in the relevant figure legend. (DOCX) [file pgen.1004071.s017.docx]

Table S1.

| **Strain^1^** | **Genotype** |
| --- | --- |
| Y940 | *MAT α HIS4 CY2 arg4(Eco47III-hpa1)  MAT* ***a*** *his4Δ (Sal1-Cla1)::ura3Δ(Sma1-Eco47III)-arg4-EcPal(1691) cyh2-z arg4(Eco47III-hpa1)*  *leu2-R lys2Δ0 ura3 ho::LYS2*  *leu2-RV::URA3-(Sma1-Eco47III)-[ARG4cloned] lys2Δ0 ura3 ho::LYS2* |
| Y941 | *MAT α HIS4 CY2 arg4(Eco47III-hpa1)  MAT* ***a*** *his4Δ (Sal1-Cla1)::ura3Δ(Sma1-Eco47III)-arg4-EcPal(1691) cyh2-z arg4(Eco47III-hpa1)*  *leu2-R lys2Δ0 ura3 ho::LYS2 KANMX6-pSCC1-3HA-SMC5*  *leu2-RV::URA3-(Sma1-Eco47III)-[ARG4cloned] lys2Δ0 ura3 ho::LYS2 KANMX6-pSCC1-3HA-SMC5* |
| Y942 | *MAT α HIS4 CY2 arg4(Eco47III-hpa1)  MAT* ***a*** *his4Δ (Sal1-Cla1)::ura3Δ(Sma1-Eco47III)-arg4-EcPal(1691) cyh2-z arg4(Eco47III-hpa1)*  *leu2-R lys2Δ0 ura3 ho::LYS2 KANMX6-pSCC1-3HA-NSE4*  *leu2-RV::URA3-(Sma1-Eco47III)-[ARG4cloned] lys2Δ0 ura3 ho::LYS2 KANMX6-pSCC1-3HA-NSE4* |
| Y957 | *MAT α, his3::hisG, leu2::hisG, trp1::hisG, lys2, ura3,* *ho::LYS2* |
| Y958 | *MAT* ***a,*** *his3::hisG, leu2::hisG, trp1::hisG, lys2, ura3,* *ho::LYS2* |
| Y967 | *MAT α his3::hisG leu2::hisG trp1::hisG lys2 ura3 ho::LYS2*  *MAT* ***a*** *his3::hisG leu2::hisG trp1::hisG lys2 ura3 ho::LYS2*  *ER-GAL4TF::URA3 pGAL1-NDT80::TRP1*  *ER-GAL4TF::URA3 pGAL1-NDT80::TRP1* |
| Y1211 | *MAT α HIS4::LEU2-(BamHI;+ori) leu2::hisG ura3∆(sma-pst) ho::hisG*  *MAT* ***a*** *his4-X::LEU2-(NgoMIV;+ori)--URA3 leu2::hisG ura3∆(sma-pst) ho::hisG*  *KANMX6-pCLB2-3HA-SMC5*  *KANMX6-pCLB2-3HA-SMC5* |
| Y1212 | *MAT α HIS4::LEU2-(BamHI;+ori) leu2::hisG ura3∆(sma-pst) ho::hisG*  *MAT* ***a*** *his4-X::LEU2-(NgoMIV;+ori)--URA3 leu2::hisG ura3∆(sma-pst) ho::hisG*  *KANMX6-pCLB2-3HA-NSE4*  *KANMX6-pCLB2-3HA-NSE4* |
| Y1381 | *MAT α his3::hisG leu2::hisG trp1::hisG lys2 ura3 ho::LYS2*  *MAT* ***a*** *his3::hisG leu2::hisG trp1::hisG lys2 ura3 ho::LYS2* |
| Y1435 | *MAT α his3::hisG leu2::hisG trp1::hisG lys2 ura3 ho::LYS2*  *MAT* ***a*** *his3::hisG leu2::hisG trp1::hisG lys2 ura3 ho::LYS2*  *ZIP3-GFP*::*URA3*  *ZIP3-GFP::URA3* |
| Y1485 | *MAT α his3::hisG leu2::hisG trp1::hisG lys2 ura3 ho::LYS2*  *MAT* ***a*** *his3::hisG leu2::hisG trp1::hisG lys2 ura3 ho::LYS2*  *PDS1-MYC18::TRP1 REC8-HA3::URA3*  *PDS1-MYC18::TRP1 REC8-HA3::URA3* |
| Y2045 | *MAT α his3::hisG leu2::hisG trp1::hisG lys2 ura3 ho::LYS2*  *MAT* ***a*** *his3::hisG leu2::hisG trp1::hisG lys2 ura3 ho::LYS2*  *NSE4-13MYC::KANMX6*  *NSE4-13MYC::KANMX6* |
| Y2404 | *MAT α his3::hisG leu2::hisG trp1::hisG lys2 ura3 ho::LYS2*  *MAT* ***a*** *his3::hisG leu2::hisG trp1::hisG lys2 ura3 ho::LYS2*  *rec8*∆*::KanMX4*  *rec8*∆*::KanMX4* |
| Y2489 | *MAT α his3::hisG leu2::hisG trp1::hisG lys2 ura3 ho::LYS2*  *MAT* ***a*** *his3::hisG leu2::hisG trp1::hisG lys2 ura3 ho::LYS2*  *hop1Δ::KanMX4*  *hop1Δ::KanMX4* |
| Y2572 | *MAT α his3::hisG leu2::hisG trp1::hisG lys2 ura3 ho::LYS2*  *MAT* ***a*** *his3::hisG leu2::hisG trp1::hisG lys2 ura3 ho::LYS2*  *REC8-GFP::URA3 PDS1-tdToMATo::KlTRP1 CNM67-3mCherry::NATMX4*  *REC8-GFP::URA3 PDS1-tdToMATo::KlTRP1 CNM67-3mCherry::NATMX4* |
| Y2671 | *MAT α his3::hisG leu2::hisG trp1::hisG lys2 ura3 ho::LYS2*  *MAT* ***a*** *his3::hisG leu2::hisG trp1::hisG lys2 ura3 ho::LYS2*  *KANMX6-pCLB2-3HA-SMC5 REC8-GFP::URA3*  *KANMX6-pCLB2-3HA-SMC5 REC8-GFP::URA3* |
| Y2673 | *MAT α his3::hisG leu2::hisG trp1::hisG lys2 ura3 ho::LYS2*  *MAT* ***a*** *his3::hisG leu2::hisG trp1::hisG lys2 ura3 ho::LYS2*  *KANMX6-pCLB2-3HA-SMC5 REC8-GFP::URA3 PDS1-tdToMATo::KlTRP1 CNM67-3mCherry-NATMX4*  *KANMX6-pCLB2-3HA-SMC5 REC8-GFP::URA3 PDS1-tdToMATo::KlTRP1 CNM67-3mCherry-NATMX4* |
| Y2689 | *MAT α his3::hisG leu2::hisG trp1::hisG lys2 ura3 ho::LYS2*  *MAT* ***a*** *his3::hisG leu2::hisG trp1::hisG lys2 ura3 ho::LYS2*  *REC8-GFP::URA3*  *REC8-GFP::URA3* |
| Y2704 | *MAT α his3::hisG leu2::hisG trp1::hisG lys2 ura3 ho::LYS2*  *MAT* ***a*** *his3::hisG leu2::hisG trp1::hisG lys2 ura3 ho::LYS2*  *HPHMX4-pCLB2-3HA-NSE4*  *HPHMX4-pCLB2-3HA-NSE4* |
| Y2705 | *MAT α his3::hisG leu2::hisG trp1::hisG lys2 ura3 ho::LYS2*  *MAT* ***a*** *his3::hisG leu2::hisG trp1::hisG lys2 ura3 ho::LYS2*  *HPHMX4-pCLB2-3HA-SMC5*  *HPHMX4-pCLB2-3HA-SMC5* |
| Y2708 | *MAT α CEN5 his3::hisG leu2::hisG trp1::hisG lys2 ura3 ho::LYS2*  *MAT* ***a*** *CEN5::tetO_224_::HIS3 his3::hisG leu2::pURA3::TetR::GFP::LEU2 trp1::hisG lys2 ura3 ho::LYS2* |
| Y2709 | *MAT α CEN5 his3::hisG leu2::hisG trp1::hisG lys2 ura3 ho::LYS2*  *MAT* ***a*** *CEN5::tetO_224_::HIS3 his3::hisG leu2::pURA3::TetR::GFP::LEU2 trp1::hisG lys2 ura3 ho::LYS2*  *KANMX6-pCLB2-3HA-SMC5*  *KANMX6-pCLB2-3HA-SMC5* |
| Y2729 | *MAT α his3::hisG leu2::hisG trp1::hisG lys2 ura3 ho::LYS2*  *MAT* ***a*** *his3::hisG leu2::hisG trp1::hisG lys2 ura3 ho::LYS2*  *HPHMX4-pCLB2-3HA-NSE4 ER-GAL4TF::URA3 pGAL1-NDT80::TRP1*  *HPHMX4-pCLB2-3HA-NSE4 ER-GAL4TF::URA3 pGAL1-NDT80::TRP1* |
| Y2751 | *MAT α his3::hisG leu2::hisG trp1::hisG lys2 ura3 ho::LYS2*  *MAT* ***a*** *his3::hisG leu2::hisG trp1::hisG lys2 ura3 ho::LYS2*  *KANMX6-pCLB2-3HA-SMC5 PDS1-MYC18::TRP1 REC8-HA3::URA3*  *KANMX6-pCLB2-3HA-SMC5 PDS1-MYC18::TRP1 REC8-HA3::URA3* |
| Y2816 | *MAT α leu2::hisG trp1::hisG ura3 ho::LYS2*  *MAT* ***a*** *leu2::hisG trp1::hisG ura3 ho::LYS2*  *REC8-HA3::URA3 spo11Δ::URA3 spo13Δ::hisG*  *REC8-HA3::URA3 spo11Δ::URA3 spo13Δ::hisG* |
| Y2819 | *MAT α leu2::hisG trp1::hisG ura3 ho::LYS2*  *MAT* ***a*** *leu2::hisG trp1::hisG ura3 ho::LYS2*  *SMC5-13MYC::KANMX6 NDC10-6HA::HIS3MX6*  *SMC5-13MYC::KANMX6 NDC10-6HA::HIS3MX6* |
| Y2824 | *MAT α his3::hisG leu2::hisG trp1::hisG lys2 ura3 ho::LYS2*  *MAT* ***a*** *his3::hisG leu2::hisG trp1::hisG lys2 ura3 ho::LYS2*  *SMC5-13MYC::KANMX6 ER-GAL4TF::URA3 pGAL1-NDT80::TRP1*  *SMC5-13MYC::KANMX6 ER-GAL4TF::URA3 pGAL1-NDT80::TRP1* |
| Y2826 | *MAT α his3::hisG leu2::hisG trp1::hisG lys2 ura3 ho::LYS2*  *MAT* ***a*** *his3::hisG leu2::hisG trp1::hisG lys2 ura3 ho::LYS2*  *NSE4-CBP-TEV-ProtA::KlURA3 ER-GAL4TF::URA3 pGAL1-NDT80::TRP1*  *NSE4-CBP-TEV-ProtA::KlURA3 ER-GAL4TF::URA3 pGAL1-NDT80::TRP1* |
| Y2836 | *MAT α leu2::hisG trp1::hisG ura3 ho::LYS2*  *MAT* ***a*** *leu2::hisG trp1::hisG ura3 ho::LYS2*  *SMC5-13MYC::KANMX6 spo11-Y135F-HA::URA3*  *SMC5-13MYC::KANMX6 spo11-Y135F-HA::URA3* |
| Y2837 | *MAT α his3::hisG leu2::hisG trp1::hisG lys2 ura3 ho::LYS2*  *MAT* ***a*** *his3::hisG leu2::hisG trp1::hisG lys2 ura3 ho::LYS2*  *SMC5-13MYC::KANMX6 rec8∆::KanMX4*  *SMC5-13MYC::KANMX6 rec8∆::KanMX4* |
| Y2846 | *MAT α his3::hisG leu2::hisG trp1::hisG lys2 ura3 ho::LYS2*  *MAT* ***a*** *his3::hisG leu2::hisG trp1::hisG lys2 ura3 ho::LYS2*  *KANMX6-pCLB2-3HA-SMC5 REC8-HA3::URA3 spo11Δ::URA3 spo13Δ::hisG*  *KANMX6-pCLB2-3HA-SMC5 REC8-HA3::URA3 spo11Δ::URA3 spo13Δ::hisG* |
| Y2848 | *MAT α his3::hisG leu2::hisG trp1::hisG lys2 ura3 ho::LYS2*  *MAT* ***a*** *his3::hisG leu2::hisG trp1::hisG lys2 ura3 ho::LYS2*  *KANMX6-pCLB2-3HA-NSE4 REC8-HA3::URA3 spo11Δ::URA3 spo13Δ::hisG*  *KANMX6-pCLB2-3HA-NSE4 REC8-HA3::URA3 spo11Δ::URA3 spo13Δ::hisG* |
| Y2851 | *MAT α his3::hisG leu2::hisG trp1::hisG lys2 ura3 ho::LYS2*  *MAT* ***a*** *his3::hisG leu2::hisG trp1::hisG lys2 ura3 ho::LYS2*  *SMC5-13MYC::KANMX6 KANMX6-pCLB2-3HA-TOP2*  *SMC5-13MYC::KANMX6 KANMX6-pCLB2-3HA-TOP2* |
| Y2855 | *MAT α his3::hisG leu2::hisG trp1::hisG lys2 ura3 ho::LYS2*  *MAT* ***a*** *his3::hisG leu2::hisG trp1::hisG lys2 ura3 ho::LYS2*  *HPHMX4-pCLB2-3HA-NSE4 rec8Δ::KanMX4*  *HPHMX4-pCLB2-3HA-NSE4 rec8Δ::KanMX4* |
| Y2856 | *MAT α his3::hisG leu2::hisG trp1::hisG lys2 ura3 ho::LYS2*  *MAT* ***a*** *his3::hisG leu2::hisG trp1::hisG lys2 ura3 ho::LYS2*  *HPHMX4-pCLB2-3HA-SMC5 rec8*∆*::KanMX4*  *HPHMX4-pCLB2-3HA-SMC5 rec8*∆*::KanMX4* |
| Y2891 | *MAT α his3::hisG leu2::hisG trp1::hisG lys2 ura3 ho::LYS2*  *MAT* ***a*** *his3::hisG leu2::hisG trp1::hisG lys2 ura3 ho::LYS2*  *SMC5-13MYC::KANMX6 KANMX6-pSCC12-3HA-CDC6*  *SMC5-13MYC::KANMX6 KANMX6-pSCC1-3HA-CDC6* |
| Y2976 | *MAT α HIS4::LEU2-(BamHI;+ori) leu2::hisG ura3∆(sma-pst) ho::hisG*  *MAT* ***a*** *his4-X::LEU2-(NgoMIV;+ori)--URA3 leu2::hisG ura3∆(sma-pst) ho::hisG* |
| Y3025 | *MAT α HIS4::LEU2-(BamHI;+ori) leu2::hisG ura3∆(sma-pst) ho::hisG*  *MAT* ***a*** *his4-X::LEU2-(NgoMIV;+ori)--URA3 leu2::hisG ura3∆(sma-pst) ho::hisG*  *ndt80∆::KANMX4*  *ndt80∆::KANMX4* |
| Y3041 | *MAT α his3::hisG leu2::hisG trp1::hisG lys2 ura3 ho::LYS2*  *MAT* ***a*** *his3::hisG leu2::hisG trp1::hisG lys2 ura3 ho::LYS2*  *REC8-GFP::URA3 PDS1-tdTomato::KlTRP1 CNM67-3mCherry::NATMX4, KANMX6-PCLB2-3HA-SGS1*  *REC8-GFP::URA3 PDS1-tdTomato::KlTRP1 CNM67-3mCherry::NATMX4, KANMX6-PCLB2-3HA-SGS1* |
| Y3047 | *MAT α his3::hisG leu2::hisG trp1::hisG lys2 ura3 ho::LYS2*  *MAT* ***a*** *his3::hisG leu2::hisG trp1::hisG lys2 ura3 ho::LYS2*    *REC8-GFP::URA3 PDS1-tdToMATo::KlTRP1 CNM67-3mCherry::NATMX4 KANMX6-PCLB2-3HA-NSE4*  *REC8-GFP::URA3 PDS1-tdToMATo::KlTRP1 CNM67-3mCherry::NATMX4 KANMX6-PCLB2-3HA-NSE4* |
| Y3067 | *MAT α his3::hisG leu2::hisG trp1::hisG lys2 ura3 ho::LYS2*  *MAT* ***a*** *his3::hisG leu2::hisG trp1::hisG lys2 ura3 ho::LYS2*    *TOP2-eGFP::KANMX4 SMC5-13MYC::KANMX6*  *TOP2-eGFP::KANMX4 SMC5-13MYC::KANMX6* |
| Y3080 | *MAT α his3::hisG leu2::hisG trp1::hisG lys2 ura3 ho::LYS2*  *MAT* ***a*** *his3::hisG leu2::hisG trp1::hisG lys2 ura3 ho::LYS2*  *HPHMX4-pCLB2-3HA-SMC5 ER-GAL4TF::URA3 pGAL1-NDT80::TRP1*  *HPHMX4-pCLB2-3HA-SMC5 ER-GAL4TF::URA3 pGAL1-NDT80::TRP1* |
| Y3135 | *MAT α his3::hisG leu2::hisG trp1::hisG lys2 ura3 ho::LYS2*  *MAT* ***a*** *his3::hisG leu2::hisG trp1::hisG lys2 ura3 ho::LYS2*  *MUS81-9MYC::KlTRP1 HPHMX4-pCLB2-3HA-SMC5*  *MUS81-9MYC::KlTRP1 HPHMX4-pCLB2-3HA-SMC5* |
| Y3137 | *MAT α his3::hisG leu2::hisG trp1::hisG lys2 ura3 ho::LYS2*  *MAT* ***a*** *his3::hisG leu2::hisG trp1::hisG lys2 ura3 ho::LYS2*  *MUS81-9MYC::KlTRP1*  *MUS81-9MYC::KlTRP1* |
| Y3144 | *MAT α his3::hisG leu2::hisG trp1::hisG lys2 ura3 ho::LYS2*  *MAT* ***a*** *his3::hisG leu2::hisG trp1::hisG lys2 ura3 ho::LYS2*  *MUS81-9MYC::KlTRP1 HPHMX4-pCLB2-3HA-NSE4*  *MUS81-9MYC::KlTRP1 HPHMX4-pCLB2-3HA-NSE4* |
| Y3147 | *MAT α his3::hisG leu2::hisG trp1::hisG lys2 ura3 ho::LYS2*  *MAT* ***a*** *his3::hisG leu2::hisG trp1::hisG lys2 ura3 ho::LYS2*  *spo11-Y135F-HA::URA3 REC8-GFP::URA3 Ctf19-13MYC::KANMX6*  *spo11-Y135F-HA::URA3 REC8-GFP::URA3 Ctf19-13MYC::KANMX6* |
| Y3150 | *MAT α his3::hisG leu2::hisG trp1::hisG lys2 ura3 ho::LYS2*  *MAT* ***a*** *his3::hisG leu2::hisG trp1::hisG lys2 ura3 ho::LYS2*  *spo11-Y135F-HA::URA3 REC8-GFP::URA3 Ctf19-13MYC::KANMX6 HPHMX4-PCLB2-3HA-SMC5*  *spo11-Y135F-HA::URA3 REC8-GFP::URA3 Ctf19-13MYC::KANMX6 HPHMX4-PCLB2-3HA-SMC5* |
| Y3153 | *MAT α his3::hisG leu2::hisG trp1::hisG lys2 ura3 ho::LYS2*  *MAT* ***a*** *his3::hisG leu2::hisG trp1::hisG lys2 ura3 ho::LYS2*  *spo11-Y135F-HA::URA3 REC8-GFP::URA3 Ctf19-13MYC::KANMX6 HPHMX4-PCLB2-3HA-NSE4*  *spo11-Y135F-HA::URA3 REC8-GFP::URA3 Ctf19-13MYC::KANMX6 HPHMX4-PCLB2-3HA-NSE4* |
| Y3185 | *MAT α his3::hisG leu2::hisG trp1::hisG lys2 ura3 ho::LYS2*  *MAT* ***a*** *his3::hisG leu2::hisG trp1::hisG lys2 ura3 ho::LYS2*  *HPHMX4-pCLB2-3HA-NSE4 KANMX6-pCLB2-3HA-SMC5*  *HPHMX4-pCLB2-3HA-NSE4 KANMX6-pCLB2-3HA-SMC5* |
| Y3237 | *MAT α his3::hisG leu2::hisG trp1::hisG lys2 ura3 ho::LYS2*  *MAT* ***a*** *his3::hisG leu2::hisG trp1::hisG lys2 ura3 ho::LYS2*  *rec8∆::KANMX4-Rec8-PK-TEV287::LEU2 GAL-NLS-myc-9-TEV-protease-NLS-NLS::TRP1*  *rec8∆::KANMX4-Rec8-PK-TEV287::LEU2 GAL-NLS-myc-9-TEV-protease-NLS-NLS::TRP1*  *HPHMX4-pCLB2-3HA-SMC5 ER-GAL4TF::URA3 pGAL1-NDT80::TRP1*  *HPHMX4-pCLB2-3HA-SMC5 ER-GAL4TF::URA3 pGAL1-NDT80::TRP1* |
| Y3240 | *MAT α his3::hisG leu2::hisG trp1::hisG lys2 ura3 ho::LYS2*  *MAT* ***a*** *his3::hisG leu2::hisG trp1::hisG lys2 ura3 ho::LYS2*  *rec8*∆*::KANMX4-Rec8-PK-TEV287::LEU2 GAL-NLS-myc-9-TEV-protease-NLS-NLS::TRP1*  *rec8*∆*::KANMX4-Rec8-PK-TEV287::LEU2 GAL-NLS-myc-9-TEV-protease-NLS-NLS::TRP1*  *HPHMX4-pCLB2-3HA-NSE4 ER-GAL4TF::URA3 pGAL1-NDT80::TRP1*  *HPHMX4-pCLB2-3HA-NSE4 ER-GAL4TF::URA3 pGAL1-NDT80::TRP1* |
| Y3252 | *MAT α his3::hisG leu2::hisG trp1::hisG lys2 ura3 ho::LYS2*  *MAT* ***a*** *his3::hisG leu2::hisG trp1::hisG lys2 ura3 ho::LYS2*  *HPHMX4-pCLB2-3HA-SMC5-IAA17::KANMX6 OsTIR1-9Myc::URA3*  *HPHMX4-pCLB2-3HA-SMC5-IAA17::KANMX6 OsTIR1-9Myc::URA3* |
| Y3258 | *MAT α his3::hisG leu2::hisG trp1::hisG lys2 ura3 ho::LYS2*  *MAT* ***a*** *his3::hisG leu2::hisG trp1::hisG lys2 ura3 ho::LYS2*  *rec8*∆*::KANMX4-Rec8-PK-TEV287::LEU2*  *rec8*∆*::KANMX4-Rec8-PK-TEV287::LEU2*  *HPHMX4-pCLB2-3HA-NSE4 ER-GAL4TF::URA3 pGAL1-NDT80::TRP1*  *HPHMX4-pCLB2-3HA-NSE4 ER-GAL4TF::URA3 pGAL1-NDT80::TRP1* |
| Y3261 | *MAT α his3::hisG leu2::hisG trp1::hisG lys2 ura3 ho::LYS2*  *MAT* ***a*** *his3::hisG leu2::hisG trp1::hisG lys2 ura3 ho::LYS2*  *rec8*∆*::KANMX4-Rec8-PK-TEV287::LEU2*  *rec8*∆*::KANMX4-Rec8-PK-TEV287::LEU2*  *HPHMX4-pCLB2-3HA-SMC5 ER-GAL4TF::URA3 pGAL1-NDT80::TRP1*  *HPHMX4-pCLB2-3HA-SMC5 ER-GAL4TF::URA3 pGAL1-NDT80::TRP1* |
| Y3264 | *MAT α his3::hisG leu2::hisG trp1::hisG lys2 ura3 ho::LYS2*  *MAT* ***a*** *his3::hisG leu2::hisG trp1::hisG lys2 ura3 ho::LYS2*  *rec8*∆*::KANMX4-Rec8-PK-TEV287::LEU2 ER-GAL4TF::URA3 pGAL1-NDT80::TRP1*  *rec8*∆*::KANMX4-Rec8-PK-TEV287::LEU2 ER-GAL4TF::URA3 pGAL1-NDT80::TRP1* |
| Y3299 | *MAT α his3::hisG leu2::hisG trp1::hisG lys2 ura3 ho::LYS2*  *MAT* ***a*** *his3::hisG leu2::hisG trp1::hisG lys2 ura3 ho::LYS2*  *rec8∆::KANMX4-Rec8-PK-TEV287::LEU2 GAL-NLS-myc-9-TEV-protease-NLS-NLS::TRP1*  *rec8∆::KANMX4-Rec8-PK-TEV287::LEU2 GAL-NLS-myc-9-TEV-protease-NLS-NLS::TRP1*  *ER-GAL4TF::URA3 pGAL1-NDT80::TRP1*  *ER-GAL4TF::URA3 pGAL1-NDT80::TRP1* |
| Y3313 | *MATα CEN5 HIS3 his4 leu2::hisG trp1::hisG lys2 ura3 ho::LYS2*  *MATa CEN5::tetO_224_::HIS3 his3::hisG HIS4 leu2::pURA3::TetR::GFP::LEU2 trp1::hisG lys2 ura3 ho::LYS2*  *KANMX6-pCLB2-3HA-NSE4*  *KANMX6-pCLB2-3HA-NSE4* |
| Y3380 | *MAT α his3::hisG leu2::hisG trp1::hisG lys2 ura3 ho::LYS2*  *MAT* ***a*** *his3::hisG leu2::hisG trp1::hisG lys2 ura3 ho::LYS2*  *ndt80*∆*::HPHMX4 rec8*∆*::KANMX4-Rec8p-REC8-PK-TEV287::Leu2 GAL-NLS-myc-9-TEV-protease-NLS-NLS::TRP1*  *ndt80*∆*::HPHMX4 rec8*∆*::KANMX4-Rec8p-REC8-PK-TEV287::Leu2 GAL-NLS-myc-9-TEV-protease-NLS-NLS::TRP1*  *ER-GAL4TF::URA3 ubr1*∆*::NATMX6*  *ER-GAL4TF::URA3 ubr1*∆*::NATMX6* |
| Y3488 | *MAT α his3::hisG leu2::hisG trp1::hisG lys2 ura3 ho::LYS2*  *MAT* ***a*** *his3::hisG leu2::hisG trp1::hisG lys2 ura3 ho::LYS2*  *dmc1Δ::ARG4 HIS3MX6-pCLB2-3HA-NSE4*  *dmc1Δ::ARG4 HIS3MX6-pCLB2-3HA-NSE4* |
| Y3491 | *MAT α his3::hisG leu2::hisG trp1::hisG lys2 ura3 ho::LYS2*  *MAT* ***a*** *his3::hisG leu2::hisG trp1::hisG lys2 ura3 ho::LYS2*  *dmc1Δ::ARG4 HIS3MX6-pCLB2-3HA-SMC5*  *dmc1Δ::ARG4 HIS3MX6-pCLB2-3HA-SMC5* |
| Y3511 | *MAT α his3::hisG leu2::hisG trp1::hisG lys2 ura3 ho::LYS2*  *MAT* ***a*** *his3::hisG leu2::hisG trp1::hisG lys2 ura3 ho::LYS2*  *ZIP3-GFP::URA3 HPHMX4-pCLB2-3HA-NSE4*  *ZIP3-GFP::URA3 HPHMX4-pCLB2-3HA-NSE4* |
| Y3514 | *MAT α his3::hisG leu2::hisG trp1::hisG lys2 ura3 ho::LYS2*  *MAT* ***a*** *his3::hisG leu2::hisG trp1::hisG lys2 ura3 ho::LYS2*  *ZIP3-GFP::URA3 HPHMX4-pCLB2-3HA-SMC5*  *ZIP3-GFP::URA3 HPHMX4-pCLB2-3HA-SMC5* |
| Y3591 | *MAT α his3::hisG leu2::hisG trp1::hisG lys2 ura3 ho::LYS2*  *MAT* ***a*** *his3::hisG leu2::hisG trp1::hisG lys2 ura3 ho::LYS2*  *ZIP3-GFP::URA3 KANMX6-pCLB2-3HA-SGS1*  *ZIP3-GFP::URA3 KANMX6-pCLB2-3HA-SGS1* |
| Y3606 | *MAT α his3::hisG leu2::hisG trp1::hisG lys2 ura3 ho::LYS2*  *MAT* ***a*** *his3::hisG leu2::hisG trp1::hisG lys2 ura3 ho::LYS2*  *Htb1-mcherry::NATMX4 PDS1-tdToMATo::KlTRP1 his3::HIS3p-GFP-TUB1::HIS3*  *Htb1-mcherry::NATMX4 PDS1-tdToMATo::KlTRP1 his3::HIS3p-GFP-TUB1::HIS3* |
| Y3618 | *MAT α his3::hisG leu2::hisG trp1::hisG lys2 ura3 ho::LYS2*  *MAT* ***a*** *his3::hisG leu2::hisG trp1::hisG lys2 ura3 ho::LYS2*  *MUS81-9MYC::KlTRP1 ER-GAL4TF::URA3 pGAL1-NDT80::TRP1*  *MUS81-9MYC::KlTRP1 ER-GAL4TF::URA3 pGAL1-NDT80::TRP1* |
| Y3621 | *MAT α his3::hisG leu2::hisG trp1::hisG lys2 ura3 ho::LYS2*  *MAT* ***a*** *his3::hisG leu2::hisG trp1::hisG lys2 ura3 ho::LYS2*  *MUS81-9MYC::KlTRP1 HPHMX4-pCLB2-3HA-SMC5 ER-GAL4TF::URA3 pGAL1-NDT80::TRP1*  *MUS81-9MYC::KlTRP1 HPHMX4-pCLB2-3HA-SMC5 ER-GAL4TF::URA3 pGAL1-NDT80::TRP1* |
| Y3624 | *MAT α his3::hisG leu2::hisG trp1::hisG lys2 ura3 ho::LYS2*  *MAT* ***a*** *his3::hisG leu2::hisG trp1::hisG lys2 ura3 ho::LYS2*  *MUS81-9MYC::KlTRP1 HPHMX4-pCLB2-3HA-NSE4 ER-GAL4TF::URA3 pGAL1-NDT80::TRP1*  *MUS81-9MYC::KlTRP1 HPHMX4-pCLB2-3HA-NSE4 ER-GAL4TF::URA3 pGAL1-NDT80::TRP1* |
| Y3627 | *MAT α his3::hisG leu2::hisG trp1::hisG lys2 ura3 ho::LYS2*  *MAT* ***a*** *his3::hisG leu2::hisG trp1::hisG lys2 ura3 ho::LYS2*  *KANMX6-pCLB2-3HA-SMC5 Htb1-mcherry::NATMX4 PDS1-tdToMATo::KlTRP1 his3::HIS3p-GFP-TUB1-HIS3*  *KANMX6-pCLB2-3HA-SMC5 Htb1-mcherry::NATMX4 PDS1-tdToMATo::KlTRP1 his3::HIS3p-GFP-TUB1-HIS3* |
| Y3630 | *MAT α his3::hisG leu2::hisG trp1::hisG lys2 ura3 ho::LYS2*  *MAT* ***a*** *his3::hisG leu2::hisG trp1::hisG lys2 ura3 ho::LYS2*  *HPHMX-pCLB2-3HA-NSE4 Htb1-mcherry::NATMX4 PDS1-tdToMATo::KlTRP1 his3::HIS3p-GFP-TUB1-HIS3*  *HPHMX-pCLB2-3HA-NSE4 Htb1-mcherry::NATMX4 PDS1-tdToMATo::KlTRP1 his3::HIS3p-GFP-TUB1-HIS3* |
| Y3636 | *MAT α his3::hisG leu2::hisG trp1::hisG lys2 ura3 ho::LYS2*  *MAT* ***a*** *his3::hisG leu2::hisG trp1::hisG lys2 ura3 ho::LYS2*  *ZIP3-GFP::URA3 pCLB2-3HA-SGS1::KANMX6 pCLB2-3HA-NSE4::HPHMX6*  *ZIP3-GFP::URA3 pCLB2-3HA-SGS1::KANMX6 pCLB2-3HA-NSE4::HPHMX6* |
| Y3653 | *MAT α his3::hisG leu2::hisG trp1::hisG lys2 ura3 ho::LYS2*  *MAT* ***a*** *his3::hisG leu2::hisG trp1::hisG lys2 ura3 ho::LYS2*  *PDS1-MYC18::TRP1 REC8-HA3::URA3 HPHMX4-pCLB2-3HA-NSE4*  *PDS1-MYC18::TRP1 REC8-HA3::URA3 HPHMX4-pCLB2-3HA-NSE4* |
| Y3683 | *MAT α his3::hisG leu2::hisG trp1::hisG lys2 ura3 ho::LYS2*  *MAT* ***a*** *his3::hisG leu2::hisG trp1::hisG lys2 ura3 ho::LYS2*  *MMS4-myc9::KITRP1*  *MMS4-myc9::KITRP1* |
| Y3686 | *MAT α his3::hisG leu2::hisG trp1::hisG lys2 ura3 ho::LYS2*  *MAT* ***a*** *his3::hisG leu2::hisG trp1::hisG lys2 ura3 ho::LYS2*  *MMS4-myc9::KITRP1 HIS3MX6-pCLB2-3HA-NSE4*  *MMS4-myc9::KITRP1 HIS3MX6-pCLB2-3HA-NSE4* |
| Y3689 | *MAT α his3::hisG leu2::hisG trp1::hisG lys2 ura3 ho::LYS2*  *MAT* ***a*** *his3::hisG leu2::hisG trp1::hisG lys2 ura3 ho::LYS2*  *MMS4-myc9::KITRP1 HIS3MX6-pCLB2-3HA-SMC5*  *MMS4-myc9::KITRP1 HIS3MX6-pCLB2-3HA-SMC5* |
| Y3842 | *MAT α HIS4::LEU2-(BamHI;+ori) leu2::hisG ura3∆(sma-pst) ho::hisG*  *MAT* ***a*** *his4-X::LEU2-(NgoMIV;+ori)--URA3 leu2::hisG ura3∆(sma-pst) ho::hisG*  *ndt80∆::KANMX4 KANMX6-pCLB2-3HA-NSE4*  *ndt80∆::KANMX4 KANMX6-pCLB2-3HA-NSE4* |
| Y3843 | *MAT α HIS4::LEU2-(BamHI;+ori) leu2::hisG ura3∆(sma-pst) ho::hisG*  *MAT* ***a*** *his4-X::LEU2-(NgoMIV;+ori)--URA3 leu2::hisG ura3∆(sma-pst) ho::hisG*  *ndt80∆::KANMX4 KANMX6-pCLB2-3HA-SMC5*  *ndt80∆::KANMX4 KANMX6-pCLB2-3HA-SMC5* |
| Y3869 | *MAT α CEN5 his3::hisG leu2::hisG trp1::hisG lys2 ura3 ho::LYS2*  *MAT* ***a*** *CEN5::tetO_224_::HIS3 his3::hisG leu2::pURA3::TetR::GFP::LEU2 trp1::hisG lys2 ura3 ho::LYS2*  *spo11-Y135F-HA::URA3*  *spo11-Y135F-HA::URA3* |
| Y3870 | *MAT α CEN5 his3::hisG leu2::hisG trp1::hisG lys2 ura3 ho::LYS2*  *MAT* ***a*** *CEN5::tetO_224_::HIS3 his3::hisG leu2::pURA3::TetR::GFP::LEU2 trp1::hisG lys2 ura3 ho::LYS2*  *HPHMX4-pCLB2-3HA-SMC5 spo11-Y135F-HA::URA3*  *HPHMX4-pCLB2-3HA-SMC5 spo11-Y135F-HA::URA3* |
| Y4012 | *MAT α CEN5 his3::hisG leu2::hisG trp1::hisG lys2 ura3 ho::LYS2*  *MAT* ***a*** *CEN5::tetO_224_::HIS3 his3::hisG leu2::pURA3::TetR::GFP::LEU2 trp1::hisG lys2 ura3 ho::LYS2*  *KANMX6-pCLB2-3HA-NSE4 spo11-Y135F-HA::URA3*  *KANMX6-pCLB2-3HA-NSE4 spo11-Y135F-HA::URA3* |
| Y4041 | *MAT α his3::hisG leu2::hisG trp1::hisG lys2 ura3 ho::LYS2*  *MAT* ***a*** *his3::hisG leu2::hisG trp1::hisG lys2 ura3 ho::LYS2*  *HPHMX4-pCLB2-3HA-SMC5-IAA17::KANMX6 OsTIR1-9Myc::URA3 Htb1-mcherry::NATMX4 CDC14-GFP::LEU2*  *HPHMX4-pCLB2-3HA-SMC5-IAA17::KANMX6 OsTIR1-9Myc::URA3 Htb1-mcherry::NATMX4 CDC14-GFP::LEU2*  *his3::HIS3p-GFP-TUB1::HIS3*  *his3::HIS3p-GFP-TUB1::HIS3* |
| Y4179 | *MAT α HIS4::LEU2-(BamHI;+ori) leu2::hisG ura3∆(sma-pst) ho::hisG*  *MAT* ***a*** *his4-X::LEU2-(NgoMIV;+ori)--URA3 leu2::hisG ura3∆(sma-pst) ho::hisG*  *KANMX6-pCLB2-3HA-NSE4 KANMX6-pCLB2-3HA-SMC5*  *KANMX6-pCLB2-3HA-NSE4 KANMX6-pCLB2-3HA-SMC5* |
| Y4185 | *MAT α HIS4::LEU2-(BamHI;+ori) leu2::hisG ura3∆(sma-pst) ho::hisG*  *MAT* ***a*** *his4-X::LEU2-(NgoMIV;+ori)--URA3 leu2::hisG ura3∆(sma-pst) ho::hisG*  *KANMX4-pCLB2-3HA-SGS1 KANMX4-pCLB2-3HA-MMS4 slx4*∆*::HphMX4 yen1*∆*::HphMX4*  *KANMX4-pCLB2-3HA-SGS1 KANMX4-pCLB2-3HA-MMS4 slx4*∆*::HphMX4 yen1*∆*::HphMX4* |
| Y4188 | *MAT α HIS4::LEU2-(BamHI;+ori) leu2::hisG ura3∆(sma-pst) ho::hisG*  *MAT* ***a*** *his4-X::LEU2-(NgoMIV;+ori)--URA3 leu2::hisG ura3∆(sma-pst) ho::hisG*  *ndt80∆::KANMX4 KANMX6-pCLB2-3HA-NSE4 KANMX4-pCLB2-3HA-MMS4 slx4*∆*::HphMX4 yen1*∆*::HphMX4*  *ndt80∆::KANMX4 KANMX6-pCLB2-3HA-NSE4 KANMX4-pCLB2-3HA-MMS4 slx4*∆*::HphMX4 yen1*∆*::HphMX4* |
| Y4202 | *MAT α CEN5 his3::hisG leu2::hisG trp1::hisG lys2 ura3 ho::LYS2*  *MAT* ***a*** *CEN5::tetO_224_::HIS3 his3::hisG leu2::pURA3::TetR::GFP::LEU2 trp1::hisG lys2 ura3 ho::LYS2*  *HPHMX4-pCLB2-3HA-SMC5 KANMX6-pCLB2-3HA-NSE4 spo11-Y135F-HA::URA3 Cdc14-mcherry::NATMX4*  *HPHMX4-pCLB2-3HA-SMC5 KANMX6-pCLB2-3HA-NSE4 spo11-Y135F-HA::URA3 Cdc14-mcherry::NATMX4*  *CNM67-3mCherry::NATMX4*  *CNM67-3mCherry::NATMX4* |
| Y4540 | *MAT α his3::hisG leu2::hisG trp1::hisG lys2 ura3 ho::LYS2*  *MAT* ***a*** *his3::hisG leu2::hisG trp1::hisG lys2 ura3 ho::LYS2*  *SMC5-IAA17-3V5::KANMX6 OsTIR1-9Myc::URA3*  *SMC5-IAA17-3V5::KANMX6 OsTIR1-9Myc::URA3* |
| Y4567 | *MAT α his3::hisG leu2::hisG trp1::hisG lys2 ura3 ho::LYS2*  *MAT* ***a*** *his3::hisG leu2::hisG trp1::hisG lys2 ura3 ho::LYS2*  *CLB3-3HA::KANMX6 CLB1-13MYC::TRP1*  *CLB3-3HA::KANMX6 CLB1-13MYC::TRP1* |
| Y4570 | *MAT α his3::hisG leu2::hisG trp1::hisG lys2 ura3 ho::LYS2*  *MAT* ***a*** *his3::hisG leu2::hisG trp1::hisG lys2 ura3 ho::LYS2*  *CLB3-3HA::KANMX6 CLB1-13MYC::TRP1 HPHMX4-pCLB2-3HA-SMC5*  *CLB3-3HA::KANMX6 CLB1-13MYC::TRP1 HPHMX4-pCLB2-3HA-SMC5* |
| Y4573 | *MAT α his3::hisG leu2::hisG trp1::hisG lys2 ura3 ho::LYS2*  *MAT* ***a*** *his3::hisG leu2::hisG trp1::hisG lys2 ura3 ho::LYS2*  *CLB3-3HA::KANMX6 CLB1-13MYC::TRP1 HPHMX4-pCLB2-3HA-NSE4*  *CLB3-3HA::KANMX6 CLB1-13MYC::TRP1 HPHMX4-pCLB2-3HA-NSE4* |
| Y4606 | *MAT α his3::hisG leu2::hisG trp1::hisG lys2 ura3 ho::LYS2*  *MAT* ***a*** *his3::hisG leu2::hisG trp1::hisG lys2 ura3 ho::LYS2*  *dmc1Δ::ARG4 HIS3MX6-pCLB2-3HA-NSE4 fpr3∆::TRP1*  *dmc1Δ::ARG4 HIS3MX6-pCLB2-3HA-NSE4 fpr3∆::TRP1* |
| Y4607 | *MAT α his3::hisG leu2::hisG trp1::hisG lys2 ura3 ho::LYS2*  *MAT* ***a*** *his3::hisG leu2::hisG trp1::hisG lys2 ura3 ho::LYS2*  *rec8*∆*::HPHMX4*  *rec8*∆*::HPHMX4* |
| Y4610 | *MAT α his3::hisG leu2::hisG trp1::hisG lys2 ura3 ho::LYS2*  *MAT* ***a*** *his3::hisG leu2::hisG trp1::hisG lys2 ura3 ho::LYS2*  *hop2∆::HIS3 HIS3MX6-pCLB2-3HA-SMC5*  *hop2∆::HIS3 HIS3MX6-pCLB2-3HA-SMC5* |
| Y4613 | *MAT α his3::hisG leu2::hisG trp1::hisG lys2 ura3 ho::LYS2*  *MAT* ***a*** *his3::hisG leu2::hisG trp1::hisG lys2 ura3 ho::LYS2*  *hop2∆::HIS3 HIS3MX6-pCLB2-3HA-NSE4*  *hop2∆::HIS3 HIS3MX6-pCLB2-3HA-NSE4* |
| Y4616 | *MAT α his3::hisG leu2::hisG trp1::hisG lys2 ura3 ho::LYS2*  *MAT* ***a*** *his3::hisG leu2::hisG trp1::hisG lys2 ura3 ho::LYS2*  *hop2∆::HIS3 HIS3MX6-pCLB2-3HA-NSE4 fpr3∆::TRP1*  *hop2∆::HIS3 HIS3MX6-pCLB2-3HA-NSE4 fpr3∆::TRP1* |
| NHY1296 | *MAT α HIS4::LEU2-(BamHI;+ori) leu2::hisG ura3∆(sma-pst) ho::hisG*  *MAT* ***a*** *his4-X::LEU2-(NgoMIV;+ori)--URA3 leu2::hisG ura3∆(sma-pst) ho::hisG* |
| NHY1829 | *MAT α HIS4::LEU2-(BamHI;+ori) leu2::hisG ura3∆(sma-pst) ho::hisG*  *MAT* ***a*** *his4-X::LEU2-(NgoMIV;+ori)--URA3 leu2::hisG ura3∆(sma-pst) ho::hisG*  *mlh3*∆*::KanMX4*  *mlh3*∆*::KanMX4* |
| NHY2137 | *MAT α HIS4::LEU2-(BamHI;+ori) leu2::hisG ura3∆(sma-pst) ho::hisG*  *MAT* ***a*** *his4-X::LEU2-(NgoMIV;+ori)--URA3 leu2::hisG ura3∆(sma-pst) ho::hisG*  *KANMX4-pCLB2-3HA-MMS4*  *KANMX4-pCLB2-3HA-MMS4* |
| NHY2242 | *MAT α HIS4::LEU2-(BamHI;+ori) leu2::hisG ura3∆(sma-pst) ho::hisG*  *MAT* ***a*** *his4-X::LEU2-(NgoMIV;+ori)--URA3 leu2::hisG ura3∆(sma-pst) ho::hisG*  *KANMX4-pCLB2-3HA-SGS1*  *KANMX4-pCLB2-3HA-SGS1* |
| NHY3912 | *MAT α HIS4::LEU2-(BamHI;+ori) leu2::hisG ura3∆(sma-pst) ho::hisG*  *MAT* ***a*** *his4-X::LEU2-(NgoMIV;+ori)--URA3 leu2::hisG ura3∆(sma-pst) ho::hisG*  *KANMX4-pCLB2-3HA-MMS4 slx4*∆*::HphMX4 yen1*∆*::HphMX4*  *KANMX4-pCLB2-3HA-MMS4 slx4*∆*::HphMX4 yen1*∆*::HphMX4* |
| NHY4639 | *MAT α HIS4::LEU2-(BamHI;+ori) leu2::hisG ura3∆(sma-pst) ho::hisG*  *MAT* ***a*** *his4-X::LEU2-(NgoMIV;+ori)--URA3 leu2::hisG ura3∆(sma-pst) ho::hisG*  *KANMX6-pCLB2-3HA-NSE4*  *KANMX6-pCLB2-3HA-NSE4* |
| NHY4863 | *MAT α HIS4::LEU2-(BamHI;+ori) leu2::hisG ura3∆(sma-pst) ho::hisG*  *MAT* ***a*** *his4-X::LEU2-(NgoMIV;+ori)--URA3 leu2::hisG ura3∆(sma-pst) ho::hisG*  *KANMX6-pCLB2-3HA-NSE4 KANMX4-pCLB2-3HA-SGS1*  *KANMX6-pCLB2-3HA-NSE4 KANMX4-pCLB2-3HA-SGS1* |
| NHY5014 | *MAT α HIS4::LEU2-(BamHI;+ori) leu2::hisG ura3∆(sma-pst) ho::hisG*  *MAT* ***a*** *his4-X::LEU2-(NgoMIV;+ori)--URA3 leu2::hisG ura3∆(sma-pst) ho::hisG*  *KANMX6-pCLB2-3HA-NSE4 mnd1*∆*::KanMX4*  *KANMX6-pCLB2-3HA-NSE4 mnd1*∆*::KanMX4* |
| NHY5095 | *MAT α HIS4::LEU2-(BamHI;+ori) leu2::hisG ura3∆(sma-pst) ho::hisG*  *MAT* ***a*** *his4-X::LEU2-(NgoMIV;+ori)--URA3 leu2::hisG ura3∆(sma-pst) ho::hisG*  *KANMX6-pCLB2-3HA-NSE4 mlh3*∆*::KanMX4*  *KANMX6-pCLB2-3HA-NSE4 mlh3*∆*::KanMX4* |
| NHY5281 | *MAT α HIS4::LEU2-(BamHI;+ori) leu2::hisG ura3∆(sma-pst) ho::hisG*  *MAT* ***a*** *his4-X::LEU2-(NgoMIV;+ori)--URA3 leu2::hisG ura3∆(sma-pst) ho::hisG*  *KANMX6-pCLB2-3HA-NSE4 KANMX4-pCLB2-3HA-MMS4 slx4*∆*::HphMX4 yen1*∆*::HphMX4*  *KANMX6-pCLB2-3HA-NSE4 KANMX4-pCLB2-3HA-MMS4 slx4*∆*::HphMX4 yen1*∆*::HphMX4* |
| NHY5292 | *MAT α HIS4::LEU2-(BamHI;+ori) leu2::hisG ura3∆(sma-pst) ho::hisG*  *MAT* ***a*** *his4-X::LEU2-(NgoMIV;+ori)--URA3 leu2::hisG ura3∆(sma-pst) ho::hisG*  *KANMX6-pCLB2-3HA-SMC5*  *KANMX6-pCLB2-3HA-SMC5* |
| NHY5327 | *MAT α HIS4::LEU2-(BamHI;+ori) leu2::hisG ura3∆(sma-pst) ho::hisG*  *MAT* ***a*** *his4-X::LEU2-(NgoMIV;+ori)--URA3 leu2::hisG ura3∆(sma-pst) ho::hisG*  *KANMX6-pCLB2-3HA-SMC5 mlh3*∆*::KanMX4*  *KANMX6-pCLB2-3HA-SMC5 mlh3*∆*::KanMX4* |
| NHY5337 | *MAT α HIS4::LEU2-(BamHI;+ori) leu2::hisG ura3∆(sma-pst) ho::hisG*  *MAT* ***a*** *his4-X::LEU2-(NgoMIV;+ori)--URA3 leu2::hisG ura3∆(sma-pst) ho::hisG*  *KANMX6-pCLB2-3HA-SMC5 KANMX4-pCLB2-3HA-SGS1*  *KANMX6-pCLB2-3HA-SMC5 KANMX4-pCLB2-3HA-SGS1* |
| H118 | *MAT α his4X::LEU2-URA3 leu2::hisG lys2 ura3 ho::LYS2 arg4-Bgl II dmc1Δ::ARG4*  *MAT* ***a*** *his4B::LEU2 leu2::hisG lys2 ura3 ho::LYS2 arg4-nsp dmc1Δ::ARG4* |
| H4471 | *MAT α his4B::LEU2 leu2::hisG lys2 ura3 ho::LYS2 arg4-Bgl II REC8-HA3::URA3*  *MAT* ***a*** *his4B::LEU2 leu2::hisG lys2 ura3 ho::LYS2 arg4-Bgl II REC8-HA3::URA3* |
| H5492 | *MAT α his4X::LEU2-(Bam)-URA3 leu2::hisG lys2 ura3 ho::LYS2 SMC5-13MYC::KANMX6*  *MAT* ***a*** *his4B::LEU2 leu2::hisG lys2 ura3 ho::LYS2 SMC5-13MYC::KANMX6* |
| H6671 | *MAT α his4X::LEU2-(Bam)-URA3 leu2::hisG lys2 ura3 ho::LYS2 SMC5-3V5::KANMX6*  *MAT* ***a*** *his4B::LEU2 leu2::hisG lys2 ura3 ho::LYS2 SMC5-3V5::KANMX6* |
| H6675 | *MAT α his4B::LEU2 leu2::hisG lys2 ura3 ho::LYS2 arg4-Bgl II SMC5-3V5::KANMX6 spo11Δ::URA3*  *MAT* ***a*** *his4B::LEU2 leu2::hisG lys2 ura3 ho::LYS2 arg4-Bgl II SMC5-3V5::KANMX6 spo11Δ::URA3* |
| SG492 | *MAT α his3::hisG leu2::hisG trp1::hisG lys2 ura3 ho::LYS2 dmc1∆::HYG*  *MAT* ***a*** *his3::hisG leu2::hisG trp1::hisG lys2 ura3 ho::LYS2 dmc1∆::HYG* |
| SG481 | *MAT α his3::hisG leu2::hisG trp1::hisG lys2 ura3 ho::LYS2 dmc1∆::HYG KANMX6-pCLB2-3HA-NSE4*  *MAT* ***a*** *his3::hisG leu2::hisG trp1::hisG lys2 ura3 ho::LYS2 dmc1∆::HYG KANMX6-pCLB2-3HA-NSE4* |
| SG478 | *MAT α his3::hisG leu2::hisG trp1::hisG lys2 ura3 ho::LYS2 dmc1∆::HYG KANMX6-pCLB2-3HA-SMC5*  *MAT* ***a*** *his3::hisG leu2::hisG trp1::hisG lys2 ura3 ho::LYS2 dmc1∆::HYG KANMX6-pCLB2-3HA-SMC5* |
| SG488 | *MAT α his3::hisG leu2::hisG trp1::hisG lys2 ura3 ho::LYS2 RAD50S::URA3*  *MAT* ***a*** *his3::hisG leu2::hisG trp1::hisG lys2 ura3 ho::LYS2 RAD50S::URA3* |
| SG484 | *MAT α his3::hisG leu2::hisG trp1::hisG lys2 ura3 ho::LYS2 RAD50S::URA3 KANMX6-pCLB2-3HA-NSE4*  *MAT* ***a*** *his3::hisG leu2::hisG trp1::hisG lys2 ura3 ho::LYS2 RAD50S::URA3 KANMX6-pCLB2-3HA-NSE4* |
| SG491 | *MAT α his3::hisG leu2::hisG trp1::hisG lys2 ura3 ho::LYS2 RAD50S::URA3 KANMX6-pCLB2-3HA-SMC5*  *MAT* ***a*** *his3::hisG leu2::hisG trp1::hisG lys2 ura3 ho::LYS2 RAD50S::URA3 KANMX6-pCLB2-3HA-SMC5* |

^1^All strains used were generated in the SK-1 background.
